# Application of Comparative Transcriptional Genomics to Identify Molecular Targets for Pediatric IBD

**DOI:** 10.3389/fimmu.2015.00165

**Published:** 2015-04-08

**Authors:** Kai Fang, Matthew B. Grisham, Christopher G. Kevil

**Affiliations:** ^1^Division of Digestive Diseases, Inflammatory Bowel Disease Center, David Geffen School of Medicine at UCLA, Los Angeles, CA, USA; ^2^Department of Immunology and Molecular Microbiology, Texas Tech University Health Sciences Center, Lubbock, TX, USA; ^3^Department of Pathology, Louisiana State University Health Sciences Center, Shreveport, LA, USA; ^4^Department of Molecular and Cellular Physiology, Louisiana State University Health Sciences Center, Shreveport, LA, USA

**Keywords:** interferon regulatory factor, chemokines, chitinase 3-like 1, transcription, bioinformatics

## Abstract

Experimental models of colitis in mice have been used extensively for analyzing the molecular events that occur during inflammatory bowel disease (IBD) development. However, it is uncertain to what extent the experimental models reproduce features of human IBD. This is largely due to the lack of precise methods for direct and comprehensive comparison of mouse and human inflamed colon tissue at the molecular level. Here, we use global gene expression patterns of two sets of pediatric IBD and two mouse models of colitis to obtain a direct comparison of the genome signatures of mouse and human IBD. By comparing the two sets of pediatric IBD microarray data, we found 83 genes were differentially expressed in a similar manner between pediatric Crohn’s disease and ulcerative colitis. Up-regulation of the chemokine (C–C motif) ligand 2 (CCL2) gene that maps to 17q12, a confirmed IBD susceptibility loci, indicates that our comparison study can reveal known genetic associations with IBD. In comparing pediatric IBD and experimental colitis microarray data, we found common signatures amongst them including: (1) up-regulation of CXCL9 and S100A8; (2) cytokine–cytokine receptor pathway dysregulation; and (3) over-represented IRF1 and IRF2 transcription binding sites in the promoter region of up-regulated genes, and HNF1A and Lhx3 binding sites were over-represented in the promoter region of the down-regulated genes. In summary, this study provides a comprehensive view of transcriptome changes between different pediatric IBD populations in comparison with different colitis models. These findings reveal several new molecular targets for further study in the regulation of colitis.

## Introduction

Ulcerative colitis (UC) and Crohn’s disease (CD) are the two major forms of inflammatory bowel disease (IBD). The incidence rate of pediatric CD in the US is 43 per 100,000 and 28 per 100,000 for pediatric UC (1). As recently reported, the incidence and prevalence of pediatric IBD is rising in both developed and developing countries (2). Growth retardation poses a significant threat to the quality of life of 15–40% of children and adolescents with IBD (3). Although environmental factors, microbes in the gastroenterological tract, genetic susceptibility, and immune system dysfunction have been implicated, the etiology of pediatric IBD remains incompletely understood.

During the development of IBD, the colon tissue changes its genome transcription in response to pathological conditions, which is a result of dysregulated interaction between the immune system and enteric bacteria. The common feature of UC and CD inflamed tissue genome transcription provides new clues for pediatric IBD treatment. Although the microarray assay has been performed on pediatric IBD, there is no comprehensive genome transcription analysis for pediatric UC or CD. Here, we performed transcriptome analysis using two sets of pediatric IBD microarray data (4, 5), T-cell transfer colitis model microarray data (6), and dextran sodium sulfate (DSS)-induced colitis microarray data (7) generated from our laboratory and deposited in the National Center for Biotechnology Information Gene Expression Omnibus (NCBI GEO) database. Network and promoter analysis was performed to identify differentially expressed genes in the inflamed colon tissue from pediatric IBD patients versus experimental animal models. Comparison between pediatric IBD and experimental colitis microarray data revealed the similarly expressed genes and over-represented transcription factor binding sites (TFBS) in the promoter regions of the dysregulated genes.

## Materials and Methods

### Pediatric IBD microarray datasets

To get a comprehensive view of the pediatric IBD genome transcription profile, two sets of pediatric IBD microarray data were selected from NCBI. Those two sets of microarray data were obtained by using Affymetrix GeneChip Human Genome HG-U133 plus 2.0 arrays that provide the most comprehensive coverage of transcribed human genome and contain probes for approximately 22,634 genes. The microarray data were generated from pediatric colon in healthy controls, colon only CD, and colon only UC. The dataset GSE10616 contained data from 11 control samples, 14 CD samples, and 10 UC samples (4); the dataset GSE9686 contained data from 8 control samples, 11 CD samples, and 5 UC samples (5). Colon RNA was isolated from biopsies obtained from patients and healthy controls at diagnosis. The pediatric Crohn’s Disease Activity Index (PCDAI) and Pediatric Ulcerative Colitis Clinical Activity Index (PUCAI) were used to assess the clinical severity of the IBD sample.

### GeneSifter analysis

Two sets of pediatric IBD microarray data were uploaded to GeneSifter software^1^ and normalized for comparison by Robust Multichip Average (RMA) method. The gene expression difference threshold was set to 2 with no upper limit. Data were analyzed with a Student’s *t*-test followed by a Benjamini and Hochberg post test to limit false discovery rates, as we previously reported (7).

### Ingenuity pathway analysis

To see the relationship between differentially expressed genes, the selected genes identified as dysregulated in pediatric CD and pediatric UC microarray data were then imported to IPA^2^ for network analysis. Genes that were related to each other in biological functions and/or diseases were organized into networks according to the Ingenuity Pathways Knowledge Base (IPKB). IPKB is a database derived from the data mining of the expression of and functional relationships between molecules; this information was extracted from published papers found in NCBI PubMed, Medline, and several other databases.

### Cis-regulatory elements analysis

To identify common properties of promoter regions of differentially expressed genes, the Affymetrix gene ID of identified genes in pediatric UC and CD were uploaded to the cREMaG system^3^ (8). The sequence upstream the transcription start site (TSS) is 5000 base pairs, and the downstream sequence of TSS is 1000 base pairs. Promoter sequences were scanned with TFBS matrices obtained from the JASPAR database and the public release of the TRANSFAC database using the TFBS BioPerl module (9, 10). The top 10 of the most over-represented binding sites were selected for comparison analysis.

### Comparison between pediatric IBD and experimental colitis microarray data

By directly comparing differential gene expression between human and mouse inflamed colon tissue, we assessed the similarity between human and mouse colitis. The dysregulated gene in DSS-colitis (GEO data base accession number GSE22307) and T-cell transfer colitis model (accession number GSE27302) were divided into eight classes according to the genes expression trends. In the DSS-colitis model, there were 1609 genes that were significantly altered during the colitis development, with 501 progressively up-regulated genes and 173 progressively down-regulated genes (7). In the T-cell transfer colitis model, there were 1775 gene expressions that were significantly changed, with 341 progressively up-regulated genes and 361 progressively down-regulated genes (6). The two sets of microarray data were obtained by using the same platform, Mouse Genome 430 2.0 Array (Affymetrix), which provided the most comprehensive annotated coverage of the mouse genome, composing of over 34,000 well-characterized mouse genes. The genes whose expression progressively changed were correlated with inflammation development and were selected for promoter binding sites analysis. The over-presented promoter binding sites were further compared with the over-presented binding sites obtained from pediatric IBD array data.

## Results

### GeneSifter analysis pediatric IBD microarray data

Analysis of GSE 9686 pediatric CD microarray data showed that 242 genes were differentially expressed, 173 genes had up-regulated expression, while 69 genes had down-regulated expression. Analysis of the GSE 10616 pediatric CD microarray data showed that there were 298 genes differentially expressed (the expression of 209 genes were up-regulated and 89 genes were down-regulated). After comparing two sets of pediatric CD microarray data, we found the expression of 167 genes was similarly changed. Among those 167 genes, 117 genes were up-regulated (Table S1 in Supplementary Material), and 50 gene expressions were down-regulated (Table S2 in Supplementary Material).

In GSE9686 pediatric UC microarray data, there were 3860 genes differentially expressed (1717 genes were up-regulated, and 2143 genes were down-regulated). While in GSE10616 pediatric UC, there were 1826 genes differentially expressed (1122 genes were up-regulated and 704 genes were down-regulated). After comparing the two sets of pediatric UC data, we found that 1071 genes were similarly up-regulated (Table S3 in Supplementary Material), and 736 genes were down-regulated (Table S4 in Supplementary Material).

**Figure 1:**
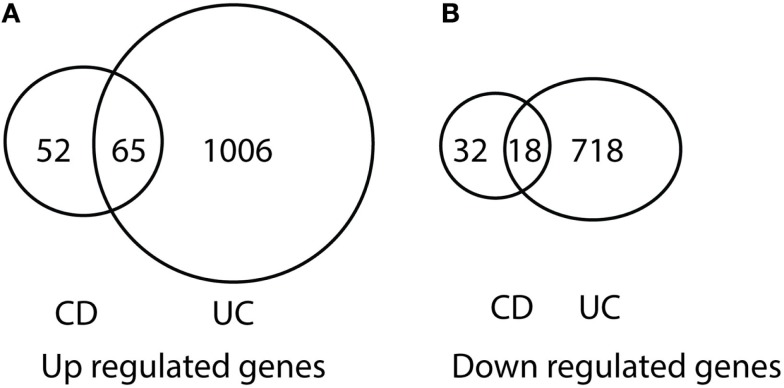
**Venn diagram illustration of gene expression similarity between pediatric CD and UC patient sample microarray data**. **(A)** One-hundred seventeen genes were up-regulated from pediatric CD compared with 1071 up-regulated genes from pediatric UC patients, with 65 genes being common between the two groups. **(B)** Fifty genes were down-regulated from pediatric CD compared from 736 down-regulated gene from pediatric UC patients, with 18 genes being common between the two groups.

After comparing the data in Tables S1 and S3 in Supplementary Material, we found that there were 65 genes up-regulated in pediatric CD and pediatric UC, as shown in Table 1 and Figure 1A. By comparing Tables S2 and S4 in Supplementary Material, we found that there were 18 genes down-regulated in pediatric CD and pediatric UC, as shown in Table 2 and Figure 1B.

**Table 1:** **Similarly up-regulated genes in pediatric IBD**.

Gene ID	Gene identifier	GSE9686 CD	GSE10616 CD	GSE9686UC	GSE10616UC
ACSL4	NM_022977	2.52	2.38	5.46	4.19
ADM	NM_001124	2.19	2.25	3.70	3.17
ALDH1A2	AB015228	2.22	2.93	11.29	8.36
APCDD1	N48299	2.3	2.28	3.45	2.94
C4BPA	NM_000715	2.01	2.50	10.31	5.94
CCL2	S69738	2.37	2.40	3.16	2.73
CCRL1	NM_016557	2.04	2.13	5.18	3.13
CDH11	D21254	2.52	2.34	6.21	4.08
CFB	NM_001710	2.25	2.27	3.44	3.19
CFI	BC020718	2.04	2.10	6.36	3.38
CH25H	NM_003956	2.01	2.02	3.66	2.99
CHI3L1	M80927	6.15	5.72	33.46	21.81
CLDN1	AF101051	2.17	2.54	8.57	4.59
COL1A2	NM_000089	2.02	2.51	5.01	4.08
COL4A1	NM_001845	2.03	2.09	4.27	3.65
COL6A3	NM_004369	2.07	2.35	4.24	3.97
CXCL1	NM_001511	5.60	4.66	18.35	13.43
CXCL11	AF002985	6.82	5.61	14.19	12.26
CXCL2	M57731	3.35	3.39	10.29	9.44
CXCL3	NM_002090	2.89	3.27	9.44	8.29
CXCL5	AK026546	6.04	6.49	44.64	22.12
CXCL6	NM_002993	4.84	5.97	30.42	18.36
CXCL9	NM_002416	4.70	3.23	4.13	3.95
CYP27B1	NM_000785	2.03	2.19	2.35	2.22
CYR61	NM_001554	3.11	2.98	3.69	3.94
DERL3	AI655697	2.25	2.11	5.50	3.96
DUOX2	NM_014080	11.06	9.73	29.14	25.35
DUOXA2	AI821606	2.24	3.79	14.00	13.6
EMR2	NM_013447	2.55	2.54	5.01	4.20
FCGR1A	X14355	2.82	2.39	2.88	2.61
FCRL5	AF343662	2.47	2.53	6.18	4.60
FN1	X02761	2.08	2.07	2.15	2.32
HSD11B1	NM_005525	2.68	2.57	4.12	3.84
IGKV1D-13	AW408194	3.19	2.52	6.96	4.87
IGLV1-44	U96394	3.63	3.26	15.57	9.51
IVD	AF043583	3.00	3.08	11.48	7.58
KDELR3	NM_006855	2.38	2.50	5.09	4.13
LCN2	NM_005564	3.11	3.27	7.12	6.18
LOXL2	NM_002318	2.23	2.31	4.77	3.78
LPL	BF672975	2.19	2.73	6.44	4.22
MMP1	NM_002421	5.44	6.69	41.57	22.03
MMP10	NM_002425	3.71	4.86	44.39	34.11
MMP3	NM_002422	11.65	10.85	66.73	49.68
MS4A2	NM_000139	2.33	2.12	3.73	2.96
NEBL	NM_006393	2.24	2.29	3.94	3.24
NIACR2	NM_006018	4.90	4.04	7.94	8.03
NID1	BF940043	2.39	2.44	5.00	3.61
NOS2	L24553	2.12	2.02	4.07	3.54
NTN3	AF103529	3.64	2.91	9.44	6.10
PCDH7	NM_002589	2.07	2.16	7.21	4.27
PCSK1	NM_000439	3.25	4.54	11.66	8.33
PHLDA1	AA576961	2.89	2.91	9.53	6.48
PSAT1	BC004863	3.49	2.49	9.33	5.50
S100A8	NM_002964	6.09	5.66	21.7	17.55
SEC24D	NM_014822	2.35	2.19	4.85	3.49
SGMS1	AI377497	2.07	2.17	5.15	3.56
SLC6A14	NM_007231	7.11	10.32	56.81	51.22
SOCS3	AI244908	2.36	2.55	4.49	4.88
SOD2	W46388	2.29	2.31	3.86	3.03
SPINK4	NM_014471	6.02	5.83	17.04	11.85
TFPI2	L27624	3.68	3.22	10.00	7.01
TIMP1	NM_003254	2.29	2.34	5.44	4.89
TMEM158	BF062629	2.46	3.06	8.01	5.83
TMEM45A	NM_018004	2.40	2.67	9.10	5.01
TYRP1	NM_000550	2.34	2.79	6.58	5.63

*The two set of pediatric microarray data are downloaded from NCBI Gene Expression Omnibus (NCBI GEO) database. Values are mean fold change normalized to control*.

**Table 2:** **Similarly down-regulated genes in pediatric IBD**.

Gene ID	Gene identifier	GSE9686 CD	GSE10616 CD	GSE9686UC	GSE10616UC
ABCB1	AF016535	−2.13	−2.02	−4.98	−4.04
ABCG2	AF098951	−4.93	−3.09	−8.52	−9.93
APOBEC3B	NM_004900	−2.09	−2.05	−2.78	−2.80
AQP8	NM_001169	−4.57	−3.85	−28.58	−36.55
KRT12	NM_000223	−2.53	−2.22	−4.34	−3.69
LOC389023	AI499651	−2.07	−2.81	−3.56	−3.60
LOC643008	BF478120	−2.02	−2.00	−4.50	−2.58
MEP1B	NM_005925	−5.08	−3.82	−6.37	−8.09
PCDH21	AI825832	−2.15	−2.24	−6.15	−3.70
PHLPPL	AB023148	−2.13	−2.01	−4.29	−3.99
PLA2G12B	BF939574	−2.29	−2.36	−2.80	−3.73
PRAP1	AA502331	−2.93	−2.33	−3.99	−4.65
SGK2	AI631895	−2.6	−2.92	−4.76	−4.35
SLC16A9	BG401568	−4.05	−3.08	−6.67	−4.65
SLC17A4	NM_005495	−2.64	−2.58	−8.08	−6.66
SLC23A3	AI263078	−2.61	−2.27	−5.24	−3.98
SLC3A1	M95548	−3.11	−2.43	−3.25	−4.76
WSCD1	AB011095	−2.17	−2.05	−2.62	−2.73

*The two set of pediatric microarray data are downloaded from NCBI Gene Expression Omnibus (NCBI GEO) database. Values are mean fold change normalized to control*.

Of the up-regulated genes, seven were from the CXC chemokine family: CXCL1, CXCL2, CXCL3, CXCL5, CXCL6, CXCL9, and CXCL11, which are the key components of the cytokine–cytokine receptor interaction pathway. CXCL1 is expressed by epithelial cells, macrophages, and neutrophils (11, 12) and has neutrophil chemoattractant activity (13). CXCL2 is secreted by macrophages and monocytes and is a chemoattractant for polymorphonuclear cells, leukocytes, and hematopoietic stem cells (11, 14, 15). CXCL5 is expressed in eosinophils and stimulates the chemotaxis of neutrophils (16). CXCL6 is a chemoattractant for neutrophils (17). CXCL9 is an interferon (IFN)-dependent CXC chemokine, which plays a pro-inflammatory role and has been found to be expressed at high levels in UC tissue (18). CXCL11 is a chemoattractant for activated T cells (19).

Of the down-regulated genes, four were solute carrier genes: SLC16A9, SLC17A4, SLC23A3, and SLC3A1. The functions of those down-regulated genes related to the transport of amino acids (20), monocarboxylate (21), glutamate (22), sodium-phosphate (23), and ascorbic acid (24).

### Ingenuity analysis of pediatric IBD microarray data

Those genes differentially expressed in pediatric CD and UC were uploaded to Ingenuity software for network analysis. Those differentially expressed genes in pediatric CD inflamed colon tissue were organized into eight networks. The molecules in each network and their top functions are listed in Table S5 in Supplementary Material. Those differentially expressed genes in pediatric UC inflamed colon tissue were organized into 25 networks, and the molecules in each network and their top functions are listed in Table S6 in Supplementary Material.

**Figure 2:**
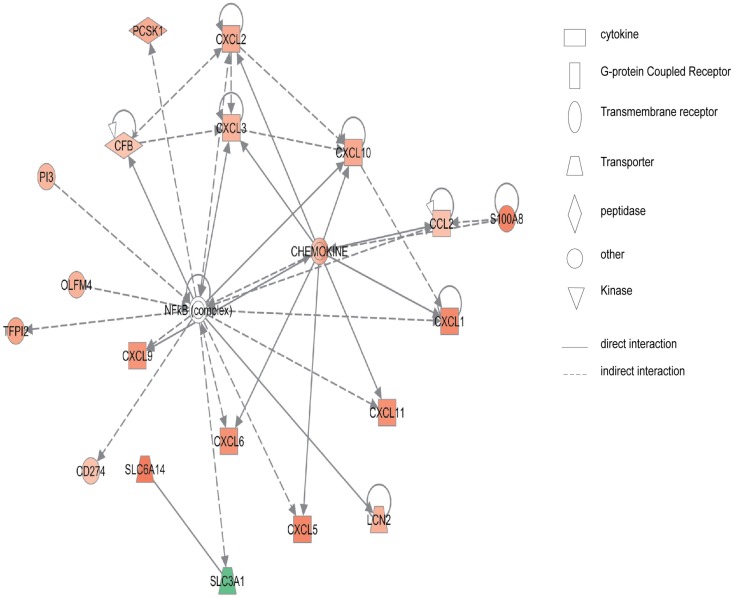
**Network 1 of pediatric CD has biological functions associated with cell-to-cell signaling and interaction, gastrointestinal disease, hepatic system disease**. Red shading indicates up-regulation, whereas green shading shows down-regulation.

**Figure 3:**
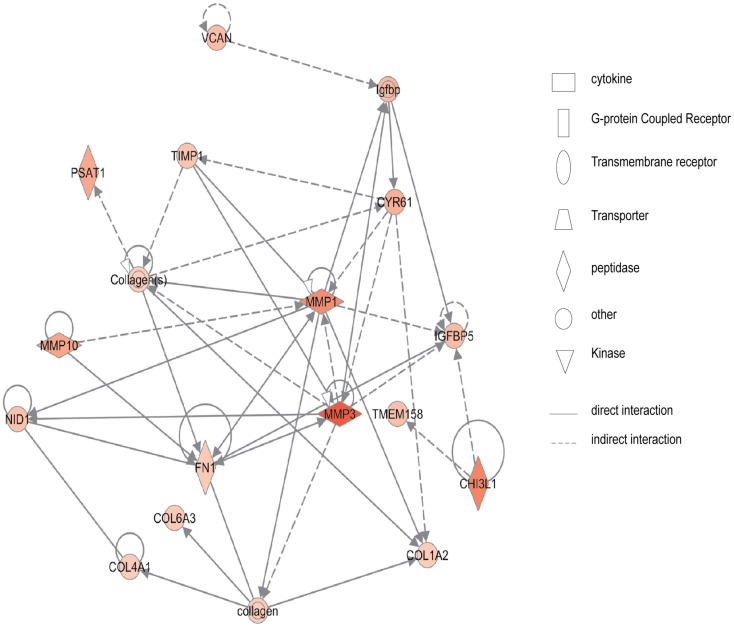
**Network 2 of pediatric CD has biological functions associated with connective tissue disorders, genetic disorder, and cardiovascular disease**. Red shading indicates up-regulation, whereas green shading shows down-regulation.

Figure 2 shows the first network of pediatric CD inflamed colon tissue differentially expressed genes with their cell-to-cell signaling functions and their interactions, as they relate to gastrointestinal and hepatic system disease. As shown in Figure 2, the transcription of nine chemokine genes was up-regulated, and those genes indirectly react with the NF-κB complex. Figure 3 shows the network 2 differentially expressed genes in pediatric CD inflamed colon tissue, which is composed of 15 up-regulated genes with functions related to connective tissue and genetic disorders. MMP-1 and MMP-3 are located in the center of pediatric CD network 2. ChI3l1 is also implicated in this network through its indirect reaction with IGFBP5.

**Figure 4:**
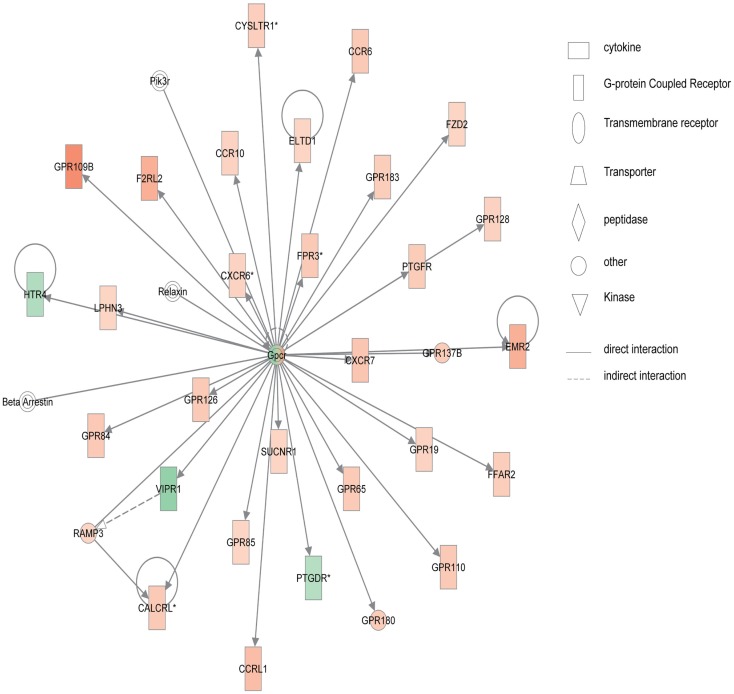
**Network 1 of pediatric UC has biological functions associated with cellular movement, cell signaling, and nucleic acid metabolism**. Red shading indicates up-regulation, whereas green shading shows down-regulation.

**Figure 5:**
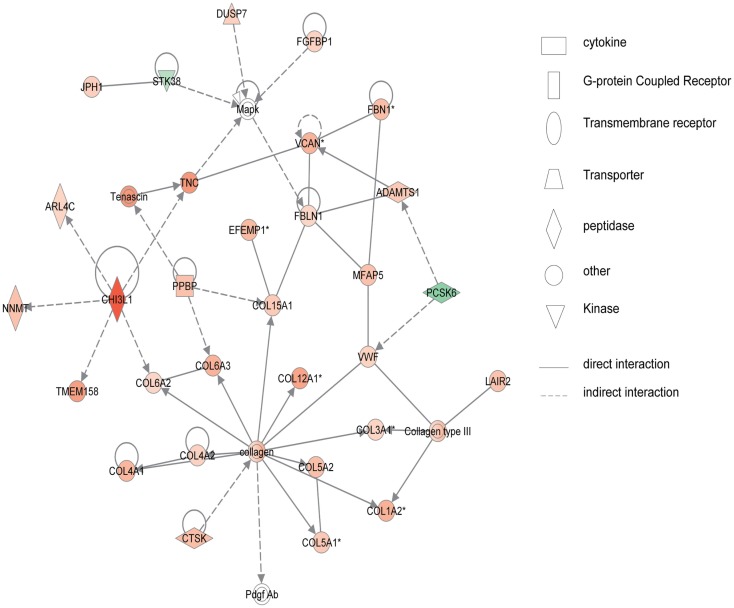
**Network 2 of pediatric UC has biological functions associated with connective tissue disorders, genetic disorder, and dermatological diseases and conditions**. Red shading indicates up-regulation, whereas green shading shows down-regulation.

Figure 4 shows the first network of pediatric UC with functions related to cellular movement and signaling. Pediatric UC network 1 is mainly composed of eleven G-protein-coupled receptors, which were all up-regulated. Transcription of eight members of the collage family was up-regulated as shown in Figure 5, with functions related to connective tissue. Interestingly, ChI3L1 was in the center of the pediatric UC network 2 (Figure 5), and ChI3L1 indirectly interacts with COL16A2 and TNC.

### Promoter analysis of pediatric IBD microarray data

Using the CREMaG system, we indentified over-presented TFBS in the differentially expressed genes. The over-presented TFBS of differentially regulated genes in pediatric CD is shown in Tables S7–S10 in Supplementary Material. TFBS over-presented in pediatric UC differentially expressed genes are shown in Table S11 in Supplementary Material (for up-regulated genes) and in Table S12 in Supplementary Material (for down-regulated genes). The fold-difference in TFBS frequency was computed by dividing the observed TFBS number by the background number.

By comparison, we found that there were six promoter sequences (RELA, NF-κB, IRF2, Evi1, and IRF1) that were over-presented in genes that were up-regulated in pediatric IBD. There were six TFBS (Lhx3, MEF2A, HNF1A, Nobox, NR2F1, and Foxa2) that were over-presented in the genes that were down-regulated in pediatric IBD-inflamed colon tissue.

In the pediatric CD microarray data analysis, the NF-κB binding sequence was over-presented in the inflammatory-related genes, such as CCL2, CXCL10, CXCL2, CXCL3, CXCL6, CXCL9, and in other up-regulated genes in the pediatric CD-inflamed colon tissue. The network analysis of pediatric CD (Figure 2) also showed that NF-κB regulates chemokine gene expression. Not surprisingly, in the pediatric UC microarray data promoter analysis, the NF-κB binding site was also shown to be over-represented in the promoter region of the up-regulated genes, as it is well known that NF-κB plays a pivotal role in the expression of inflammatory mediators. The promoter region of 89 up-regulated genes (ICAM-1, COL1A1, WNT5A, CXCL5, IL-1B, CXCL2, IL-6, IL-11, and others) in pediatric UC has the NF-κB binding site.

### Comparison of pediatric IBD and experimental colitis microarray data

The dysregulated KEGG pathway of GSE9686 pediatric UC and GSE10616 pediatric UC is shown in Tables S13 and S14 in Supplementary Material. The over-represented TFBS of progressively up-regulated or down-regulated genes in the T-cell transfer colitis model and the DSS-colitis model are shown in Tables S15–S18 in Supplementary Material.

**Figure 6:**
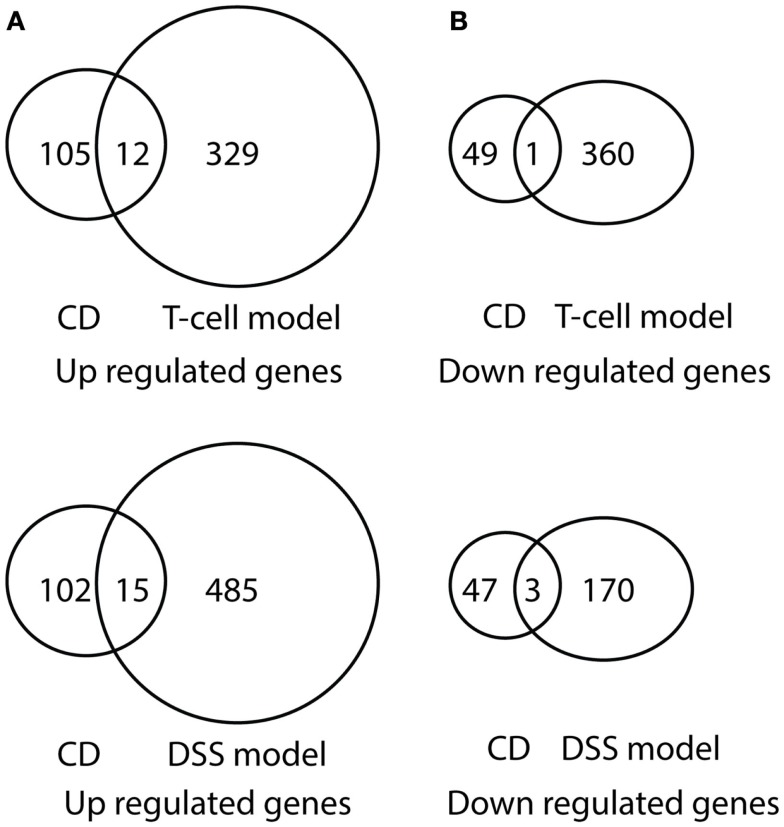
**Venn diagram illustration of gene expression similarity between pediatric CD and experimental colitis model microarray data**. **(A)** One-hundred seventeen genes were up-regulated from pediatric CD compared with 341 up-regulated genes from T-cell colitis model and 501 up-regulated genes from DSS-colitis model. **(B)** Fifty genes were down-regulated from pediatric CD compared from 361 down-regulated gene from T-cell colitis model and 173 down-regulated genes from DSS-colitis model.

**Figure 7:**
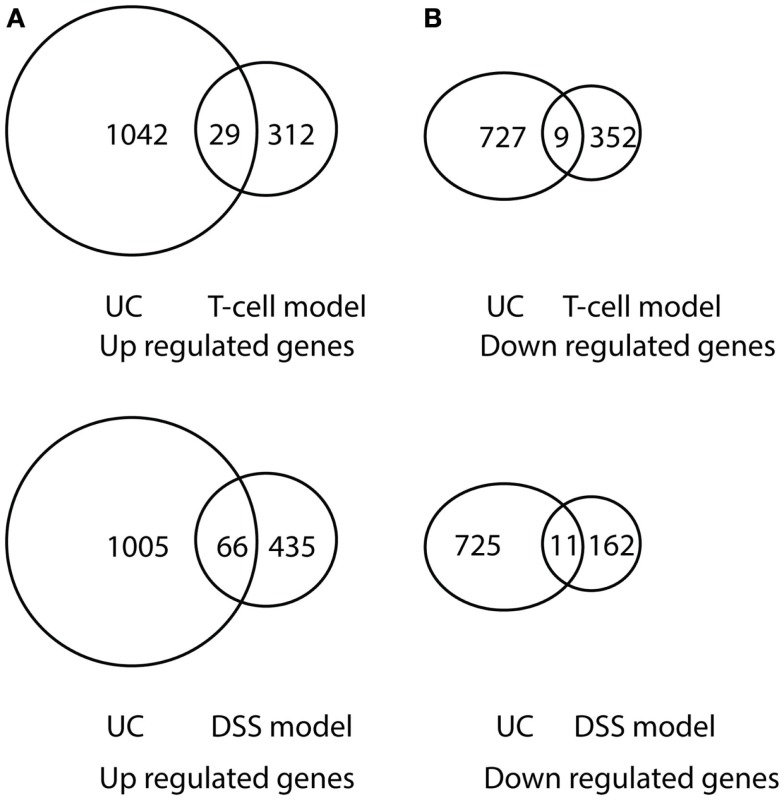
**Venn diagram illustration of gene expression similarity between pediatric UC patient sample microarray data and experimental colitis model microarray data**. **(A)** One-thousand seventy one genes were up-regulated from pediatric UC compared with 341 up-regulated genes from T-cell colitis model and 501 up-regulated genes from DSS-colitis model. **(B)** Seven-hundred thirty six genes were down-regulated from pediatric UC compared from 361 down-regulated gene from T-cell colitis model and 173 down-regulated genes from DSS-colitis model.

Comparison of differentially expressed genes in pediatric IBD and progressively up-regulated or down-regulated genes in experimental colitis is shown in Table 3 and Figures 6 and 7. The comparison of the over-presented promoters in the differentially expressed genes is shown in Table 4. Among them, the cytokine–cytokine receptor pathway was dysregulated in all the four sets of microarray data. CXCL9 and S100A8 were up-regulated in all the four sets of microarray data. The expression of S100A8 was also found to be up-regulated in the trinitrobenzene sulfonic acid (TNBS)-induced colitis rat model and T-cell-mediated colitis in SCID mice (25, 26).

**Table 3:** **Common dysregulated pathways and genes**.

	Dysregulated pathways	Genes up-regulated	Genes down-regulated
T-cell model vs. pediatric CD	Cytokine–cytokine receptor interaction, chemokine signaling pathway, Leishmaniasis, Chagas disease, asthma, malaria, NOD-like receptor signaling pathway	CXCL1, CXCL10, CXCL2, CXCL5, CXCL9, DUOXA2, GBP1, IL-1B, MMP3, PSAT1, S100A8, TGM2	ABCB1
T-cell model vs. pediatric UC	Cytokine–cytokine receptor interaction, chemokine signaling pathway, cell adhesion molecules (CAMs), hematopoietic cell lineage, Leishmaniasis, malaria, Alzheimer’s disease, Huntington’s disease, graft-vs.-host disease	ARL4C, CTSC, CD274, CDC6, CCR1, CXCL1, CXCL2, CXCL9, CSF2RB, C1R, C2, DUOXA2, EGR2, PSAT1, PYHIN1, RUNX2, S100A8, S100A9, SLPI, SRGN, STAT1, SLAMF8, SLC7A11, SNX10, SOCS1, TGM2, WARS, UBD, ZC3H12A	ABCB1, CHKA, DENND1B, FGFR3, HOXB5, HSD3B2, RBM25, SCIN, VSIG2
DSS model vs. pediatric CD	Cytokine–cytokine receptor interaction, Toll-like receptor signaling pathway, ECM-receptor interaction	ACSL4, APCDD1, ALDH1A2, CCL2, CXCL10, CXCL9, CHI3L1, HSD11B1, IGFBP5, LCN2, MMP10, MMP3, PCDH7, S100A8, SOCS3	ANK3, AQP8, PHLPPL
DSS model vs. pediatric UC	Cytokine–cytokine receptor interaction, complement and coagulation cascades, cell adhesion molecules (CAMs), Toll-like receptor signaling pathway, ECM-receptor interaction, hematopoietic cell lineage	ACSL4, APCDD1, ALDH1A2, CTSK, CD300A, CD86, CCL2, CCL3, CCR1, CXCL9, CHRDL2, COL18A1, CSF2RB, CLEC7A, CRISPLD2, CYP1B1, EDNRA, EDNRB, EPHA3, EMP3, GPR84, GJA1, IGFBP5, ICAM-1, IFIT3, IL-11, IL33, IL-6, LMCD1, LCN2, LHFP, LUM, LCP2, MMP10, MMP12, MMP2, MMP3, NR2F1, OLFML2B, OLFML3, OSMR, PDE4B, PLXNC1, KCNJ8, PCOLCE, PSMB9, PCDH7, PYHIN1, QK1, RSPO3, S100A8, S100A9, SAMSN1, SLPI, SH3PXD2B, STAT1, SLAMF8, ST8SIA4, SOCS3, STX11, TUBB6, TNFSF11, TNFRSF11B, TWIST1, VGLL3, WISP1	ABAT, ACVR1C, ANK3, AQP8, ABCC6, MALAT1, PHLPPL, RBM25, SLC26A2, SLC26A3, TRPM6

**Table 4:** **Comparison promoter between animal model and pediatric IBD**.

	Over-represented in up-regulated genes	Over-represented in down-regulated genes
T-cell model vs. pediatric CD	IRF1	Lhx3
	IRF2	MEF2A
	NF-κB	HNF1A
	RELA	Nobox
	ELF5	
T-cell model vs. pediatric UC	IRF1	HNF1A
	IRF2	Lhx3
	NFYA	Pax4
	RELA	MEF2A
	FOXF2	IRF2
	NF-κB	Nobox
DSS model vs. pediatric CD	IRF2	Lhx3
	IRF1	HNF1A
		Foxa2
DSS model vs. pediatric UC	IRF2	HNF1A
	Pax4	Lhx3
	IRF1	Pax4
	FOXF2	IRF2
		Foxa2

Promoter analysis provided the possible common regulatory mechanism of the expression of the dysregulated genes. As shown in Table 4, the IRF1 and IRF2 binding sites were over-represented in the up-regulated genes in pediatric IBD and experimental colitis. The HNF1A and Lhx3 binding sites were over-presented in the down-regulated genes in the four sets of microarray data. HNF1A is a transcription factor that regulates the expression of cytokine-driven C-reactive protein, which is a clinical marker of inflammation (27). Lhx3 is a transcription factor that is required for pituitary and motor neuron development (28).

As shown in Table 3, cytokine–cytokine receptor interaction pathway is dysregulated in pediatric UC, CD, T-cell transfer, and DSS-colitis model. While chemokine signaling pathway is dysregulated in pediatric UC, CD, and T-cell transfer colitis model, but not in DSS-colitis model. Additionally, NF-κB binding site is over-presented in the promoter region of up-regulated genes in pediatric CD, UC, and T-cell transfer colitis, but not in DSS-colitis model (Table 4). Thus, the comparison of dysregulated KEGG pathway (Table 3) and the over-represented TFBS (Table 4) showed that the T-cell transfer colitis model was better than the DSS-induced colitis model at simulating pediatric IBD; however, the DSS-colitis model was more similar to pediatric UC than pediatric CD, as the DSS model has more common dysregulated pathways and molecules (Table 3) and over-represented TFBS in the dysregulated genes with pediatric UC than CD (Table 4).

## Discussion

Our microarray analysis revealed that chitinase 3-like 1 (cartilage glycoprotein-39, CHI3L1) was up-regulated in pediatric IBD samples. CHI3L1 has the ability to enhance the adhesion and internalization of bacteria in epithelial cells (29). *In vivo*, neutralizing CHI3L1 with an antibody suppresses DSS-induced colitis, and this neutralization dramatically decreases bacteria adhesion and invasion of epithelial cells. It has been demonstrated that CHI3L1 expression is up-regulated in epithelial cells under inflammatory conditions. CHI3L1 also activates Akt signaling in epithelial cells through its chitin binding motif, and increases secretion of IL-8 and TNF-α in a dose-dependent manner (30). Fecal CHI3L1 levels are positively correlated with pathology score (31). Serum concentration of CHI3L1 is also elevated in IBD patients (32). Thus, CHI3L1 might be selected as both a target and a marker of pediatric IBD.

Cysteine-rich, angiogenic inducer 61 (CYR61 or CCN1) was up-regulated in pediatric IBD. It has been demonstrated that CCN1 up-regulates pro-inflammatory gene transcription, such as TNF-α, IL-1α, IL-1β, IL-6, and IL-12b in mice macrophages (33). This induction results from CCN1 direct activation of NF-κB and increased TNF-α synthesis. CCN1 supports macrophage adhesion through integrin αMβ2 and syndecan-4. Because mice lacking CCN1 cannot develop *in utero*, involving vascular defects in the placenta, CCN1 is also related with vasculogenesis during embryogenesis (34). Moreover, CCN1 construct transfected mice showed increased angiogenesis in colon tissue (35). Together, these suggest that CCN1 might be a unique target to treat pediatric IBD through inhibition of pro-inflammatory gene expression and angiogenesis.

Chemokine (C–C motif) ligand 2 (CCL2), also known as monocyte chemotactic protein-1 or small inducible cytokine A2, was up-regulated in pediatric inflamed colon tissue. CCL2 attracts monocytes, memory T cells, and dendritic cells to sites of tissue injury, infection, and inflammation (36, 37). Interestingly, CCL2 is located in the confirmed CD and UC susceptibility loci 19q12 (38, 39). Increased expression of CCL2 (Table 1) in pediatric inflamed colon tissue supports the idea that CCL2 might be one of the causal genes of pediatric IBD. Interestingly, this has been demonstrated by nanomolar concentrations of CCL2 stimulating inflammatory responses of monocytes and effector T cells, whereas picomolar CCL2 exerts a global suppressive effect on T-cell trafficking into inflamed lymph nodes (40), as confirmed by picomolar levels of CCL2 ameliorating TNBS- and DSS-induced colitis (41). Thus, before targeting CCL2 for IBD therapy more information is needed regarding its dose effect on human colon tissue inflammation responses.

One major difference of genome transcription in adult IBD versus pediatric IBD colon tissue is that there is fewer common dysregulated genes found in adult IBD. Feng et al. found that 25 genes were up-regulated and 18 genes were down-regulated in adult IBD inflamed colon tissue (42). We found that 65 genes were up-regulated and 18 genes were down-regulated in pediatric IBD colon tissue. Compared with those two studies, CXCL2 and CXCL3 were both up-regulated in pediatric and adult IBD, and only ABCB1 was down-regulated in pediatric and adult IBD. This comparison suggests that there is a large difference between pediatric and adult IBD patients. Additionally, both studies show that there is a difference between CD and UC genome transcription patterns, which suggests that CD and UC have distinctive pathogenesis.

Promoter analysis provided potential targets at the transcription factor level. NF-κB transcription factors are comprised of five family members in mammalian cells: RelA (p65), RelB, c-Rel, p50/p105 (NF-κB1), and p52/p100 (NF-κB2). Those members form homo- or hetero-dimers of NF-κB complexes to regulate the expression of a variety of genes (43). Interestingly, Stronati et al. found that nuclear NF-κB and the binding activity of NF-κB to a consensus DNA sequence were significantly increased in the inflamed mucosa of patients, compared to controls (44). In IBD patients, the increased NF-κB expression in mucosal macrophages is accompanied with an increasing capacity of these cells to produce and secrete TNF-α, IL-1, and IL-6 (45). Many of the established immunosuppressive drugs for IBD, like corticosteroids, play anti-inflammatory roles, at least partly via the inhibition of the NF-κB activity (46). The fact that the NF-κB binding sites are over-represented in the T-cell transfer colitis model, combined with the fact that the NF-κB binding sites are over-represented in pediatric IBD inflamed colon tissue suggests that NF-κB likely plays an important role in pediatric IBD. In agreement with this idea, NF-κB has been extensively studied, and different ways to block NF-κB have been evaluated for IBD treatment. Unfortunately, due to significant side effects and liver toxicity, optimal ways to block NF-κB to treat IBD has not been realized (47).

Interferon regulatory factor-1 and -2 (IRF1 and IRF2) are transcription factors that regulate expression of inflammatory-related genes, but are primarily identified as transcription factors which regulate the human IFN-α/β gene (48). Interestingly, our promoter analysis showed that binding site of IRF1 and IFR2 are over-represented in the pediatric IBD up-regulated genes. Additionally, Clavell et al. found increased expression of IRF1 in lamina propria mononuclear cells from patients with CD (49). Compared with wild-type mice, production of TNF-α and IFN-γ in IRF1^−/−^ mice is greatly impaired (50). Mice with a target mutation in IRF2 (IRF2^−/−^) exhibit significant inhibition of IL-12, IL-12R, IFN-γ, IL-1 β, and IL-6 expression (51). It has been demonstrated that IRF2 recruits the NF-κB transcription factor into the nucleus via physical interaction, which enhances TNF-α-induced NF-κB transcription (52). Thus, IRF1 and IRF2 have the potential to be selective and potentially effective targets for the treatment of both experimental colitis and pediatric IBD.

In conclusion, we performed pediatric IBD transcriptome analysis and its cross-species comparison with experimental colitis models. Identification of common dysregulated gene expression profiles, over-represented transcription binding sites, and related transcription factors controlling dysregulated gene expression changes reveal several molecular targets that serve as novel pathways for further study and potential therapy for pediatric IBD.

## Conflict of Interest Statement

The authors declare that the research was conducted in the absence of any commercial or financial relationships that could be construed as a potential conflict of interest.

## Supplementary Material

The Supplementary Material for this article can be found online at http://journal.frontiersin.org/article/10.3389/fimmu.2015.00165/abstract

## Footnotes

^1^http://www.genesifter.com

^2^http://www.ingenuity.com

^3^http://www.cremag.org
