# Supplementary material for: Application of Comparative Transcriptional Genomics to Identify Molecular Targets for Pediatric IBD
Source: Front Immunol. 2015 Apr 8;6:165. doi: 10.3389/fimmu.2015.00165 (PMC4457140; doi:10.3389/fimmu.2015.00165)
Supplement: Supplementary file 1 [file Data_Sheet1.PDF]

Supplementary Table 1. Common genes that are up regulated in pediatric CD

Gene Title	Gene ID	Gene Identifier	Other ID	GSE9686		GSE10616	
				fold value	p	Fold value	p
Acyl-CoA synthetase long-chain family member 4	ACSL4	NM_022977	202422_s_at	2.52	0.02	2.38	0.01
Adenomatosis polyposis coli down-regulated 1	APCDD1	N48299	225016_at	2.3	0.01	2.28	0.01
Adrenomedullin	ADM	NM_001124	202912_at	2.19	0.01	2.25	0.01
Aldehyde dehydrogenase 1 family, member A2	ALDH1A2	AB015228	207016_s_at	2.22	0.02	2.93	0.01
Ankyrin repeat domain 22	ANKRD22	AI097229	239196_at	2.28	0.01	2.05	0.01
Anti-thyroglobulin light chain variable region	-	X79782	217179_x_at	2.84	0.03	2.6	0.01
Cadherin 11, type 2, OB-cadherin (osteoblast)	CDH11	D21254	207173_x_at	2.52	0.02	2.34	0.02
CDNA FLJ13585 fis, clone PLACE1009150	-	AU157716	227051_at	2.38	0.01	2.52	0.01
CDNA FLJ14388 fis, clone HEMBA1002716	-	AA147884	229802_at	3.23	0.01	3.3	0.01
CDNA FLJ38396 fis, clone FEBRA2007957	-	R55784	242488_at	2.17	0.01	2.39	0.01
Chemokine (C-C motif) ligand 2	CCL2	S69738	216598_s_at	2.37	0.03	2.4	0.01
Chemokine (C-C motif) receptor-like 1	CCRL1	NM_016557	220351_at	2.04	0.02	2.13	0.02
Chemokine (C-X-C motif) ligand 1 (melanoma growth stimulating activity, alpha)	CXCL1	NM_001511	204470_at	5.6	0.01	4.66	0.01
Chemokine (C-X-C motif) ligand 10	CXCL10	NM_001565	204533_at	3.45	0.01	2.74	0.03
Chemokine (C-X-C motif) ligand 11	CXCL11	AF002985	211122_s_at	6.82	0.01	4.36	0.01
Chemokine (C-X-C motif) ligand 2	CXCL2	M57731	209774_x_at	3.35	0.01	3.39	0.01
Chemokine (C-X-C motif) ligand 3	CXCL3	NM_002090	207850_at	2.89	0.01	3.27	0.01
Chemokine (C-X-C motif) ligand 5	CXCL5	AK026546	214974_x_at	6.04	0.02	6.49	0.01
Chemokine (C-X-C motif) ligand 6 (granulocyte chemotactic protein 2)	CXCL6	NM_002993	206336_at	4.84	0.02	5.97	0.01
Chemokine (C-X-C motif) ligand 9	CXCL9	NM_002416	203915_at	4.7	0.01	3.23	0.02
Chitinase 3-like 1 (cartilage glycoprotein-39)	CHI3L1	M80927	209395_at	6.15	0.01	5.45	0.01
Cholesterol 25-hydroxylase	CH25H	NM_003956	206932_at	2.01	0.01	2.02	0.01
Claudin 1	CLDN1	AF101051	222549_at	2.17	0.01	2.54	0.01
Clone 24405 mRNA sequence	-	AA530995	213832_at	2.54	0.01	2.61	0.01

Collagen, type I, alpha 2	COL1A2	NM_000089	202404_s_at	2.02	0.02	2.51	0.01
Collagen, type IV, alpha 1	COL4A1	NM_001845	211981_at	2.03	0.03	2.09	0.01
Collagen, type VI, alpha 3	COL6A3	NM_004369	201438_at	2.07	0.02	2.35	0.01
Complement component 4 binding protein, alpha	C4BPA	NM_000715	205654_at	2.01	0.02	2.5	0.01
Complement factor B	CFB	NM_001710	202357_s_at	2.25	0.01	2.27	0.01
Complement factor I	CFI	BC020718	1555564_a_at	2.04	0.03	2.1	0.02
Consensus includes gb:AI694413 /FEA=EST /DB_XREF=gi:4971753 /DB_XREF=est:wd83d12.x1 /CLONE=IMAGE:2338199 /UG=Hs.332649 olfactory receptor, family 2, subfamily I, member 6	-	AI694413	235229_at	2.46	0.01	2.28	0.03
Cysteine-rich, angiogenic inducer, 61	CYR61	NM_001554	201289_at	3.11	0.01	2.98	0.01
Cytochrome P450, family 27, subfamily B, polypeptide 1	CYP27B1	NM_000785	205676_at	2.03	0.01	2.19	0.01
Der1-like domain family, member 3	DERL3	AI655697	229721_x_at	2.25	0.01	2.11	0.01
Dual oxidase 2	DUOX2	NM_014080	219727_at	11.1	0.01	9.73	0.01
Dual oxidase maturation factor 2	DUOXA2	AI821606	230615_at	2.24	0.01	3.79	0.01
Egf-like module containing, mucin-like, hormone receptor-like 2	EMR2	NM_013447	207610_s_at	2.55	0.03	2.54	0.01
Elongation factor, RNA polymerase II, 2	ELL2	AI924426	226099_at	2.61	0.01	2.58	0.01
Family with sequence similarity 20, member B	FAM20B	BG540628	214768_x_at	4.4	0.01	3.2	0.01
Fc fragment of IgG, high affinity Ia, receptor (CD64)	FCGR1A	X14355	216950_s_at	2.82	0.02	2.39	0.02
Fc fragment of IgG, high affinity Ib, receptor (CD64)	FCGR1B	L03419	214511_x_at	3.77	0.01	3.06	0.01
Fc fragment of IgG, low affinity IIIb, receptor (CD16b)	FCGR3B	NM_000570	204006_s_at	4.11	0.01	2.95	0.02
Fc receptor-like 5	FCRL5	AF343662	224404_s_at	2.47	0.01	2.53	0.01
Fibronectin 1	FN1	X02761	212464_s_at	2.08	0.02	2.07	0.01
FK506 binding protein 11, 19 kDa	FKBP11	NM_016594	219118_at	2.97	0.01	2.51	0.01
Glutathione peroxidase 8 (putative)	GPX8	AL571557	227628_at	2.92	0.01	2.61	0.01
Guanylate binding protein 1, interferon-inducible, 67kDa	GBP1	NM_002053	202270_at	2.66	0.01	2.32	0.01
Heat shock protein 70kDa family, member 13	HSPA13	NM_006948	202558_s_at	2.32	0.01	2.08	0.01
hemoglobin, beta	-	M25079	209116_x_at	2.53	0.01	3.24	0.01

Hydroxysteroid (11-beta) dehydrogenase 1	HSD11B1	NM_005525	205404_at	2.68	0.01	2.57	0.01
Hypothetical protein MGC29506	MGC29506	AF151024	223565_at	2.53	0.02	2.33	0.01
IKK interacting protein	IKIP	AW182575	227295_at	2.19	0.02	2.05	0.02
Immunoglobulin (mAb56) light chain V region mRNA, partial sequence	-	D84140	217235_x_at	3.01	0.03	2.96	0.01
immunoglobulin heavy constant alpha 1 /// immunoglobulin heavy constant delta /// immunoglobulin heavy constant gamma 1 (G1m marker) /// immunoglobulin heavy constant mu /// immunoglobulin heavy variable 3-23 /// immunoglobulin heavy variable 4-31	-	AB035175	216510_x_at	2.98	0.01	2.69	0.01
immunoglobulin heavy constant delta	-	AJ275469	214973_x_at	3.16	0.01	2.25	0.02
Immunoglobulin heavy constant gamma 1 (G1m marker)	IGHG1	BC001872	209374_s_at	3.19	0.02	3.69	0.01
immunoglobulin heavy constant mu	-	U80139	216491_x_at	2.86	0.02	2.55	0.01
immunoglobulin heavy locus /// immunoglobulin heavy constant alpha 1 /// immunoglobulin heavy constant gamma 1 (G1m marker) /// immunoglobulin heavy constant gamma 3 (G3m marker) /// immunoglobulin heavy constant mu /// immunoglobulin heavy variable	-	AJ225092	211868_x_at	2.18	0.03	2.17	0.01
Immunoglobulin kappa chain, V-region (SPK.3)	-	AF103530	217157_x_at	2.52	0.03	2.19	0.01
Immunoglobulin kappa light chain (IGKV) mRNA variable region, joining region, and constant region	-	M85256	211645_x_at	4.24	0.02	3.14	0.01
immunoglobulin kappa variable 1/OR2-108 (non-functional)	-	X51887	217378_x_at	2.87	0.03	2.38	0.01
Immunoglobulin kappa variable 1D-13	IGKV1D-13	AW408194	216207_x_at	3.19	0.02	2.52	0.01
immunoglobulin lambda locus	-	D87021	216560_x_at	2.84	0.01	2.11	0.03
Immunoglobulin lambda locus	IGL@	AB001733	211798_x_at	2.65	0.03	2.57	0.01

Immunoglobulin lambda locus	IGL@	AB014341	211881_x_at	2.03	0.03	2.06	0.01
Immunoglobulin lambda locus	IGL@	AJ249377	217148_x_at	3.15	0.03	3.17	0.01
Immunoglobulin lambda locus	IGL@	D01059	211655_at	2.19	0.01	2.22	0.01
Immunoglobulin lambda locus	IGL@	D84143	216984_x_at	2.9	0.02	2.7	0.01
Immunoglobulin lambda locus	IGL@	X93006	217227_x_at	2.49	0.02	2.34	0.01
Immunoglobulin lambda variable 1-44	IGLV1-44	U96394	234764_x_at	3.63	0.02	3.26	0.01
Insulin-like growth factor binding protein 5	IGFBP5	AW157548	203424_s_at	2.22	0.01	2.46	0.01
Interleukin 1, beta	IL1B	NM_000576	205067_at	3.32	0.01	3.37	0.01
Interleukin 8	IL8	NM_000584	202859_x_at	6.5	0.01	3.37	0.02
Isolate 09 immunoglobulin light chain variable region	-	BG482805	214777_at	4.49	0.01	3.46	0.01
Isovaleryl Coenzyme A dehydrogenase	IVD	AF043583	217258_x_at	3	0.02	3.08	0.01
KDEL (Lys-Asp-Glu-Leu) endoplasmic reticulum protein retention receptor 3	KDELR3	NM_006855	204017_at	2.38	0.01	2.5	0.01
Lipocalin 2	LCN2	NM_005564	212531_at	3.11	0.01	3.27	0.01
Lipoprotein lipase	LPL	BF672975	203548_s_at	2.19	0.01	2.73	0.01
Lysyl oxidase-like 2	LOXL2	NM_002318	202998_s_at	2.23	0.02	2.31	0.01
Matrix metallopeptidase 1 (interstitial collagenase)	MMP1	NM_002421	204475_at	5.44	0.02	6.69	0.01
Matrix metallopeptidase 10 (stromelysin 2)	MMP10	NM_002425	205680_at	3.71	0.01	4.86	0.01
Matrix metallopeptidase 3 (stromelysin 1, progelatinase)	MMP3	NM_002422	205828_at	11.7	0.01	10.85	0.01
Membrane-spanning 4-domains, subfamily A, member 2 (Fc fragment of IgE, high affinity I, receptor for; beta polypeptide)	MS4A2	NM_000139	207496_at	2.33	0.03	2.12	0.02
Nebulette	NEBL	NM_006393	203962_s_at	2.24	0.01	2.29	0.01
Netrin 3	NTN3	AF103529	216576_x_at	3.64	0.03	2.91	0.02
Niacin receptor 2	NIACR2	NM_006018	205220_at	4.9	0.02	4.04	0.01
Nidogen 1	NID1	BF940043	202007_at	2.39	0.02	2.44	0.01
Nitric oxide synthase 2, inducible	NOS2	L24553	210037_s_at	2.12	0.01	2.02	0.01
olfactomedin 4	-	AL390736	212768_s_at	2.93	0.02	2.24	0.02
peptidase inhibitor 3, skin-derived	-	L10343	41469_at	2.76	0.01	3.89	0.01
Phosphoserine aminotransferase 1	PSAT1	BC004863	223062_s_at	3.49	0.01	2.49	0.01
Pleckstrin homology-like domain, family A, member 1	PHLDA1	AA576961	217996_at	2.89	0.01	2.91	0.02
Proprotein convertase	PCSK1	NM_000439	205825_at	3.25	0.01	4.54	0.01

subtilisin/kexin type 1								
Prostaglandin-endoperoxide synthase 2 (prostaglandin G/H synthase and cyclooxygenase)	PTGS2	AY151286	1554997_a_at	2.31	0.02	3.75	0.01
Protocadherin 7	PCDH7	NM_002589	205534_at	2.07	0.01	2.16	0.01
Regenerating islet-derived family, member 4	REG4	AY126671	1554436_a_at	2.95	0.01	3.75	0.01
S100 calcium binding protein A8	S100A8	NM_002964	202917_s_at	6.09	0.02	5.66	0.01
SEC24 family, member D (S. cerevisiae)	SEC24D	NM_014822	202375_at	2.35	0.01	2.19	0.01
Serine peptidase inhibitor, Kazal type 4	SPINK4	NM_014471	207214_at	6.02	0.01	5.83	0.01
similar to Ig kappa chain V-I region HK102 precursor	-	AJ408433	216401_x_at	3.4	0.02	2.72	0.01
SLAM family member 7	-	AL121985	222838_at	2.84	0.02	2.23	0.01
Solute carrier family 6 (amino acid transporter), member 14	SLC6A14	NM_007231	219795_at	7.11	0.01	10.32	0.01
Sphingomyelin synthase 1	SGMS1	AI377497	212989_at	2.07	0.01	2.17	0.01
Stearoyl-CoA desaturase (delta-9-desaturase)	SCD	AB032261	200832_s_at	2.53	0.01	2.14	0.01
Superoxide dismutase 2, mitochondrial	SOD2	W46388	215223_s_at	2.29	0.01	2.31	0.01
Suppressor of cytokine signaling 3	SOCS3	AI244908	227697_at	2.36	0.02	2.55	0.01
TIMP metallopeptidase inhibitor 1	TIMP1	NM_003254	201666_at	2.29	0.03	2.34	0.01
Tissue factor pathway inhibitor 2	TFPI2	L27624	209278_s_at	3.68	0.01	3.22	0.01
Transcribed locus	-	AI401105	240118_at	2.44	0.01	2.11	0.01
Transcribed locus	-	AI608902	227458_at	2.3	0.02	2.29	0.01
Transcribed locus	-	BF513674	235965_at	2.49	0.01	2.29	0.01
transglutaminase 2 (C polypeptide, protein-glutamine-gamma-glutamyltransferase)	-	AL031651	201042_at	2.47	0.01	2.52	0.01
Transmembrane protein 158	TMEM158	BF062629	213338_at	2.46	0.02	3.06	0.01
Transmembrane protein 45A	TMEM45A	NM_018004	219410_at	2.4	0.02	2.67	0.01
Tyrosinase-related protein 1	TYRP1	NM_000550	205694_at	2.34	0.01	2.79	0.01
Vanin 1	VNN1	NM_004666	205844_at	3.81	0.01	6.22	0.01
Versican	VCAN	NM_004385	204620_s_at	2.7	0.02	2.58	0.01

Supplementary Table 2. Common genes that are down regulated in pediatric CD

Gene Title	Gene ID	Gene Identifier	Other ID	GSE9686		GSE10616	
				fold	p	fold	p
Ankyrin 3, node of Ranvier (ankyrin G)	ANK3	AL136710	209442_x_at	-2.02	0.01	-2.62	0.01
Apolipoprotein B mRNA editing enzyme, catalytic polypeptide-like 3B	APOBEC3B	NM_004900	206632_s_at	-2.09	0.01	-2.05	0.03
Aquaporin 8	AQP8	NM_001169	206784_at	-4.57	0.03	-3.85	0.01
ATP-binding cassette, sub-family B (MDR/TAP), member 1	ABCB1	AF016535	209994_s_at	-2.13	0.01	-2.02	0.02
ATP-binding cassette, sub-family C (CFTR/MRP), member 3	ABCC3	AF009670	209641_s_at	-2.11	0.01	-2.06	0.02
ATP-binding cassette, sub-family G (WHITE), member 2	ABCG2	AF098951	209735_at	-4.93	0.01	-3.09	0.03
CDNA FLJ12288 fis, clone MAMMA1001783	-	AK022350	233388_at	-2.16	0.01	-2.1	0.01
CDNA FLJ13274 fis, clone OVARC1001029	-	AU155112	234095_at	-2.02	0.01	-2.07	0.01
CDNA: FLJ21389 fis, clone COL03455	-	AK025042	1564323_at	-3.22	0.01	-3.52	0.01
CDNA: FLJ21391 fis, clone COL03479	-	AK025044	215657_at	-2.21	0.02	-2.56	0.01
CDNA: FLJ21565 fis, clone COL06463	-	AK025218	234193_at	-2.3	0.01	-2.35	0.01
CDNA: FLJ22614 fis, clone HSI05089	-	AK026267	234632_x_at	-2.58	0.02	-2.86	0.01
Chromosome 10 open reading frame 116	C10orf116	NM_006829	203571_s_at	-2.33	0.01	-2.37	0.01
Consensus includes gb:AI494291 /FEA=EST /DB_XREF=gi:4395294 /DB_XREF=est:qy98d11.x1 /CLONE=IMAGE:2020053 /UG=Hs.111977 ESTs	-	AI494291	240690_at	-2.12	0.01	-2.12	0.01
Homo sapiens cDNA FLJ20670 fis, clone KAIA4743.	-	AK000677	233876_at	-2.16	0.01	-2.45	0.01
Homo sapiens hypothetical protein FLJ23556 (FLJ23556), mRNA.	-	NM_024880	206548_at	-2.27	0.02	-2.09	0.03
Hydroxysteroid (17-beta) dehydrogenase 2	HSD17B2	NM_002153	204818_at	-3.06	0.02	-2.23	0.04
Hypothetical gene supported by AK128346	LOC440993	BM914560	1558220_at	-2.14	0.02	-2.11	0.01
Hypothetical gene supported by BC032913; BC048425	LOC389023	AI499651	236351_at	-2.07	0.02	-2.81	0.01
hypothetical LOC440993	-	CA418406	1557293_at	-2.55	0.01	-2.44	0.01
Keratin 12	KRT12	NM_000223	207811_at	-2.53	0.01	-2.22	0.01
Meprin A, beta	MEP1B	NM_005925	207251_at	-5.08	0.01	-3.82	0.01
peptidyl arginine deiminase, type II	-	AL049569	209791_at	-2.28	0.01	-2.14	0.01
PH domain and leucine rich repeat protein phosphatase-like	PHLPPL	AB023148	213407_at	-2.13	0.01	-2.01	0.01
Phospholipase A2, group XIIB	PLA2G12B	BF939574	231009_at	-2.29	0.02	-2.36	0.01
PP12104	LOC643008	BF478120	229740_at	-2.02	0.01	-2	0.02

Proline-rich acidic protein 1	PRAP1	AA502331	243669_s_at	-2.93	0.01	-2.33	0.01
Protocadherin 21	PCDH21	AI825832	213369_at	-2.15	0.03	-2.24	0.01
Serum/glucocorticoid regulated kinase 2	SGK2	AI631895	230573_at	-2.6	0.02	-2.92	0.01
Solute carrier family 16, member 1 (monocarboxylic acid transporter 1)	SLC16A1	NM_003051	202236_s_at	-2.04	0.02	-2.27	0.01
Solute carrier family 16, member 9 (monocarboxylic acid transporter 9)	SLC16A9	BG401568	227506_at	-4.05	0.01	-3.08	0.01
Solute carrier family 17 (sodium phosphate), member 4	SLC17A4	NM_005495	207051_at	-2.64	0.02	-2.58	0.01
Solute carrier family 20 (phosphate transporter), member 1	SLC20A1	AI671885	230494_at	-2.58	0.01	-3.68	0.01
Solute carrier family 23 (nucleobase transporters), member 3	SLC23A3	AI263078	230949_at	-2.61	0.01	-2.27	0.01
Solute carrier family 3 (cystine, dibasic and neutral amino acid transporters, activator of cystine, dibasic and neutral amin	SLC3A1	M95548	205799_s_at	-3.11	0.03	-2.43	0.04
Transcribed locus	-	AA598661	236216_at	-2.22	0.01	-2.23	0.02
Transcribed locus	-	AA682539	235575_at	-2.37	0.02	-2.75	0.01
Transcribed locus	-	AI090874	240110_at	-3.55	0.01	-3.2	0.01
Transcribed locus	-	AI424825	231484_at	-2.55	0.02	-2.24	0.02
Transcribed locus	-	AI457588	242733_at	-2.12	0.01	-2.14	0.01
Transcribed locus	-	AI610347	238999_at	-2.19	0.01	-2.01	0.01
Transcribed locus	-	AI916887	241592_at	-3.05	0.01	-2.46	0.01
Transcribed locus	-	AW117547	237157_at	-2.28	0.02	-2.23	0.02
Transcribed locus	-	AW136397	242055_at	-2.17	0.01	-2.14	0.01
Transcribed locus	-	AW971248	222372_at	-2.36	0.01	-2.35	0.02
Transcribed locus	-	AW976631	242059_at	-2.32	0.01	-2.84	0.01
Transcribed locus	-	BF590303	243462_s_at	-2.27	0.01	-2.38	0.01
Transcribed locus	-	T65568	244282_at	-2.87	0.01	-3.08	0.01
WSC domain containing 1	WSCD1	AB011095	213155_at	-2.17	0.02	-2.05	0.01
zinc finger, DHHC-type containing 11	-	AF267859	221646_s_at	-2.31	0.01	-2.11	0.02

Supplementary Table 3. Commonly up regulated genes in pediatric UC

Gene Title	Gene ID	Gene Identifier	GSE 9686		GSE10616	
			fold	P	fold	P
1-acylglycerol-3-phosphate O-acyltransferase 4 (lysophosphatidic acid acyltransferase, delta)	AGPAT4	AI733330	2.2	0.01	2.17	0.01
2,4-dienoyl CoA reductase 1, mitochondrial	-	AF049895	2.16	0.01	2	0.01
2'-5'-oligoadenylate synthetase 2, 69/71kDa	OAS2	NM_016817	2.05	0.02	2.37	0.01
5'-nucleotidase domain containing 2	NT5DC2	NM_022908	2.37	0.01	2.08	0.01
6-phosphofructo-2-kinase/fructose-2,6-biphosphatase 3	PFKFB3	NM_004566	2.96	0.01	2.63	0.01
A kinase (PRKA) anchor protein 12	AKAP12	AB003476	2.58	0.02	2.16	0.01
A kinase (PRKA) anchor protein 2	AKAP2	NM_007203	2.67	0.01	2.23	0.01
Abhydrolase domain containing 2	ABHD2	AW292816	2.66	0.01	2.03	0.01
Acid phosphatase 5, tartrate resistant	ACP5	NM_001611	2.92	0.01	2.46	0.01
Acid phosphatase, prostate	ACPP	AI659898	3.48	0.01	3.01	0.01
Acidic (leucine-rich) nuclear phosphoprotein 32 family, member E	ANP32E	NM_030920	3.21	0.01	2.27	0.01
Activin A receptor, type I	ACVR1	NM_001105	2.97	0.01	2.36	0.01
acyl-CoA synthetase long-chain family member 1	-	NM_021122	2.66	0.01	2.22	0.01
Acyl-CoA synthetase long-chain family member 4	ACSL4	NM_022977	5.46	0.01	4.19	0.01
ADAM metallopeptidase domain 12	ADAM12	NM_003474	2.12	0.01	2.02	0.01
ADAM metallopeptidase domain 19 (meltrin beta)	ADAM19	Y13786	2.38	0.02	2.24	0.01
ADAM metallopeptidase domain 9 (meltrin gamma)	ADAM9	AF495383	3.4	0.01	2.43	0.01
ADAM metallopeptidase with thrombospondin type 1 motif, 1	-	AK023795	2.87	0.01	2.58	0.01
ADAM metallopeptidase with thrombospondin type 1 motif, 12	ADAMTS12	W74476	3.6	0.01	2.74	0.01
ADAM metallopeptidase with thrombospondin type 1 motif, 5	ADAMTS5	BF060767	3.46	0.01	2.48	0.01
ADAM metallopeptidase with thrombospondin type 1 motif, 9	ADAMTS9	AI431730	2.45	0.01	2.28	0.01
Adaptor-related protein complex 1, sigma 3 subunit	AP1S3	AF393369	2.08	0.01	2.27	0.01
Adenomatosis polyposis coli down-regulated 1	APCDD1	N48299	3.45	0.01	2.94	0.01
Adenylate cyclase activating polypeptide 1 (pituitary)	ADCYAP1	BE220888	3.93	0.01	3.01	0.01

Adipocyte-specific adhesion molecule	ASAM	BF056275	2.74	0.01	2.17	0.01
ADP-ribosylation factor GTPase activating protein 3	ARFGAP3	BC005122	3.24	0.01	2.6	0.01
ADP-ribosylation factor-like 4C	ARL4C	AW450363	2.53	0.05	2.04	0.02
Adrenomedullin	ADM	NM_001124	3.7	0.01	3.17	0.01
AHNAK nucleoprotein 2	AHNAK2	AI935123	2.52	0.01	2.05	0.01
Aldehyde dehydrogenase 1 family, member A2	ALDH1A2	AB015228	11.29	0.01	8.36	0.01
Aldehyde oxidase 1	AOX1	NM_001159	3.52	0.01	2.34	0.01
aldolase B, fructose-bisphosphate	-	BC005314	5.69	0.01	3.67	0.01
Amphiregulin	AREG	NM_001657	2.46	0.01	2.01	0.01
Amyotrophic lateral sclerosis 2 (juvenile) chromosome region, candidate 4	ALS2CR4	BG194770	2.39	0.01	2.03	0.01
Angiopoietin 2	ANGPT2	AA083514	2.92	0.01	2.65	0.01
Angiopoietin-like 2	ANGPTL2	AF007150	4.12	0.01	3.5	0.01
Angiotensinogen (serpin peptidase inhibitor, clade A, member 8)	AGT	NM_000029	10.58	0.01	6.61	0.01
Anillin, actin binding protein	ANLN	AK023208	4.39	0.01	2.97	0.01
ankyrin repeat domain 22	-	AI925518	2.51	0.02	2.41	0.01
Annexin A1	ANXA1	NM_000700	5.74	0.01	4.2	0.01
Annexin A3	ANXA3	M63310	3.56	0.01	2.88	0.01
Annexin A5	ANXA5	NM_001154	2.43	0.01	2.09	0.01
Anoctamin 6	ANO6	AL041280	2.16	0.01	2.07	0.01
Anterior gradient homolog 2 (Xenopus laevis)	AGR2	AI922323	2.78	0.01	2.57	0.01
Anthrax toxin receptor 1	ANTXR1	AF279145	2.95	0.02	2.38	0.01
Anti-thyroglobulin light chain variable region	-	X79782	10.29	0.01	7.69	0.01
Apolipoprotein B mRNA editing enzyme, catalytic polypeptide-like 3F	APOBEC3F	BF508948	2.53	0.01	2.16	0.01
Apolipoprotein B mRNA editing enzyme, catalytic polypeptide-like 3G	APOBEC3G	NM_021822	3.08	0.01	2.56	0.01
Apolipoprotein L, 1	APOL1	AF323540	3.34	0.01	3.02	0.01
apolipoprotein L, 6	-	BF512806	3.09	0.01	2.2	0.01
Aquaporin 3 (Gill blood group)	AQP3	N74607	2.75	0.01	2.54	0.01
Aquaporin 9	AQP9	NM_020980	5.62	0.01	5.18	0.01
ArfGAP with SH3 domain, ankyrin repeat and PH domain 1	ASAP1	AW513835	3.08	0.01	2.64	0.01
Armadillo repeat containing, X-linked 2	ARMCX2	NM_014782	2.37	0.01	2	0.01
aryl hydrocarbon receptor nuclear translocator-like 2	-	AF256215	2.91	0.01	2.88	0.01
arylsulfatase B	-	AW168942	2.34	0.01	2.01	0.01
Aspartate beta-hydroxylase	ASPH	AW469351	3.02	0.01	2.06	0.01
Aspartate beta-hydroxylase domain containing 2	ASPHD2	BE550881	3.46	0.01	2.73	0.01

Aspartylglucosaminidase	AGA	NM_000027	2.95	0.01	2.24	0.01
Asporin	ASPN	AF316824	2.35	0.01	2.09	0.01
Ataxin 1	ATXN1	BF438383	2.53	0.01	2.07	0.01
ATPase family, AAA domain containing 2	ATAD2	NM_014109	2.78	0.01	2.11	0.01
ATPase, Ca++ transporting, type 2C, member 1	ATP2C1	AF225981	2.02	0.01	2.24	0.01
ATPase, class I, type 8B, member 2	ATP8B2	AB032963	2.57	0.03	2.04	0.01
ATP-binding cassette, sub-family C (CFTR/MRP), member 4	ABCC4	BC041560	3.09	0.01	2.26	0.01
ATP-binding cassette, sub-family G (WHITE), member 5	ABCG5	NM_022436	3.11	0.01	2.17	0.01
B cell RAG associated protein	-	NM_014863	3.38	0.01	2.73	0.01
B9 protein domain 1	B9D1	BC002944	2.73	0.01	2.29	0.01
Basic helix-loop-helix family, member e22	BHLHE22	AL134708	2.78	0.01	2.38	0.01
Basonuclin 2	BNC2	AW024890	2.6	0.01	2.34	0.01
BCL2/adenovirus E1B 19kDa interacting protein 3	-	U15174	3.75	0.01	2.8	0.01
BCL2-associated athanogene 2	BAG2	AF095192	4.23	0.01	3.36	0.01
BCL2-related protein A1	BCL2A1	NM_004049	3.42	0.01	2.9	0.01
beta-1,3-N-acetylgalactosaminyltransferase 1 (globoside blood group)	-	AF154848	2.83	0.01	2.39	0.01
Beta-site APP-cleaving enzyme 2	BACE2	AF178532	2.71	0.01	2.18	0.01
bicaudal D homolog 1 (Drosophila)	-	BC010091	2.61	0.01	2.45	0.01
Biglycan	BGN	AA845258	2.34	0.01	2.44	0.01
Biliverdin reductase A	BLVRA	AA740186	2.72	0.01	2.13	0.01
Bone morphogenetic protein 6	BMP6	NM_001718	3.23	0.01	2.5	0.01
bone morphogenetic protein 7	-	AL157414	2.22	0.01	2.11	0.01
branched chain aminotransferase 1, cytosolic	-	AL390172	4.08	0.01	2.89	0.01
Budding uninhibited by benzimidazoles 1 homolog (yeast)	BUB1	AF043294	3.4	0.01	2.56	0.01
Cadherin 11, type 2, OB-cadherin (osteoblast)	CDH11	D21254	6.21	0.01	4.08	0.01
Cadherin 13, H-cadherin (heart)	CDH13	NM_001257	2.7	0.01	2.32	0.01
Cadherin 3, type 1, P-cadherin (placental)	CDH3	NM_001793	4.2	0.01	4.16	0.01
Calcitonin receptor-like	CALCRL	NM_005795	3.26	0.01	2.52	0.01
Caldesmon 1	CALD1	NM_004342	3.9	0.01	2.86	0.01
Calumenin	CALU	AF257659	3.34	0.01	2.66	0.01
Carbohydrate (chondroitin 4) sulfotransferase 11	CHST11	AI123348	3.02	0.01	2.34	0.01
Carbohydrate (chondroitin 4) sulfotransferase 12	CHST12	NM_018641	2.55	0.01	2.12	0.01
Carbohydrate (N-acetylglucosamine-6-O)	CHST2	NM_004267	3.67	0.01	3.02	0.01

sulfotransferase 2						
Carbonyl reductase 3	CBR3	NM_001236	3.22	0.01	2.3	0.01
Carboxypeptidase A3 (mast cell)	CPA3	NM_001870	10.21	0.01	5.8	0.01
Carboxypeptidase X (M14 family), member 1	CPXM1	NM_019609	2.89	0.01	2.37	0.01
Caspase 1, apoptosis-related cysteine peptidase (interleukin 1, beta, convertase)	CASP1	U13699	2.42	0.01	2.09	0.01
Caspase recruitment domain family, member 16	CARD16	NM_052889	2.46	0.01	2.24	0.01
Caspase recruitment domain family, member 6	CARD6	AF356193	3.26	0.01	2.67	0.01
Cat eye syndrome chromosome region, candidate 1	CECR1	NM_017424	2.97	0.01	2.18	0.01
Cathepsin C	CTSC	AI246687	2.7	0.01	2.92	0.01
Cathepsin E	CTSE	NM_001910	2.89	0.02	2.45	0.01
Cathepsin K	CTSK	NM_000396	3.93	0.01	3.27	0.01
Caveolin 2	CAV2	NM_001233	3.04	0.02	2.08	0.04
CD1b molecule	CD1B	NM_001764	2.35	0.01	2.19	0.01
CD1e molecule	CD1E	AA309511	2.93	0.02	2.57	0.01
CD274 molecule	CD274	AF233516	2.13	0.01	2.06	0.01
CD300a molecule	CD300A	AF020314	2.34	0.02	2.08	0.01
CD38 molecule	CD38	NM_001775	5.03	0.01	3.8	0.01
CD44 molecule (Indian blood group)	CD44	AF098641	2.45	0.01	2.1	0.01
CD55 molecule, decay accelerating factor for complement (Cromer blood group)	-	CA448665	5.66	0.01	4.11	0.01
CD79a molecule, immunoglobulin-associated alpha	CD79A	NM_001783	2.32	0.02	2.13	0.01
CD80 molecule	CD80	BC042665	2.12	0.01	2.02	0.01
CD86 molecule	CD86	L25259	3.92	0.01	2.86	0.01
CDNA clone IMAGE:30924414	-	BF058422	4.16	0.01	3.59	0.01
CDNA clone IMAGE:4342162	-	BF512556	2.27	0.01	2.09	0.01
CDNA clone IMAGE:5259272	-	BF968097	3.17	0.02	2.25	0.01
CDNA clone IMAGE:5278089	-	AW451197	2.3	0.02	3.03	0.01
CDNA clone IMAGE:5284125	-	AI858493	2.61	0.02	2.34	0.01
CDNA clone IMAGE:6254031	-	AI800713	2.47	0.01	2.42	0.01
CDNA FLJ11313 fis, clone PLACE1010106, highly similar to Homo sapiens mRNA; cDNA DKFZp586M1418	-	AL049385	2.61	0.02	2.44	0.01
CDNA FLJ12909 fis, clone NT2RP2004400	-	AU151107	2.48	0.01	2.3	0.01
CDNA FLJ13585 fis, clone PLACE1009150	-	AU157716	5.8	0.01	4.17	0.01
CDNA FLJ14388 fis, clone HEMBA1002716	-	AA147884	12.17	0.01	9.69	0.01
CDNA FLJ20769 fis, clone COL06674	-	AK000776	2.47	0.01	2.05	0.01

CDNA FLJ25556 fis, clone JTH02629	-	AA191741	2.93	0.01	2.52	0.01
CDNA FLJ26539 fis, clone KDN09310	-	AW025579	4.13	0.01	3.09	0.01
CDNA FLJ31814 fis, clone NT2RI2009585	-	AK001164	2.49	0.01	2.16	0.01
CDNA FLJ32438 fis, clone SKMUS2001402	-	AI200555	2.44	0.01	2.21	0.01
CDNA FLJ34100 fis, clone FCBBF3007597	-	BF976693	3.08	0.01	2.61	0.01
CDNA FLJ37310 fis, clone BRAMY2016706	-	AI633559	3.53	0.01	2.51	0.01
CDNA FLJ38396 fis, clone FEBRA2007957	-	R55784	2.27	0.01	2.12	0.01
CDNA: FLJ21027 fis, clone CAE07110	-	AK024680	2.53	0.01	2.19	0.01
Cell adhesion molecule 1	CADM1	AL519710	2.6	0.01	2.56	0.01
Cell cycle progression 1	CCPG1	AK022459	3.19	0.01	2.28	0.01
Cell division cycle 6 homolog (S. cerevisiae)	CDC6	NM_001254	2.86	0.01	2.26	0.01
Centromere protein A	CENPA	NM_001809	2.87	0.01	2.11	0.01
Centromere protein Q	CENPQ	NM_018132	2.63	0.01	2.04	0.01
Centrosomal protein 170kDa	CEP170	NM_014812	2.36	0.03	2.09	0.01
Centrosomal protein 55kDa	CEP55	NM_018131	3.07	0.01	2.49	0.01
ChaC, cation transport regulator homolog 2 (E. coli)	CHAC2	AI191897	3.11	0.01	2.36	0.01
Chemokine (C-C motif) ligand 11	CCL11	D49372	8.09	0.01	5.43	0.01
Chemokine (C-C motif) ligand 18 (pulmonary and activation-regulated)	CCL18	AB000221	3.02	0.02	2.56	0.01
Chemokine (C-C motif) ligand 2	CCL2	S69738	3.16	0.01	2.73	0.01
Chemokine (C-C motif) ligand 3	CCL3	NM_002983	3.66	0.01	2.77	0.01
Chemokine (C-C motif) ligand 4	CCL4	NM_002984	3.94	0.01	2.96	0.01
Chemokine (C-C motif) receptor 1	CCR1	AI421071	3.79	0.01	2.72	0.01
Chemokine (C-C motif) receptor 10	CCR10	NM_016602	2.49	0.01	2.11	0.01
chemokine (C-C motif) receptor 2	-	NM_000647	4.33	0.01	3.39	0.01
Chemokine (C-C motif) receptor 5	CCR5	NM_000579	2.68	0.01	2.15	0.01
Chemokine (C-C motif) receptor 6	CCR6	NM_004367	3.32	0.02	2.56	0.01
Chemokine (C-C motif) receptor-like 1	CCRL1	NM_016557	5.18	0.01	3.13	0.01
Chemokine (C-X-C motif) ligand 1 (melanoma growth stimulating activity, alpha)	CXCL1	NM_001511	18.35	0.01	13.43	0.01
Chemokine (C-X-C motif) ligand 11	CXCL11	AF002985	14.19	0.01	12.26	0.01
Chemokine (C-X-C motif) ligand 2	CXCL2	M57731	10.29	0.01	9.44	0.01
Chemokine (C-X-C motif) ligand	CXCL3	NM_002090	9.44	0.01	8.29	0.01

3						
Chemokine (C-X-C motif) ligand 5	CXCL5	AK026546	44.64	0.01	22.12	0.01
Chemokine (C-X-C motif) ligand 6 (granulocyte chemotactic protein 2)	CXCL6	NM_002993	30.42	0.01	18.36	0.01
Chemokine (C-X-C motif) ligand 9	CXCL9	NM_002416	4.13	0.02	3.95	0.01
Chemokine (C-X-C motif) receptor 6	CXCR6	NM_006564	2.54	0.01	2.04	0.01
Chemokine (C-X-C motif) receptor 7	CXCR7	AI817041	3.38	0.01	2.72	0.01
Chemokine-like factor	CKLF	NM_016951	2.69	0.01	2.1	0.01
chimerin (chimaerin) 1	-	BF339445	2.79	0.01	2.29	0.01
Chitinase 3-like 1 (cartilage glycoprotein-39)	CHI3L1	M80927	33.46	0.01	21.81	0.01
CHK1 checkpoint homolog (S. pombe)	CHEK1	NM_001274	2.34	0.01	2.17	0.01
Chloride channel 4	CLCN4	AF052117	3.38	0.01	2.55	0.01
Chloride intracellular channel 4	CLIC4	AI638420	2.74	0.01	2.18	0.01
Cholesterol 25-hydroxylase	CH25H	NM_003956	3.66	0.01	2.99	0.01
Cholesteryl ester transfer protein, plasma	CETP	NM_000078	2.18	0.01	2.27	0.01
Cholinergic receptor, nicotinic, alpha 5	CHRNA5	NM_000745	2.63	0.01	2.1	0.01
Chondroitin sulfate N-acetylgalactosaminyltransferase 1	CSGALNACT1	NM_018371	3.61	0.01	3.09	0.01
chondroitin sulfate N-acetylgalactosaminyltransferase 2	-	AL139812	2.63	0.01	2.17	0.01
Chondroitin sulfate synthase 1	CHSY1	NM_014918	2.84	0.01	2.27	0.01
Chordin-like 2	CHRDL2	AF332891	2.92	0.01	2.78	0.01
Chromosome 1 open reading frame 38	C1orf38	AB035482	2.38	0.04	2.26	0.01
Chromosome 12 open reading frame 23	C12orf23	AK001731	2.96	0.01	2.54	0.01
Chromosome 12 open reading frame 32	C12orf32	L23518	6.21	0.01	4.7	0.01
Chromosome 12 open reading frame 48	C12orf48	AI224977	2.61	0.01	2.02	0.01
Chromosome 13 open reading frame 15	C13orf15	NM_014059	2.55	0.05	2.27	0.01
Chromosome 13 open reading frame 33	C13orf33	AW084730	2.45	0.01	2.08	0.01
Chromosome 14 open reading frame 145	C14orf145	AI937080	6.22	0.01	3.77	0.01
Chromosome 16 open reading frame 54	C16orf54	BC025741	3.8	0.03	2.9	0.01
Chromosome 19 open reading frame 59	C19orf59	BF433657	2.12	0.02	2.23	0.01
Chromosome 2 open reading frame 30	C2orf30	AK001913	2.48	0.01	2.03	0.01
Chromosome 20 open reading	C20orf103	NM_012261	2.49	0.01	2.12	0.01

frame 103						
Chromosome 21 open reading frame 45	C21orf45	AW151538	2.72	0.01	2.03	0.01
Chromosome 3 open reading frame 34	C3orf34	AW665748	2.32	0.01	2.08	0.01
chromosome 3 open reading frame 64	-	AK023140	2.62	0.01	2.35	0.01
Chromosome 4 open reading frame 32	C4orf32	AI110850	3.15	0.01	2.26	0.01
Chromosome 4 open reading frame 46	C4orf46	BE620598	2.75	0.01	2.03	0.01
Chromosome 6 open reading frame 150	C6orf150	AK097148	5.24	0.01	3.21	0.01
Chromosome 6 open reading frame 173	C6orf173	BG492359	2.77	0.01	2.37	0.01
Chromosome 8 open reading frame 4	C8orf4	NM_020130	2.25	0.01	2.25	0.01
Chromosome 9 open reading frame 3	C9orf3	AL137535	2.43	0.01	2.06	0.01
CKLF-like MARVEL transmembrane domain containing 2	CMTM2	AA778552	2.54	0.01	2.42	0.01
Claudin 1	CLDN1	AF101051	8.57	0.01	4.59	0.01
Claudin 2	CLDN2	AF177340	3.53	0.01	2.79	0.01
Clone 24405 mRNA sequence	-	AA530995	4.5	0.01	3.62	0.01
Clone CPRF1-T2 immunoglobulin lambda chain VJ region, (IGL)	-	AF043586	5.63	0.01	4.35	0.01
Coagulation factor II (thrombin) receptor	F2R	NM_001992	5.12	0.01	3.93	0.01
Coagulation factor II (thrombin) receptor-like 2	F2RL2	AI378647	5.13	0.01	4.09	0.01
Coagulation factor III (thromboplastin, tissue factor)	F3	NM_001993	3.12	0.01	2.54	0.01
Cofilin 2 (muscle)	CFL2	AV726166	2.98	0.01	2.3	0.01
Coiled-coil domain containing 109B	CCDC109 B	NM_017918	2.46	0.01	2.01	0.01
Coiled-coil domain containing 3	CCDC3	AL136562	2.95	0.01	2.37	0.01
Coiled-coil domain containing 69	CCDC69	BC016647	2.58	0.03	2.1	0.01
Collagen triple helix repeat containing 1	CTHRC1	AA584310	10.76	0.01	6.68	0.01
Collagen, type I, alpha 1	COL1A1	AI743621	5.58	0.01	4.09	0.01
Collagen, type I, alpha 2	COL1A2	NM_000089	5.01	0.01	4.08	0.01
collagen, type III, alpha 1	-	AF130082	3.05	0.01	2.4	0.01
Collagen, type IV, alpha 1	COL4A1	NM_001845	4.27	0.01	3.65	0.01
Collagen, type IV, alpha 2	COL4A2	AA909035	2.48	0.01	2.13	0.01
Collagen, type V, alpha 1	COL5A1	AI983428	2.85	0.01	2.52	0.01
Collagen, type V, alpha 2	COL5A2	NM_000393	3.53	0.01	3.08	0.01
Collagen, type VI, alpha 2	COL6A2	AY029208	2.14	0.01	2.02	0.01
Collagen, type VI, alpha 3	COL6A3	NM_004369	4.24	0.01	3.97	0.01
collagen, type XII, alpha 1	-	AL096771	3.95	0.01	2.97	0.01
Collagen, type XIV, alpha 1	COL14A1	BF449063	2.51	0.03	2.34	0.01

Collagen, type XV, alpha 1	COL15A1	NM_001855	3.13	0.01	2.46	0.01
Collagen, type XVIII, alpha 1	COL18A1	AF018081	2.2	0.01	2.15	0.01
Colony stimulating factor 2 receptor, beta, low-affinity (granulocyte-macrophage)	CSF2RB	AV756141	3.63	0.02	2.86	0.01
Complement component (3b/4b) receptor 1-like	CR1L	BE552138	2.36	0.01	2.34	0.01
Complement component 1, r subcomponent	C1R	AL573058	2.57	0.01	2.48	0.01
complement component 1, s subcomponent	-	BC007010	5.01	0.01	3.23	0.01
Complement component 2	C2	NM_000063	2.68	0.01	2.25	0.01
Complement component 4 binding protein, alpha	C4BPA	NM_000715	10.31	0.01	5.94	0.01
Complement factor B	CFB	NM_001710	3.44	0.01	3.19	0.01
Complement factor I	CFI	BC020718	6.36	0.01	3.38	0.01
Connective tissue growth factor	CTGF	M92934	2.56	0.01	2.35	0.01
Consensus includes gb:AI694413 /FEA=EST /DB_XREF=gi:4971753 /DB_XREF=est:wd83d12.x1 /CLONE=IMAGE:2338199 /UG=Hs.332649 olfactory receptor, family 2, subfamily I, member 6	-	AI694413	4.02	0.01	3.52	0.01
Consensus includes gb:T65020 /FEA=EST /DB_XREF=gi:674065 /DB_XREF=est:yc75b10.s1 /CLONE=IMAGE:21583 /UG=Hs.12699 ESTs	-	T65020	2.44	0.01	2.27	0.01
C-type lectin domain family 2, member B	CLEC2B	BC005254	2.97	0.01	2.57	0.01
C-type lectin domain family 4, member A	CLEC4A	AF200738	3.41	0.01	2.63	0.01
C-type lectin domain family 4, member A	CLEC4A	NM_016184	3.92	0.01	3	0.01
C-type lectin domain family 5, member A	CLEC5A	NM_013252	3.2	0.01	2.7	0.01
C-type lectin domain family 7, member A	-	AF400600	3.44	0.01	2.76	0.01
C-type lectin domain family 7, member A	CLEC7A	AF313468	2.79	0.04	2.5	0.01
CUG triplet repeat, RNA binding protein 2	CUGBP2	NM_006561	2.44	0.01	2.14	0.01
Cyclin A2	CCNA2	AI346350	2.67	0.01	2.1	0.01
Cyclin B1	CCNB1	BE407516	2.71	0.01	2.1	0.01
Cyclin B1	CCNB1	N90191	3.06	0.01	2.19	0.01
Cyclin-dependent kinase inhibitor 3	CDKN3	AF213040	2.66	0.01	2.08	0.01
Cystatin A (stefin A)	CSTA	NM_005213	2.76	0.04	2.45	0.01
Cystatin F (leukocystatin)	CST7	AF031824	3.1	0.01	2.46	0.01
Cysteine and glycine-rich protein 2	CSRP2	NM_001321	2.13	0.05	2.23	0.01

Cysteine-rich secretory protein LCCL domain containing 2	CRISPLD 2	AL136861	2.77	0.02	2.15	0.01
Cysteine-rich, angiogenic inducer, 61	CYR61	AF003114	3.69	0.01	3.94	0.01
Cysteinyl leukotriene receptor 1	CYSLTR1	BE549540	2.77	0.01	2.31	0.01
Cytidine deaminase	CDA	NM_001785	2.68	0.03	2.26	0.01
Cytochrome P450, family 1, subfamily B, polypeptide 1	CYP1B1	NM_000104	2.2	0.03	2.04	0.02
Cytochrome P450, family 27, subfamily B, polypeptide 1	CYP27B1	NM_000785	2.35	0.01	2.22	0.01
Cytochrome P450, family 39, subfamily A, polypeptide 1	CYP39A1	NM_016593	2.63	0.01	2.38	0.01
Cytochrome P450, family 4, subfamily X, polypeptide 1	CYP4X1	AA557324	2.95	0.01	2.59	0.01
Cytoplasmic polyadenylation element binding protein 4	CPEB4	BE620832	2.91	0.01	2.34	0.01
Cytotoxic and regulatory T cell molecule	CRTAM	NM_019604	2.86	0.01	2.32	0.01
Damage-regulated autophagy modulator	DRAM	NM_018370	2.95	0.01	2.41	0.01
DDHD domain containing 1	DDHD1	AA029818	2.63	0.01	2.17	0.01
Deafness, autosomal dominant 5	DFNA5	NM_004403	2.24	0.02	2.03	0.01
Decorin	DCN	AF138300	2.46	0.03	2.07	0.01
Dedicator of cytokinesis 4	DOCK4	NM_014705	2.7	0.01	2.17	0.01
Defensin, beta 4	DEFB4	NM_004942	14.05	0.01	10.46	0.01
Degenerative spermatocyte homolog 1, lipid desaturase (Drosophila)	DEGS1	NM_003676	2.81	0.01	2.2	0.01
Der1-like domain family, member 3	DERL3	AI655697	5.5	0.01	3.96	0.01
Dermatan sulfate epimerase	DSE	NM_013352	3.57	0.03	2.71	0.01
Dermatan sulfate epimerase-like	DSEL	AK021539	2.83	0.01	2.01	0.03
Dickkopf homolog 3 (Xenopus laevis)	DKK3	AU148057	3.04	0.01	2.4	0.01
Dihydropyrimidinase-like 3	DPYSL3	W72516	4.13	0.01	3.12	0.01
Dihydropyrimidine dehydrogenase	DPYD	NM_000110	2.77	0.02	2.13	0.01
Discs, large (Drosophila) homolog-associated protein 5	DLGAP5	NM_014750	3.26	0.01	2.43	0.01
DnaJ (Hsp40) homolog, subfamily B, member 9	-	AF115512	4.16	0.01	2.84	0.01
DnaJ (Hsp40) homolog, subfamily C, member 10	DNAJC10	AA651899	3.29	0.01	2.52	0.01
DnaJ (Hsp40) homolog, subfamily C, member 12	DNAJC12	NM_021800	2.83	0.02	2.34	0.01
Doublecortin-like kinase 1	DCLK1	AI129626	2.76	0.01	2.04	0.01
Dpy-19-like 1 (C. elegans)	DPY19L1	BI461155	3.36	0.01	2.65	0.01
Drebrin 1	DBN1	NM_004395	2.73	0.01	2.14	0.01
Dual adaptor of phosphotyrosine and 3-phosphoinositides	DAPP1	AA150186	3.64	0.01	3	0.01

Dual oxidase 2	DUOX2	NM_014080	29.14	0.01	25.35	0.01
Dual oxidase maturation factor 2	DUOXA2	AI821606	14	0.01	13.6	0.01
Dual specificity phosphatase 14	DUSP14	NM_007026	2.46	0.01	2.17	0.01
Dual specificity phosphatase 4	DUSP4	BC002671	2.93	0.01	2.28	0.01
Dystrobrevin, beta	DTNB	AK022277	4.94	0.01	3.43	0.01
E2F transcription factor 7	E2F7	AI341146	2.75	0.01	2.31	0.01
Early growth response 2 (Krox-20 homolog, Drosophila)	EGR2	NM_000399	2.67	0.01	2.51	0.01
Early growth response 3	EGR3	NM_004430	2.45	0.02	2.63	0.01
Echinoderm microtubule associated protein like 1	EML1	AI825937	2.45	0.01	2.25	0.01
Ecotropic viral integration site 2B	EVI2B	BC005926	4.1	0.02	2.83	0.01
Ectonucleoside triphosphate diphosphohydrolase 1	ENTPD1	AI301948	2.17	0.01	2.29	0.01
EGF, latrophilin and seven transmembrane domain containing 1	ELTD1	NM_022159	2.37	0.01	2.09	0.01
EGF-containing fibulin-like extracellular matrix protein 1	EFEMP1	AI826799	4.03	0.01	3.36	0.01
Egf-like module containing, mucin-like, hormone receptor-like 2	EMR2	NM_013447	5.01	0.01	4.2	0.01
EGF-like-domain, multiple 6	EGFL6	NM_015507	9.35	0.01	6.33	0.01
ELK3, ETS-domain protein (SRF accessory protein 2)	ELK3	AW575374	3.11	0.01	2.42	0.01
ELL associated factor 2	EAF2	NM_018456	3.53	0.01	2.82	0.01
Elongation factor, RNA polymerase II, 2	ELL2	AI745624	3.74	0.01	3.05	0.01
ELOVL family member 5, elongation of long chain fatty acids (FEN1/Elo2, SUR4/Elo3-like, yeast)	ELOVL5	AL136939	2.27	0.02	2.02	0.01
Endothelin receptor type A	EDNRA	AU118882	3.3	0.01	2.93	0.01
Endothelin receptor type B	EDNRB	M74921	2.62	0.03	2.05	0.02
Eomesodermin homolog (Xenopus laevis)	EOMES	NM_005442	2.32	0.01	2.18	0.01
EPH receptor A2	EPHA2	NM_004431	2.48	0.01	2.19	0.01
EPH receptor A3	EPHA3	AF213459	2.1	0.05	2.03	0.03
Epithelial cell transforming sequence 2 oncogene	ECT2	NM_018098	3.32	0.01	2.5	0.01
Epithelial membrane protein 3	EMP3	NM_001425	2.91	0.01	2.16	0.01
ERO1-like (S. cerevisiae)	ERO1L	AW268365	5.66	0.01	4.02	0.01
ERO1-like beta (S. cerevisiae)	ERO1LB	NM_019891	2.58	0.01	2.2	0.01
Eukaryotic translation initiation factor 1A, X-linked	EIF1AX	BE542684	2.98	0.01	2.1	0.02
Eukaryotic translation initiation factor 5A	EIF5A	NM_001970	3.44	0.01	2.4	0.02
Eukaryotic translation initiation factor 5A2	EIF5A2	BG500474	3.87	0.01	2.86	0.01
Excision repair cross-complementing rodent repair	ERCC6L	NM_017669	2.35	0.01	2.06	0.01

deficiency, complementation group 6-like						
Exocyst complex component 5	EXOC5	BF509391	2.78	0.01	2.11	0.01
Family with sequence similarity 126, member A	FAM126A	AV734839	2.5	0.01	2.15	0.01
family with sequence similarity 129, member A	-	NM_022083	2.43	0.01	2.06	0.01
Family with sequence similarity 148, member A	FAM148A	BE218239	6.08	0.01	5.45	0.01
Family with sequence similarity 162, member B	FAM162B	AI540210	2.58	0.01	2.29	0.01
Family with sequence similarity 171, member B	FAM171B	AW043602	3.57	0.01	3.02	0.01
Family with sequence similarity 176, member A	FAM176A	AV700753	2.62	0.01	2.23	0.01
Family with sequence similarity 19 (chemokine (C-C motif)-like), member A1	FAM19A1	AI824004	2.47	0.01	3.7	0.01
Family with sequence similarity 20, member B	FAM20B	BG536224	2.6	0.01	2.15	0.01
Family with sequence similarity 26, member F	FAM26F	AV734646	4.2	0.03	3.31	0.01
Family with sequence similarity 33, member A	FAM33A	BE048371	3.24	0.01	2.5	0.01
Family with sequence similarity 40, member B	FAM40B	AB032996	3.07	0.01	2.79	0.01
Family with sequence similarity 46, member C	FAM46C	AL046017	3.09	0.01	2.51	0.01
Family with sequence similarity 49, member A	FAM49A	AA243659	2.89	0.03	2.49	0.01
family with sequence similarity 54, member A	-	AL138828	2.46	0.01	2.01	0.01
family with sequence similarity 55, member C	-	BE883167	2.34	0.01	2.06	0.01
family with sequence similarity 92, member A1	-	BF338870	2.43	0.01	2.01	0.01
Fatty acid desaturase 1	FADS1	AL512760	3.22	0.01	3.08	0.01
F-box protein 16	FBXO16	BF196856	2.94	0.01	2.58	0.01
F-box protein 6	FBXO6	AF129536	3.81	0.01	3.25	0.01
Fc fragment of IgE, high affinity I, receptor for; gamma polypeptide	-	BC020763	5.2	0.01	3.61	0.01
Fc fragment of IgG, high affinity Ia, receptor (CD64)	FCGR1A	X14355	2.88	0.01	2.61	0.01
Fc fragment of IgG, low affinity IIa, receptor (CD32)	FCGR2A	NM_021642	4.15	0.01	3.08	0.01
Fc fragment of IgG, low affinity IIc, receptor for (CD32)	FCGR2C	U90940	2.61	0.01	2.11	0.01
Fc fragment of IgG, low affinity IIIb, receptor (CD16b)	FCGR3B	J04162	8.53	0.01	6.83	0.01
Fc receptor-like 5	FCRL5	AF343662	6.18	0.01	4.6	0.01
Fermitin family homolog 2 (Drosophila)	FERMT2	AW469573	2.97	0.01	2.39	0.01
Fibrillin 1	FBN1	AI264196	3.28	0.01	2.78	0.01

Fibrinogen gamma chain	FGG	AI133452	3.27	0.01	2.91	0.01
Fibroblast activation protein, alpha	FAP	U76833	4.04	0.01	3.82	0.01
Fibroblast growth factor 2 (basic)	FGF2	NM_002006	2.42	0.01	2.03	0.01
Fibroblast growth factor 7 (keratinocyte growth factor)	FGF7	NM_002009	3	0.01	2.86	0.01
Fibroblast growth factor binding protein 1	FGFBP1	NM_005130	2.33	0.01	2.12	0.01
Fibronectin 1	FN1	X02761	2.15	0.02	2.32	0.01
Fibronectin leucine rich transmembrane protein 2	FLRT2	NM_013231	2.46	0.03	2.4	0.01
Fibronectin type III domain containing 1	FNDC1	AI345957	4.1	0.01	2.81	0.01
Fibronectin type III domain containing 3B	FNDC3B	AW058617	3.23	0.01	2.35	0.01
Fibulin 1	FBLN1	NM_001996	2.57	0.01	2.03	0.01
Ficolin (collagen/fibrinogen domain containing) 1	FCN1	NM_002003	2.02	0.01	2.15	0.01
Filamin A interacting protein 1-like	FILIP1L	AF329092	2.92	0.02	2.35	0.01
Fin bud initiation factor homolog (zebrafish)	FIBIN	AI802391	5.69	0.01	4	0.01
FK506 binding protein 11, 19 kDa	FKBP11	NM_016594	3.85	0.01	5.3	0.01
FK506 binding protein 7	FKBP7	AA683602	2.7	0.01	2.01	0.01
Flavin containing monooxygenase 3	FMO3	M83772	3.12	0.01	2.33	0.01
Follistatin-like 1	FSTL1	BC000055	2.62	0.01	2.3	0.01
Forkhead box F1	FOXF1	NM_001451	2.65	0.01	2.09	0.01
Forkhead box Q1	FOXQ1	AI676059	6.51	0.01	3.61	0.01
Formyl peptide receptor 1	FPR1	NM_002029	4.29	0.01	3.56	0.01
Formyl peptide receptor 2	FPR2	M88107	3.56	0.01	3.48	0.01
Formyl peptide receptor 3	FPR3	AW026543	3.75	0.02	2.48	0.02
Four jointed box 1 (Drosophila)	FJX1	NM_014344	3.43	0.01	2.95	0.01
Free fatty acid receptor 2	FFAR2	NM_005306	2.45	0.01	2.36	0.01
Friend leukemia virus integration 1	FLI1	NM_002017	2.84	0.02	2.29	0.01
Frizzled homolog 2 (Drosophila)	FZD2	L37882	2.35	0.01	2.08	0.01
fucosyltransferase 11 (alpha (1,3) fucosyltransferase)	-	BF541967	2.63	0.01	2.09	0.01
Fucosyltransferase 8 (alpha (1,6) fucosyltransferase)	FUT8	AB049740	3.57	0.01	2.6	0.01
Full length insert cDNA clone ZC64C06	-	AK056897	2.66	0.01	2.34	0.01
G protein-coupled receptor 110	GPR110	BG426455	3.64	0.01	2.5	0.01
G protein-coupled receptor 126	-	AL033377	3.52	0.01	2.57	0.01
G protein-coupled receptor 128	GPR128	NM_032787	3.9	0.01	2.15	0.02
G protein-coupled receptor 137B	GPR137B	NM_003272	3.2	0.01	2.39	0.01
G protein-coupled receptor 180	GPR180	AU160685	3.04	0.01	2.4	0.01
G protein-coupled receptor 183	GPR183	NM_004951	3	0.03	2.35	0.01
G protein-coupled receptor 19	GPR19	NM_006143	2.51	0.01	2.1	0.01

G protein-coupled receptor 37 (endothelin receptor type B-like)	-	U87460	2.87	0.01	2.12	0.01
G protein-coupled receptor 65	GPR65	NM_003608	3.13	0.03	2.45	0.01
G protein-coupled receptor 84	GPR84	AF237762	2.6	0.01	2.52	0.01
G protein-coupled receptor 85	GPR85	AL161959	2.09	0.01	2.06	0.01
G0/G1switch 2	G0S2	NM_015714	2.11	0.05	2.1	0.01
Galanin prepropeptide	GAL	AL556409	2.58	0.02	2.85	0.01
Gamma-aminobutyric acid (GABA) A receptor, pi	GABRP	NM_014211	2.6	0.01	2.49	0.01
Gap junction protein, alpha 1, 43kDa	GJA1	NM_000165	4.34	0.01	3.45	0.01
Gap junction protein, alpha 5, 40kDa	GJA5	AI692880	2.56	0.01	2.3	0.01
Gap junction protein, gamma 1, 45kDa	GJC1	AA430014	3.09	0.01	2.8	0.01
Gardner-Rasheed feline sarcoma viral (v-fgr) oncogene homolog	FGR	NM_005248	2.2	0.01	2.19	0.01
General transcription factor IIA, 1-like	GTF2A1L	BG434174	2.62	0.01	2.24	0.01
GLI pathogenesis-related 1	-	AV682252	2.53	0.02	2.17	0.01
glucocorticoid induced transcript 1	-	AC006042	3.6	0.01	2.85	0.01
Glucuronidase, beta/ immunoglobulin lambda-like polypeptide 1 pseudogene	LOC91316	AA398569	2.69	0.01	2.49	0.01
Glutaminyl-peptide cyclotransferase	QPCT	NM_012413	2.89	0.02	2.16	0.02
Glutathione peroxidase 7	GPX7	AA406605	2.52	0.01	2.24	0.01
glutathione peroxidase 8 (putative)	-	AA173223	3.47	0.01	2.6	0.01
Glycine dehydrogenase (decarboxylating)	GLDC	NM_000170	3.62	0.01	2.1	0.01
Glycosyltransferase 8 domain containing 2	GLT8D2	W63754	2.63	0.03	2.23	0.01
Glypican 6	GPC6	AI651255	2.48	0.01	2.02	0.01
Golgi transport 1 homolog B (S. cerevisiae)	GOLT1B	NM_016072	2.8	0.01	2.3	0.01
Grainyhead-like 1 (Drosophila)	GRHL1	BE566136	3.4	0.01	3.49	0.01
granulysin	-	M85276	2.21	0.01	2.16	0.01
Granzyme A (granzyme 1, cytotoxic T-lymphocyte-associated serine esterase 3)	GZMA	NM_006144	2.76	0.01	2.03	0.01
Granzyme B (granzyme 2, cytotoxic T-lymphocyte-associated serine esterase 1)	GZMB	J03189	4.31	0.01	3.05	0.01
Gremlin 1, cysteine knot superfamily, homolog (Xenopus laevis)	GREM1	AF154054	7.3	0.01	6	0.01
Growth arrest-specific 1	GAS1	NM_002048	4.55	0.02	3.26	0.01
GTP binding protein overexpressed in skeletal muscle	GEM	NM_005261	2.45	0.01	2.23	0.01

Guanine nucleotide binding protein (G protein), alpha 14	GNA14	NM_004297	3	0.01	2.26	0.01
Guanine nucleotide binding protein (G protein), alpha 15 (Gq class)	GNA15	NM_002068	3.6	0.01	2.95	0.01
Guanine nucleotide binding protein (G protein), beta polypeptide 4	GNB4	H99792	3.26	0.01	2.41	0.01
Guanylate binding protein 1, interferon-inducible, 67kDa	GBP1	AW014593	2.35	0.02	2.26	0.01
Guanylate binding protein 4	GBP4	BG260886	2.41	0.01	2.02	0.01
Guanylate binding protein 5	GBP5	BG271923	3.61	0.01	2.93	0.01
Guanylate cyclase 1, soluble, alpha 3	GUCY1A3	AI758408	2.9	0.02	2.61	0.01
Guanylate cyclase 1, soluble, beta 3	GUCY1B3	AF020340	2.54	0.01	2.33	0.01
Heat shock protein 70kDa family, member 13	HSPA13	AI718418	4.66	0.01	3.16	0.01
Helicase, lymphoid-specific	HELLS	AF155827	2.64	0.01	2.03	0.01
Hemoglobin, alpha 1	HBA1	AF105974	2.67	0.01	2.38	0.01
hemoglobin, alpha 2	-	V00489	2.43	0.01	2.13	0.01
hemoglobin, beta	-	AF059180	4.91	0.01	4.04	0.01
Hemoglobin, delta	HBD	NM_000519	2.05	0.01	2	0.01
Heparan sulfate (glucosamine) 3-O-sulfotransferase 3B1	HS3ST3B1	AA780067	3.91	0.02	2.87	0.01
heparan sulfate 6-O-sulfotransferase 2	-	NM_147174	2.64	0.01	2.68	0.01
Hepatitis A virus cellular receptor 2	HAVCR2	AW025572	2.83	0.02	2.31	0.01
hepatocyte growth factor (hepapoietin A; scatter factor)	-	X16323	3.79	0.01	3.41	0.01
Hepatoma-derived growth factor, related protein 3	HDGFRP3	AB029156	2.46	0.01	2.13	0.01
Homo sapiens partial IGVH4 gene for immunoglobulin heavy chain V region, case 2, cell D 56	-	AJ275453	3	0.01	2.25	0.01
Homo sapiens, clone IMAGE:3881549, mRNA	-	BE222344	3.83	0.01	3.03	0.01
Homogentisate 1,2-dioxygenase (homogentisate oxidase)	HGD	NM_000187	2.48	0.01	2.15	0.01
HOP homeobox	HOPX	AB059408	3.63	0.01	2.61	0.01
HtrA serine peptidase 1	HTRA1	NM_002775	2.37	0.01	2.03	0.01
Hyaluronan synthase 2	HAS2	NM_005328	2.52	0.01	2.14	0.01
Hydroxysteroid (11-beta) dehydrogenase 1	HSD11B1	NM_005525	4.12	0.01	3.84	0.01
Hydroxysteroid (17-beta) dehydrogenase 6 homolog (mouse)	HSD17B6	U89281	3	0.01	2.32	0.02
Hypothetical gene supported by AK096370	FLJ39051	BF343672	2.53	0.01	2.19	0.01
Hypothetical LOC145788	FLJ27352	AA398658	2.41	0.01	2.11	0.01

Hypothetical LOC645784	FLJ40330	AA632139	3.15	0.04	2.82	0.01
Hypothetical protein LOC100132891	LOC100132891	AI948599	2.33	0.01	2.11	0.01
Hypothetical protein LOC387763	LOC387763	AW276078	2.65	0.02	2.4	0.01
hypothetical protein LOC643977	-	BE875232	2.17	0.01	2.28	0.01
hypothetical protein MGC16075	-	NM_032761	2.09	0.01	2.02	0.02
Hypothetical protein MGC29506	MGC29506	AF151024	7.16	0.01	5.32	0.01
Hypothetical protein MGC29506	MGC29506	NM_016459	3.89	0.01	3.4	0.01
Ig lambda light chain variable region	-	AW405975	5.01	0.03	4.02	0.01
IGL mRNA for immunoglobulin lambda light chain, partial cds, clone: F004-101L	-	AJ388663	4.39	0.01	3.09	0.01
IKK interacting protein	-	BG498328	3.25	0.01	2.51	0.01
Immunoglobulin (mAb56) light chain V region mRNA, partial sequence	-	D84140	7.41	0.01	5.42	0.01
Immunoglobulin anti-HBsAg lambda light chain (LM25)	-	AI952772	3.3	0.01	2.66	0.01
Immunoglobulin heavy chain variable region (clone Tmu25)	-	L06102	2.89	0.01	2.11	0.01
immunoglobulin heavy constant alpha 1 /// immunoglobulin heavy constant delta /// immunoglobulin heavy constant gamma 1 (G1m marker) /// immunoglobulin heavy constant mu /// immunoglobulin heavy variable 3-23 /// immunoglobulin heavy variable 4-31	-	AB035175	9.77	0.01	6.28	0.01
immunoglobulin heavy constant alpha 1 /// immunoglobulin heavy constant gamma 1 (G1m marker) /// immunoglobulin heavy constant gamma 3 (G3m marker) /// immunoglobulin heavy constant mu /// similar to Ig heavy chain V-III region VH26 precursor	-	AJ275408	2.49	0.01	2.08	0.01
immunoglobulin heavy constant alpha 1 /// immunoglobulin heavy constant gamma 1 (G1m marker) /// immunoglobulin heavy variable 3-23	-	AJ275355	3.12	0.01	2.42	0.01
immunoglobulin heavy constant alpha 1 /// immunoglobulin heavy constant gamma 1 (G1m marker) /// similar to hCG1773549	-	AJ275397	4.83	0.01	4	0.01
immunoglobulin heavy constant delta	-	AJ275469	6.37	0.01	4.99	0.01

Immunoglobulin heavy constant gamma 1 (G1m marker)	-	AJ275413	4.06	0.01	3.07	0.01
Immunoglobulin heavy constant gamma 1 (G1m marker)	IGHG1	AJ239383	2.75	0.01	3.91	0.01
Immunoglobulin heavy constant gamma 1 (G1m marker)	IGHG1	BC001872	4.31	0.03	3.68	0.01
Immunoglobulin heavy constant gamma 1 (G1m marker)	IGHG1	BG340548	6.07	0.01	4.04	0.01
Immunoglobulin heavy constant gamma 1 (G1m marker)	IGHG1	L06101	4.04	0.01	2.95	0.01
Immunoglobulin heavy constant gamma 1 (G1m marker)	IGHG1	L23516	8.52	0.01	5.7	0.01
Immunoglobulin heavy constant gamma 1 (G1m marker)	IGHG1	L23519	3.45	0.01	3	0.01
Immunoglobulin heavy constant gamma 1 (G1m marker)	IGHG1	L34164	6.11	0.01	4.35	0.01
Immunoglobulin heavy constant gamma 1 (G1m marker)	IGHG1	M24668	3.71	0.01	2.86	0.01
Immunoglobulin heavy constant gamma 1 (G1m marker)	IGHG1	M24669	7.14	0.01	5.44	0.01
Immunoglobulin heavy constant gamma 1 (G1m marker)	IGHG1	M24670	5.88	0.01	4.43	0.01
Immunoglobulin heavy constant gamma 1 (G1m marker)	IGHG1	M87268	4.17	0.01	3.27	0.01
Immunoglobulin heavy constant gamma 1 (G1m marker)	IGHG1	M87789	5.11	0.01	5.82	0.01
Immunoglobulin heavy constant gamma 1 (G1m marker)	IGHG1	S74639	3.01	0.01	2.37	0.01
immunoglobulin heavy constant mu	-	U80139	9.47	0.01	6.25	0.01
immunoglobulin heavy constant mu /// similar to Ig heavy chain V-III region VH26 precursor	-	BF002659	2.92	0.01	2.55	0.01
immunoglobulin heavy locus /// immunoglobulin heavy constant alpha 1 /// immunoglobulin heavy constant alpha 2 (A2m marker) /// immunoglobulin heavy constant delta /// immunoglobulin heavy constant gamma 1 (G1m marker) /// immunoglobulin heavy consta	-	AJ225093	2.84	0.01	2.25	0.01
immunoglobulin heavy locus /// immunoglobulin heavy constant alpha 1 /// immunoglobulin heavy constant gamma 1 (G1m marker) /// immunoglobulin heavy constant gamma 3 (G3m marker) /// immunoglobulin heavy constant mu /// immunoglobulin heavy variable	-	AJ225092	4.53	0.01	3.55	0.01
immunoglobulin heavy locus /// immunoglobulin heavy constant alpha 1 /// immunoglobulin heavy constant gamma 1 (G1m	-	U92706	5	0.01	3.76	0.01

marker) /// immunoglobulin heavy constant gamma 3 (G3m marker) /// immunoglobulin heavy constant mu /// immunoglobulin heavy variable						
Immunoglobulin kappa chain, V-region (SPK.3)	-	AF103530	5.58	0.01	4	0.01
Immunoglobulin kappa constant	IGKC	BG485135	2.37	0.01	2.02	0.01
Immunoglobulin kappa constant	IGKC	BG548679	4.56	0.01	3.73	0.01
Immunoglobulin kappa constant	IGKC	L14457	8.94	0.01	5.6	0.01
Immunoglobulin kappa constant	IGKC	L14458	7.49	0.01	4.91	0.01
Immunoglobulin kappa light chain (IGKV) mRNA variable region, joining region, and constant region	-	M85256	10.62	0.01	6.65	0.01
immunoglobulin kappa variable 1/OR15-118 pseudogene	-	M20812	5.06	0.01	3.7	0.01
immunoglobulin kappa variable 1/OR2-108 (non-functional)	-	X51887	6.26	0.01	4.35	0.01
Immunoglobulin kappa variable 1D-13	IGKV1D-13	AW408194	6.96	0.01	4.87	0.01
immunoglobulin kappa variable 1D-8	-	Z00008	5.15	0.01	3.08	0.01
Immunoglobulin lambda locus	-	AJ275399	2.63	0.01	2.07	0.01
Immunoglobulin lambda locus	-	D87016	2.49	0.04	2.37	0.01
immunoglobulin lambda locus	-	D87021	8.66	0.01	5.53	0.01
Immunoglobulin lambda locus	-	L21961	7.48	0.01	4.98	0.01
immunoglobulin lambda locus	-	X84340	2.54	0.01	2.4	0.01
Immunoglobulin lambda locus	IGL@	AA680302	2.63	0.01	2.19	0.01
Immunoglobulin lambda locus	IGL@	AB001733	6.76	0.01	5.08	0.01
Immunoglobulin lambda locus	IGL@	AB014341	4.87	0.01	4.12	0.01
Immunoglobulin lambda locus	IGL@	AF103591	6.47	0.01	4.63	0.01
Immunoglobulin lambda locus	IGL@	AF234255	7.1	0.01	5.54	0.01
Immunoglobulin lambda locus	IGL@	AJ249377	2.77	0.01	6.51	0.01
Immunoglobulin lambda locus	IGL@	AV698647	2.34	0.01	2.03	0.01
Immunoglobulin lambda locus	IGL@	D01059	4.75	0.01	3.79	0.01
Immunoglobulin lambda locus	IGL@	D84143	6.11	0.01	4.76	0.01
Immunoglobulin lambda locus	IGL@	H53689	11.39	0.01	7.87	0.01
Immunoglobulin lambda locus	IGL@	M87790	3.29	0.01	2.77	0.01
Immunoglobulin lambda locus	IGL@	X57812	2.53	0.01	2.16	0.01
Immunoglobulin lambda locus	IGL@	X93006	7.95	0.01	6.15	0.01
immunoglobulin lambda locus /// immunoglobulin lambda variable 3-19	-	AF047245	3.67	0.01	3.26	0.01
Immunoglobulin lambda variable 1-44	IGLV1-44	U96394	15.57	0.01	9.51	0.01
immunoglobulin lambda-like polypeptide 3	-	AL022324	2.48	0.01	2.24	0.01
Immunoglobulin superfamily, DCC subclass, member 4	IGDCC4	AB046848	4.05	0.01	3.33	0.01
Immunoglobulin superfamily, member 6	IGSF6	NM_005849	2.85	0.02	2.43	0.01

Indoleamine 2,3-dioxygenase 1	IDO1	M34455	4.23	0.01	4.17	0.01
Inducible T-cell co-stimulator	ICOS	AB023135	2.34	0.03	2.23	0.01
Inhibin, beta A	INHBA	M13436	5.41	0.01	4.66	0.01
Inositol monophosphatase domain containing 1	IMPAD1	AI302253	2.96	0.01	2.15	0.01
Inscuteable homolog (Drosophila)	INSC	BF432206	2.62	0.03	2.51	0.01
Insulin induced gene 1	INSIG1	NM_005542	2.25	0.01	2.02	0.01
Insulin receptor substrate 1	IRS1	NM_005544	2.64	0.01	2.52	0.01
Insulin-like growth factor 2 mRNA binding protein 3	IGF2BP3	NM_006547	3.75	0.01	3.39	0.01
Insulin-like growth factor binding protein 5	IGFBP5	AW007532	4.18	0.01	3.77	0.01
Insulin-like growth factor binding protein 7	IGFBP7	NM_001553	2.26	0.01	2.02	0.01
integral membrane protein 2A	-	AL021786	3.35	0.01	2.21	0.01
Integrin, alpha 2 (CD49B, alpha 2 subunit of VLA-2 receptor)	ITGA2	N95414	3.1	0.01	2.57	0.01
Integrin, alpha 4 (antigen CD49D, alpha 4 subunit of VLA-4 receptor)	ITGA4	L12002	3.61	0.01	2.56	0.01
Intercellular adhesion molecule 1	ICAM1	AI608725	2.4	0.01	2.21	0.01
Interferon (alpha, beta and omega) receptor 2	IFNAR2	NM_000874	2.79	0.01	2.29	0.01
Interferon regulatory factor 4	IRF4	NM_002460	4.16	0.01	3.3	0.01
interferon, gamma	-	M29383	3.81	0.01	3.32	0.01
Interferon, gamma-inducible protein 30	IFI30	NM_006332	2.27	0.02	2.07	0.01
Interferon-induced protein with tetratricopeptide repeats 3	IFIT3	AI075407	3.48	0.01	2.7	0.01
interleukin 1 receptor antagonist	-	U65590	5.54	0.01	5.42	0.01
Interleukin 1 receptor-like 1	IL1RL1	NM_003856	2.75	0.01	2.2	0.01
Interleukin 1, alpha	IL1A	M15329	4.94	0.01	3.81	0.01
Interleukin 1, beta	IL1B	M15330	5.38	0.01	4.78	0.01
Interleukin 10 receptor, alpha	IL10RA	NM_001558	2.41	0.04	2.01	0.01
Interleukin 11	IL11	NM_000641	2.84	0.01	2.6	0.01
Interleukin 13 receptor, alpha 2	IL13RA2	NM_000640	12.33	0.01	9.62	0.01
Interleukin 17A	IL17A	Z58820	4.76	0.01	4.86	0.01
Interleukin 2 receptor, alpha	IL2RA	K03122	2.56	0.01	2.3	0.01
Interleukin 21 receptor	IL21R	AF269133	3.21	0.01	2.69	0.01
Interleukin 24	IL24	NM_006850	3.49	0.02	2.45	0.01
Interleukin 26	IL26	NM_018402	2.19	0.01	2.37	0.01
Interleukin 33	IL33	AB024518	3.05	0.03	2.85	0.01
Interleukin 6 (interferon, beta 2)	IL6	NM_000600	4.55	0.01	3.49	0.01
interleukin 8	-	AF043337	8.59	0.01	8.86	0.01
Interleukin 8 receptor, beta	IL8RB	NM_001557	5.33	0.01	5.49	0.01
Interleukin-1 receptor-associated kinase 2	IRAK2	AI246590	2.45	0.01	2.22	0.01
Interleukin-1 receptor-associated kinase 3	IRAK3	AL049435	4.04	0.01	3.4	0.01
IQ motif containing G	-	NM_025111	2.71	0.01	2.22	0.01

Isolate 09 immunoglobulin light chain variable region	-	BG482805	9.31	0.01	5.72	0.01
Isovaleryl Coenzyme A dehydrogenase	IVD	AF043583	11.48	0.01	7.58	0.01
Janus kinase 2 (a protein tyrosine kinase)	JAK2	AF001362	2.64	0.01	2.12	0.01
Jub, ajuba homolog (Xenopus laevis)	JUB	AI289311	4.87	0.01	3.62	0.01
Junctophilin 1	JPH1	AI202201	2.52	0.01	2.33	0.01
Kallikrein-related peptidase 10	KLK10	BC002710	4.9	0.02	3.47	0.01
Kallmann syndrome 1 sequence	KAL1	NM_000216	2.33	0.02	2.17	0.01
KDEL (Lys-Asp-Glu-Leu) containing 1	KDELC1	NM_024089	2.55	0.01	2.12	0.01
KDEL (Lys-Asp-Glu-Leu) endoplasmic reticulum protein retention receptor 3	KDELR3	NM_006855	5.09	0.01	4.13	0.01
Kelch-like 5 (Drosophila)	KLHL5	AK002174	2.95	0.01	2.42	0.01
keratin 6A	-	J00269	4.31	0.02	2.79	0.02
Keratin 6B	KRT6B	AI831452	9.39	0.01	6.52	0.01
Keratin 7	KRT7	BC002700	2.7	0.02	2.1	0.01
Keratinocyte growth factor-like protein 1	KGFLP1	AF523265	3.85	0.01	3.45	0.01
KIAA0125	-	NM_014792	3.02	0.02	2.87	0.01
KIAA0802	KIAA0802	AB018345	2.79	0.01	2.82	0.01
KIAA1199	KIAA1199	AB033025	10.85	0.01	8.5	0.01
KIAA1462	KIAA1462	AL553774	2.01	0.01	2	0.01
Kinesin family member 14	KIF14	AW183154	2.73	0.01	2.26	0.01
Kinesin family member 18A	KIF18A	NM_031217	3.1	0.01	2.17	0.01
Kynureninase (L-kynurenine hydrolase)	KYNU	BC000879	3.82	0.01	3.4	0.01
Kynurenine 3-monooxygenase (kynurenine 3-hydroxylase)	KMO	AI074145	2.93	0.01	2.57	0.01
La ribonucleoprotein domain family, member 6	LARP6	BF792126	2.98	0.01	2.57	0.01
Laminin, gamma 1 (formerly LAMB2)	LAMC1	J03202	2.41	0.01	2.17	0.01
Laminin, gamma 2	LAMC2	NM_005562	2.42	0.01	2.17	0.01
Latent transforming growth factor beta binding protein 2	LTBP2	NM_000428	2.29	0.01	2.06	0.01
Latrophilin 2	LPHN2	NM_012302	3.41	0.01	3.3	0.01
Latrophilin 3	LPHN3	BF511741	2.65	0.04	2.07	0.03
LATS, large tumor suppressor, homolog 2 (Drosophila)	LATS2	AI535735	2.16	0.01	2.02	0.01
Layilin	LAYN	BE856341	2.57	0.01	2.18	0.01
Lectin, galactoside-binding, soluble, 1	LGALS1	NM_002305	2.34	0.01	2.14	0.01
Lectin, mannose-binding, 1	LMAN1	NM_005570	2.95	0.01	2.22	0.01
Leptin receptor	LEPR	U50748	2.85	0.01	2.34	0.01
Leucine rich repeat containing 8 family, member C	LRRC8C	AL136919	2.27	0.03	2.06	0.01
Leukocyte immunoglobulin-like receptor, subfamily B (with TM and ITIM domains), member 2	LILRB2	AF004231	4.05	0.01	3.82	0.01

Leukocyte-associated immunoglobulin-like receptor 2	LAIR2	NM_002288	3.17	0.01	2.86	0.01
LIM and cysteine-rich domains 1	LMCD1	NM_014583	2.3	0.01	2.04	0.01
LIM and senescent cell antigen-like domains 1	LIMS1	AL110164	2.32	0.01	2	0.01
Lipase, endothelial	LIPG	NM_006033	2.51	0.01	2.43	0.01
Lipocalin 2	LCN2	NM_005564	7.12	0.01	6.18	0.01
Lipoma HMGIC fusion partner	LHFP	NM_005780	3.74	0.01	2.87	0.01
Lipoprotein lipase	LPL	BF672975	6.44	0.01	4.22	0.01
Low density lipoprotein receptor-related protein 8, apolipoprotein e receptor	LRP8	NM_004631	2.77	0.01	3.28	0.01
LSM5 homolog, U6 small nuclear RNA associated (S. cerevisiae)	LSM5	NM_012322	3.29	0.01	2.16	0.01
Lumican	LUM	NM_002345	5.42	0.01	3.61	0.01
LY6/PLAUR domain containing 1	LYPD1	AL567376	2.91	0.01	2.3	0.01
LY6/PLAUR domain containing 6B	LYPD6B	BF060747	2.31	0.01	2.01	0.01
Lymphocyte antigen 96	LY96	NM_015364	5.59	0.01	4.06	0.01
Lymphocyte cytosolic protein 2 (SH2 domain containing leukocyte protein of 76kDa)	LCP2	AI123251	3.32	0.01	2.55	0.01
Lymphocyte transmembrane adaptor 1	LAX1	NM_017773	4.7	0.01	4.07	0.01
Lymphocyte-activation gene 3	LAG3	NM_002286	2.41	0.01	2.06	0.01
Lysophosphatidylcholine acyltransferase 1	LPCAT1	NM_024830	2.59	0.01	2.48	0.01
Lysophosphatidylglycerol acyltransferase 1	LPGAT1	NM_014873	2.28	0.01	2.01	0.01
Lysyl oxidase	LOX	NM_002317	3.08	0.01	2.48	0.01
Lysyl oxidase-like 1	LOXL1	NM_005576	3.24	0.01	2.63	0.01
Lysyl oxidase-like 2	LOXL2	NM_002318	4.77	0.01	3.78	0.01
MAD2 mitotic arrest deficient-like 1 (yeast)	MAD2L1	NM_002358	3.53	0.01	2.55	0.01
major histocompatibility complex, class II, DM alpha	-	X76775	2.68	0.01	2.3	0.01
Major histocompatibility complex, class II, DR alpha	HLA-DRA	M60333	2.42	0.03	2.03	0.01
major histocompatibility complex, class II, DR beta 1 /// major histocompatibility complex, class II, DR beta 3 /// major histocompatibility complex, class II, DR beta 4 /// major histocompatibility complex, class II, DR beta 5 /// similar to Major h	-	AA807056	6.33	0.01	3.07	0.02
Major histocompatibility complex, class I-related	MR1	NM_001531	2.95	0.01	2.31	0.01
malic enzyme 1, NADP(+)-dependent, cytosolic	-	AL049699	6.5	0.01	4.44	0.01

Mannosidase, endo-alpha	MANEA	AI587307	3.89	0.01	2.63	0.01
MAS-related GPR, member F	-	H15920	3.77	0.01	3.1	0.01
Matrix Gla protein	MGP	NM_000900	3.09	0.02	2.84	0.01
Matrix metallopeptidase 1 (interstitial collagenase)	MMP1	NM_002421	41.57	0.01	22.03	0.01
Matrix metallopeptidase 10 (stromelysin 2)	MMP10	NM_002425	44.39	0.01	34.11	0.01
Matrix metallopeptidase 12 (macrophage elastase)	MMP12	NM_002426	10.59	0.01	6.65	0.01
Matrix metallopeptidase 19	MMP19	U38321	2.43	0.01	2.19	0.01
Matrix metallopeptidase 2 (gelatinase A, 72kDa gelatinase, 72kDa type IV collagenase)	MMP2	NM_004530	3.74	0.01	2.65	0.01
Matrix metallopeptidase 3 (stromelysin 1, progelatinase)	MMP3	NM_002422	66.73	0.01	49.68	0.01
Matrix metallopeptidase 7 (matrilysin, uterine)	MMP7	NM_002423	9.39	0.01	11.8	0.01
Matrix metallopeptidase 9 (gelatinase B, 92kDa gelatinase, 92kDa type IV collagenase)	MMP9	NM_004994	3.49	0.03	3.3	0.01
Matrix-remodelling associated 5	MXRA5	AF245505	2.89	0.01	2.59	0.01
Mdm2 p53 binding protein homolog (mouse)	MDM2	AI952357	2.79	0.01	2.09	0.01
Melanoma cell adhesion molecule	MCAM	AF089868	2.15	0.01	2.01	0.01
Membrane metallo-endopeptidase	MME	AI433463	4.41	0.01	3.58	0.01
Membrane-associated ring finger (C3HC4) 1	1-Mar	NM_017923	2.93	0.01	2.31	0.01
Membrane-spanning 4-domains, subfamily A, member 2 (Fc fragment of IgE, high affinity I, receptor for; beta polypeptide)	MS4A2	NM_000139	3.73	0.01	2.96	0.01
Mesenchyme homeobox 1	MEOX1	NM_004527	3.08	0.01	3.01	0.01
Methionine sulfoxide reductase B3	MSRB3	AW027333	2.6	0.01	2.19	0.01
methylenetetrahydrofolate dehydrogenase (NADP+ dependent) 1-like	-	AL133260	2.45	0.01	2.27	0.01
Methylenetetrahydrofolate dehydrogenase (NADP+ dependent) 2, methenyltetrahydrofolate cyclohydrolase	MTHFD2	NM_006636	3.01	0.01	2.45	0.01
MHC class I polypeptide-related sequence A	MICA	NM_005931	2.98	0.01	2.63	0.01
Microfibrillar associated protein 5	MFAP5	U37283	2.29	0.01	2.96	0.01
Microphthalmia-associated transcription factor	MITF	AL117653	2.79	0.01	2.22	0.01
Microtubule-associated protein	MAP9	AI887898	3.17	0.01	2.19	0.01

9						
Minichromosome maintenance complex component 10	MCM10	NM_018518	2.77	0.01	2.18	0.01
Mitogen-activated protein kinase kinase kinase 8	MAP3K8	NM_005204	2.19	0.01	2.08	0.01
MORC family CW-type zinc finger 4	MORC4	NM_024657	2.44	0.01	2.14	0.01
mRNA turnover 4 homolog (S. cerevisiae)	-	BG107419	3.14	0.01	2.06	0.01
MRNA; cDNA DKFZp313C0240 (from clone DKFZp313C0240)	-	H16409	3.7	0.01	3.23	0.01
MRNA; cDNA DKFZp564H1663 (from clone DKFZp564H1663)	-	AL117598	2.36	0.01	2.09	0.01
MRNA; cDNA DKFZp586C1322 (from clone DKFZp586C1322)	-	AL049452	2.13	0.01	2.02	0.01
MRNA; cDNA DKFZp586F2224 (from clone DKFZp586F2224)	-	AI655015	2.68	0.01	2.25	0.01
Multimerin 1	MMRN1	NM_007351	4.35	0.01	4.15	0.01
Multiple coagulation factor deficiency 2	MCFD2	AL567779	2.97	0.01	2.33	0.01
mutated in colorectal cancers	-	BE967311	2.56	0.03	2.36	0.01
Myeloid cell nuclear differentiation antigen	MNDA	NM_002432	6.79	0.01	4.77	0.01
MyoD family inhibitor domain containing	MDFIC	BC040713	2.78	0.01	2.42	0.01
Myoferlin	MYOF	AF207990	2.2	0.01	2.03	0.01
Myosin, heavy chain 10, non-muscle	MYH10	AK026977	2.53	0.01	2.07	0.01
Na+/H+ exchanger domain containing 2	NHEDC2	BF433180	2.6	0.01	2.09	0.01
NAD(P)H dehydrogenase, quinone 2	NQO2	NM_000904	2.64	0.01	2.3	0.01
NADPH oxidase 4	NOX4	NM_016931	2.13	0.01	2.15	0.01
Natural killer cell group 7 sequence	NKG7	NM_005601	3.08	0.01	2.24	0.01
Nebulette	NEBL	NM_006393	3.94	0.01	3.24	0.01
Netrin 3	NTN3	AF103529	9.44	0.01	6.1	0.01
Neuregulin 1	NRG1	NM_013959	3.51	0.01	3.14	0.01
Neuromedin U	NMU	NM_006681	2.22	0.01	2.02	0.01
Neuronal cell adhesion molecule	NRCAM	NM_005010	2.76	0.01	2.42	0.01
Neuronal growth regulator 1	NEGR1	AI123532	2.62	0.01	2.02	0.02
Neuronal pentraxin II	NPTX2	U26662	9.51	0.01	4.54	0.01
Neutrophil cytosolic factor 2	NCF2	BC001606	4.1	0.01	3.18	0.01
Nexilin (F actin binding protein)	NEXN	AF114264	2.82	0.01	2.3	0.01
Niacin receptor 2	NIACR2	NM_006018	7.94	0.01	8.03	0.01
Nicotinamide N-methyltransferase	NNMT	NM_006169	2.92	0.01	2.9	0.01
nicotinamide phosphoribosyltransferase	-	BC020691	2.94	0.01	2.32	0.01
Nidogen 1	NID1	BF940043	5	0.01	3.61	0.01
Nidogen 2 (osteonidogen)	NID2	NM_007361	2.44	0.01	2.36	0.01
NIMA (never in mitosis gene a)-	NEK2	NM_002497	2.85	0.01	2.18	0.01

related kinase 2						
Nitric oxide synthase 2, inducible	NOS2	L24553	4.07	0.01	3.54	0.01
non-protein coding RNA 94	-	BC002791	2.7	0.01	2.14	0.01
Nuclear factor of kappa light polypeptide gene enhancer in B-cells inhibitor, zeta	NFKBIZ	AB037925	2.1	0.01	2.13	0.01
Nuclear receptor subfamily 2, group F, member 1	NR2F1	AI951185	2.72	0.02	2.57	0.01
Nuclear receptor subfamily 4, group A, member 2	NR4A2	NM_006186	2.84	0.01	3.13	0.01
Nuclear receptor subfamily 4, group A, member 2	NR4A2	S77154	2.89	0.01	3.25	0.01
Nucleobindin 2	NUCB2	NM_005013	3.41	0.01	2.59	0.01
Nucleosome assembly protein 1-like 3	NAP1L3	NM_004538	2.6	0.01	2.13	0.01
Nucleosome assembly protein 1-like 5	NAP1L5	AW025330	2.86	0.01	2.62	0.01
Nucleotide-binding oligomerization domain containing 2	NOD2	NM_022162	3.19	0.01	2.79	0.01
Olfactomedin 1	OLFM1	NM_006334	2.2	0.01	2.23	0.01
Olfactomedin-like 2B	OLFML2B	AW007573	2.68	0.01	2.78	0.01
Olfactomedin-like 3	OLFML3	NM_020190	2.4	0.01	2.06	0.01
Oncostatin M receptor	OSMR	BC010943	2.59	0.01	2.16	0.01
ORAI calcium release-activated calcium modulator 2	ORAI2	AW205664	2.48	0.01	2.24	0.01
Oxysterol binding protein-like 8	OSBPL8	AL049923	2.87	0.01	2.06	0.01
Paired related homeobox 1	PRRX1	AA775472	4.68	0.01	4.33	0.01
Parvin, beta	PARVB	AA187563	2.57	0.01	2.33	0.01
PDZ and LIM domain 4	PDLIM4	AF153882	2.2	0.01	2.05	0.01
PDZ binding kinase	PBK	NM_018492	3.16	0.01	2.36	0.01
PDZ domain containing 2	-	AF338650	2.47	0.01	2.03	0.01
PDZK1 interacting protein 1	PDZK1IP1	NM_005764	4.88	0.01	3.72	0.01
Peptidase domain containing associated with muscle regeneration 1	PAMR1	AI671186	2.27	0.01	2.04	0.01
Peptidase inhibitor 15	PI15	AI088609	4.49	0.01	3.14	0.01
peptidase inhibitor 3, skin-derived	-	L10343	12.81	0.01	10.77	0.01
Peptidylprolyl isomerase (cyclophilin)-like 1	PPIL1	BC003048	2.35	0.01	2.08	0.01
Peroxidasin homolog (Drosophila)	PXDN	BF342851	2.49	0.02	2.27	0.01
Peroxiredoxin 4	PRDX4	NM_006406	3.61	0.01	2.84	0.01
PFTAIRE protein kinase 1	PFTK1	NM_012395	2.31	0.01	2.04	0.01
PHD finger protein 19	PHF19	BE544837	2.55	0.01	2.28	0.01
Phorbol-12-myristate-13-acetate-induced protein 1	PMAIP1	AI857639	2.48	0.01	2.1	0.01
Phosphatidylinositol glycan anchor biosynthesis, class F	PIGF	BE897886	3.27	0.01	2.65	0.01
Phosphatidylinositol transfer protein, cytoplasmic 1	PITPNC1	AA815089	3.23	0.01	2.6	0.01

Phosphodiesterase 10A	PDE10A	AI143879	2.57	0.01	2.28	0.01
Phosphodiesterase 4B, cAMP-specific (phosphodiesterase E4 dunce homolog, Drosophila)	PDE4B	L20966	3.2	0.01	2.57	0.01
Phosphoglucomutase 3	PGM3	BC001258	2.37	0.01	2.06	0.01
phosphoinositide-3-kinase, regulatory subunit 3 (gamma)	-	BE622627	2.55	0.01	2.11	0.01
Phospholamban	PLN	AI969945	3.59	0.01	3.03	0.01
Phospholipase A1 member A	PLA1A	NM_015900	3.97	0.01	3.27	0.01
phospholipase C, beta 1 (phosphoinositide-specific)	-	AL049593	3.44	0.01	2.71	0.01
Phospholipase C-like 1	PLCL1	NM_006226	2.93	0.01	2.42	0.01
Phosphoribosyl pyrophosphate synthetase 1	PRPS1	NM_002764	2.62	0.01	2.11	0.01
Phosphoserine aminotransferase 1	PSAT1	BC004863	9.33	0.01	5.5	0.01
Phosphoserine phosphatase	PSPH	NM_004577	2.45	0.01	2.01	0.01
Pim-2 oncogene	PIM2	NM_006875	3.69	0.01	3.03	0.01
Plasminogen activator, urokinase	PLAU	K03226	5.79	0.01	4.07	0.01
Plasminogen activator, urokinase receptor	PLAUR	U08839	2.79	0.01	2.11	0.01
Plastin 3 (T isoform)	PLS3	NM_005032	3.12	0.01	2.44	0.01
Platelet factor 4	PF4	NM_002619	3.34	0.01	3.07	0.01
platelet/endothelial cell adhesion molecule	-	AW574504	2.23	0.01	2.32	0.01
Pleckstrin	PLEK	NM_002664	2.23	0.03	2.03	0.01
Pleckstrin homology-like domain, family A, member 1	PHLDA1	AA576961	9.53	0.01	6.48	0.01
Pleckstrin homology-like domain, family B, member 2	PHLDB2	AK025444	2.34	0.01	2.08	0.01
Plexin C1	PLXNC1	AF035307	2.45	0.04	2.19	0.01
podoplanin	-	AU154455	6.93	0.01	5.09	0.01
Polycystic kidney and hepatic disease 1 (autosomal recessive)-like 1	PKHD1L1	AV706971	2.99	0.01	2.37	0.01
Potassium channel tetramerisation domain containing 14	KCTD14	AI672101	3.17	0.01	2.45	0.01
Potassium intermediate/small conductance calcium-activated channel, subfamily N, member 3	KCNN3	NM_002249	2.96	0.01	2.58	0.01
Potassium inwardly-rectifying channel, subfamily J, member 15	KCNJ15	U73191	3.85	0.01	3.59	0.01
Potassium inwardly-rectifying channel, subfamily J, member 8	KCNJ8	BF514158	3	0.01	2.75	0.01
Potassium voltage-gated channel, Isk-related family, member 3	KCNE3	AI692703	2.5	0.01	2.2	0.01
POU class 2 associating factor 1	POU2AF1	NM_006235	3.93	0.04	2.99	0.01

PR domain containing 1, with ZNF domain	PRDM1	AI692659	3.4	0.01	2.55	0.01
Pregnancy-associated plasma protein A, pappalysin 1	PAPPA	AI110886	2.81	0.01	2.56	0.01
Prepronociceptin	PNOC	NM_006228	3.04	0.01	2.37	0.01
Probable ubiquitin-conjugating enzyme E2 FLJ25076	FLJ25076	H17038	3.64	0.01	2.53	0.01
Procollagen C-endopeptidase enhancer	PCOLCE	NM_002593	2.95	0.01	2.72	0.01
Prokineticin 2	PROK2	AF182069	8.71	0.01	8.59	0.01
Proline rich 16	PRR16	NM_016644	5.98	0.01	4.37	0.01
Pro-platelet basic protein (chemokine (C-X-C motif) ligand 7)	PPBP	R64130	2.99	0.01	2.9	0.01
Proprotein convertase subtilisin/kexin type 1	PCSK1	NM_000439	11.66	0.01	8.33	0.01
Proprotein convertase subtilisin/kexin type 9	PCSK9	W92036	2.24	0.01	2.45	0.01
prostaglandin D2 synthase 21kDa (brain)	-	M61900	2.76	0.03	2.4	0.01
prostaglandin F receptor (FP)	-	BC035694	2.5	0.01	2.58	0.01
Prostaglandin-endoperoxide synthase 2 (prostaglandin G/H synthase and cyclooxygenase)	PTGS2	AY151286	5.08	0.01	4.71	0.01
prostate transmembrane protein, androgen induced 1	-	AL035541	2.31	0.01	2.85	0.01
Proteasome (prosome, macropain) subunit, beta type, 9 (large multifunctional peptidase 2)	PSMB9	NM_002800	2.33	0.01	2.06	0.01
Protein C receptor, endothelial (EPCR)	PROCR	NM_006404	3.44	0.01	2.43	0.01
Protein kinase C, delta binding protein	PRKCDBP	AI088622	2.63	0.01	2.51	0.01
Protein kinase D1	PRKD1	NM_002742	2.61	0.01	2.25	0.01
Protein kinase, cAMP-dependent, regulatory, type II, beta	PRKAR2B	NM_002736	3.5	0.01	2.74	0.01
Protein phosphatase 1, regulatory (inhibitor) subunit 16B	PPP1R16B	AB020630	2.16	0.04	2.04	0.01
Protein phosphatase 2C, magnesium-dependent, catalytic subunit	PPM2C	BG542521	3.24	0.01	2.42	0.01
Protein tyrosine phosphatase, non-receptor type 13 (APO-1/CD95 (Fas)-associated phosphatase)	PTPN13	NM_006264	2.64	0.01	2.28	0.01
Protein tyrosine phosphatase, receptor type, G	PTPRG	AI857788	3.74	0.01	2.48	0.01
Protein tyrosine phosphatase-like A domain containing 2	PTPLAD2	AI804932	2.47	0.04	2.01	0.01
protocadherin 17	-	NM_014459	2.05	0.03	2.16	0.01
Protocadherin 18	PCDH18	AW189885	3.75	0.02	2.65	0.01

Protocadherin 7	PCDH7	NM_002589	7.21	0.01	2.07	0.01
Protoporphyrinogen oxidase	PPOX	AW615133	2.35	0.01	2.18	0.01
purinergic receptor P2Y, G-protein coupled, 13	-	NM_023914	3.18	0.02	2.59	0.01
Putative homeodomain transcription factor 1	PHTF1	AA927671	2.84	0.01	2.37	0.01
Pyrin and HIN domain family, member 1	PYHIN1	AI827431	2.31	0.01	2.06	0.01
Pyruvate dehydrogenase kinase, isozyme 1	PDK1	AU146532	2.35	0.01	2.12	0.01
Quaking homolog, KH domain RNA binding (mouse)	QKI	AA149639	3.48	0.01	2.62	0.01
RAB23, member RAS oncogene family	RAB23	AF161486	2.92	0.01	2.28	0.01
RAB31, member RAS oncogene family	RAB31	AF183421	3.04	0.01	2.44	0.01
RAB33A, member RAS oncogene family	RAB33A	NM_004794	2.6	0.01	2.09	0.01
RAB34, member RAS oncogene family	RAB34	AF327350	2.35	0.01	2.12	0.01
RAB38, member RAS oncogene family	RAB38	NM_022337	3.48	0.01	2.66	0.01
RAB39B, member RAS oncogene family	RAB39B	AV724323	2.38	0.01	2.13	0.01
RAB8B, member RAS oncogene family	RAB8B	AB038995	3.61	0.02	2.59	0.01
Ras homolog gene family, member J	RHOJ	AI583530	2.53	0.01	2.19	0.01
Ras homolog gene family, member Q	RHOQ	AW771590	4.3	0.01	3.17	0.01
RAS-like, estrogen-regulated, growth inhibitor	RERG	AW294092	2.5	0.01	2.24	0.01
Ras-related GTP binding D	-	AL138717	3.05	0.01	2.1	0.03
Receptor (G protein-coupled) activity modifying protein 3	RAMP3	NM_005856	2.27	0.01	2.04	0.01
RecQ protein-like (DNA helicase Q1-like)	RECQL	AI962943	2.29	0.01	2.05	0.01
Regenerating islet-derived 1 alpha	REG1A	AF172331	43.64	0.01	16.23	0.01
Regenerating islet-derived 1 beta	REG1B	NM_006507	22.4	0.04	10.66	0.03
Regenerating islet-derived 3 alpha	REG3A	NM_002580	13.54	0.02	8.48	0.01
Regenerating islet-derived family, member 4	REG4	AY007243	18.65	0.01	11.66	0.01
Regulator of G-protein signaling 18	RGS18	AF076642	4.27	0.01	2.86	0.01
Regulator of G-protein signaling 2, 24kDa	RGS2	NM_002923	2.47	0.02	2.28	0.01
Regulator of G-protein signaling 4	RGS4	AL514445	4.76	0.01	3.67	0.01
Regulator of G-protein signaling 5	RGS5	AF159570	2.17	0.04	2.06	0.01
Replication factor C (activator 1)	RFC3	NM_002915	2.82	0.01	2.08	0.01

3, 38kDa						
Reticulon 1	RTN1	NM_021136	2.31	0.03	2.25	0.01
Retinoblastoma 1	RB1	NM_000321	2.7	0.01	2.04	0.02
Retinoic acid induced 2	RAI2	NM_021785	2.6	0.02	2.09	0.01
Retinol binding protein 4, plasma	RBP4	NM_006744	3.1	0.01	2.46	0.01
Retinol dehydrogenase 11 (all-trans/9-cis/11-cis)	RDH11	AF167438	2.52	0.01	2.21	0.01
Rho family GTPase 1	RND1	U69563	3.86	0.01	2.48	0.01
Rho GTPase activating protein 28	ARHGAP28	AI935647	3.04	0.01	2.32	0.01
Rho GTPase activating protein 6	-	NM_001174	3.54	0.01	2.54	0.01
Rho guanine nucleotide exchange factor (GEF) 3	ARHGEF3	NM_019555	2.45	0.01	2.22	0.01
ribonucleotide reductase M2 polypeptide	-	BE966236	3.51	0.01	2.7	0.01
Ribosomal protein L5	RPL5	AL137958	2.79	0.01	2.16	0.01
Ribosomal protein S6 kinase, 90kDa, polypeptide 2	RPS6KA2	AI992251	3.18	0.01	2.63	0.01
Ring finger protein 183	RNF183	BE796148	2.51	0.02	2.54	0.01
RNA binding motif, single stranded interacting protein 1	RBMS1	AI580100	2.4	0.01	2.08	0.01
RNA binding protein with multiple splicing	RBPMS	D84109	3.01	0.01	2.55	0.01
RNA polymerase II associated protein 3	RPAP3	NM_024604	2.64	0.01	2.02	0.03
Roundabout, axon guidance receptor, homolog 1 (Drosophila)	ROBO1	BF059159	3.5	0.02	3.02	0.01
R-spondin 3 homolog (Xenopus laevis)	RSPO3	BF589322	3.37	0.01	2.97	0.01
Runt-related transcription factor 2	RUNX2	AL353944	3.39	0.01	2.58	0.01
S100 calcium binding protein A11	S100A11	NM_005620	2.48	0.01	2.17	0.01
S100 calcium binding protein A11 /// S100 calcium binding protein A11 pseudogene	-	NM_021039	2.61	0.01	2.17	0.01
S100 calcium binding protein A12	S100A12	NM_005621	8.71	0.01	9.03	0.01
S100 calcium binding protein A2	S100A2	NM_005978	2.43	0.01	2.3	0.01
S100 calcium binding protein A8	S100A8	NM_002964	21.7	0.01	17.55	0.01
S100 calcium binding protein A9	S100A9	NM_002965	4.64	0.01	4.48	0.01
S100 calcium binding protein P	S100P	NM_005980	4.06	0.01	4.63	0.01
Sal-like 1 (Drosophila)	SALL1	AU152837	3.61	0.03	2.74	0.01
SAM domain, SH3 domain and nuclear localization signals 1	SAMSN1	AF519621	3.12	0.02	2.41	0.01
Schlafen family member 11	SLFN11	AW003459	2.59	0.01	2.33	0.01
Schlafen family member 12	SLFN12	NM_018042	2.3	0.01	2.14	0.01

SEC11 homolog C (S. cerevisiae)	SEC11C	AF212233	2.7	0.01	2.24	0.01
SEC14-like 1 (S. cerevisiae)	SEC14L1	AI017770	4.15	0.01	3.36	0.01
SEC24 family, member A (S. cerevisiae)	SEC24A	AI080364	2.29	0.01	2.13	0.01
SEC24 family, member D (S. cerevisiae)	SEC24D	NM_014822	4.85	0.01	3.49	0.01
Sec61 alpha 1 subunit (S. cerevisiae)	SEC61A1	AF346602	2.9	0.01	2.25	0.01
Secreted frizzled-related protein 2	SFRP2	AF311912	7.22	0.01	5.94	0.01
Secreted phosphoprotein 1	SPP1	M83248	4	0.01	3.09	0.01
Secretogranin V (7B2 protein)	SCG5	NM_003020	3.41	0.01	2.5	0.01
Secretory leukocyte peptidase inhibitor	SLPI	NM_003064	4.3	0.01	3.68	0.01
Sel-1 suppressor of lin-12-like (C. elegans)	SEL1L	AB020335	3.51	0.01	2.68	0.01
Selectin L	SELL	NM_000450	5.8	0.01	5.19	0.01
Selectin P (granule membrane protein 140kDa, antigen CD62)	SELP	NM_003005	2.75	0.01	2.64	0.01
Selenoprotein M	SELM	BF973568	3.46	0.01	2.81	0.01
Semenogelin I	SEMG1	NM_003007	6.19	0.01	3.69	0.01
Septin 6	6-Sep	AW150913	2.84	0.03	2.17	0.01
Serglycin	SRGN	J03223	3.24	0.02	2.62	0.01
Serine peptidase inhibitor, Kazal type 4	SPINK4	NM_014471	17.04	0.01	11.85	0.01
Serine/threonine/tyrosine interacting protein	STYX	AI652546	3.04	0.01	2.02	0.01
Serpin peptidase inhibitor, clade A (alpha-1 antiproteinase, antitrypsin), member 1	SERPINA 1	AF119873	3.08	0.01	2.5	0.01
Serpin peptidase inhibitor, clade A (alpha-1 antiproteinase, antitrypsin), member 3	SERPINA 3	NM_001085	4.17	0.03	3.05	0.01
Serpin peptidase inhibitor, clade B (ovalbumin), member 5	SERPINB 5	NM_002639	11.33	0.01	10.97	0.01
Serpin peptidase inhibitor, clade B (ovalbumin), member 9	SERPINB 9	BC002538	3.32	0.03	2.59	0.01
Serpin peptidase inhibitor, clade E (nexin, plasminogen activator inhibitor type 1), member 2	SERPINE 2	AL541302	2.59	0.02	2.27	0.01
Serpin peptidase inhibitor, clade G (C1 inhibitor), member 1	SERPING 1	NM_000062	2.04	0.01	2.1	0.01
Serpin peptidase inhibitor, clade I (neuroserpin), member 1	SERPINI1	NM_005025	3.62	0.01	2.77	0.01
Serum amyloid A2	SAA2	M23699	6.76	0.01	5.51	0.01
Serum amyloid A4, constitutive	SAA4	NM_006512	2.63	0.01	2.36	0.01
SH2 domain protein 1A	SH2D1A	AF072930	3.27	0.02	2.77	0.01
SH3 and PX domains 2B	SH3PXD2 B	BG054798	2.56	0.01	2.13	0.01
SH3-domain GRB2-like (endophilin) interacting protein 1	SGIP1	AL136561	2.21	0.01	2.08	0.01
SHC SH2-domain binding protein 1	SHCBP1	NM_024745	3.17	0.01	2.51	0.01

Short chain dehydrogenase/reductase family 16C, member 5	SDR16C5	AI440266	3.66	0.01	3.3	0.01
Shugoshin-like 2 (S. pombe)	SGOL2	N31731	2.4	0.01	2.02	0.01
Sidekick homolog 1, cell adhesion molecule (chicken)	SDK1	AL042166	2.36	0.02	2.1	0.01
Signal peptidase complex subunit 3 homolog (S. cerevisiae)	SPCS3	AL136660	2.6	0.01	2.26	0.01
Signal recognition particle 72kDa	SRP72	AI493872	3.2	0.01	2.29	0.01
Signal recognition particle receptor, B subunit	SRPRB	BF983948	3.05	0.01	2.38	0.01
Signal sequence receptor, alpha	SSR1	AI016620	2.88	0.01	2.26	0.01
Signal sequence receptor, delta (translocon-associated protein delta)	SSR4	NM_006280	2.26	0.01	2.11	0.01
signal sequence receptor, gamma (translocon-associated protein gamma)	-	AW150923	3.04	0.01	2.19	0.01
signal transducer and activator of transcription 1, 91kDa	-	M97935	2.24	0.01	2.61	0.01
Signaling lymphocytic activation molecule family member 1	SLAMF1	NM_003037	2.71	0.01	2.24	0.01
similar to Ig kappa chain V-I region HK102 precursor	-	AJ408433	8.27	0.01	5.41	0.01
Sin3A-associated protein, 30kDa	SAP30	NM_003864	3.12	0.01	2.45	0.01
SLAM family member 7	-	AL121985	5.05	0.01	3.87	0.01
SLAM family member 8	SLAMF8	NM_020125	3.12	0.03	2.79	0.01
Small glutamine-rich tetratricopeptide repeat (TPR)-containing, beta	SGTB	AI376997	3.37	0.01	2.56	0.01
Solute carrier family 1 (glutamate/neutral amino acid transporter), member 4	SLC1A4	BF340083	2.98	0.01	2.3	0.01
Solute carrier family 16, member 14 (monocarboxylic acid transporter 14)	SLC16A14	R15072	2.37	0.04	2.41	0.01
Solute carrier family 16, member 4 (monocarboxylic acid transporter 5)	SLC16A4	NM_004696	3.03	0.01	2.73	0.01
Solute carrier family 18 (vesicular monoamine), member 2	SLC18A2	AI269290	2.5	0.01	2.16	0.01
Solute carrier family 2 (facilitated glucose transporter), member 14	SLC2A14	AA778684	2.66	0.01	2.59	0.01
Solute carrier family 2 (facilitated glucose transporter), member 3	SLC2A3	AI631159	2.57	0.01	2.14	0.01
Solute carrier family 24 (sodium/potassium/calcium exchanger), member 3	SLC24A3	NM_020689	2.38	0.01	2.07	0.01

Solute carrier family 39 (zinc transporter), member 6	SLC39A6	AI635449	3.65	0.01	2.7	0.01
Solute carrier family 4, sodium bicarbonate cotransporter, member 5	SLC4A5	BG324504	2.37	0.01	2.11	0.01
Solute carrier family 6 (amino acid transporter), member 14	SLC6A14	NM_007231	56.81	0.01	51.22	0.01
Solute carrier family 6 (neurotransmitter transporter, taurine), member 6	SLC6A6	BG150485	3.09	0.01	2.79	0.01
Solute carrier family 6 (proline IMINO transporter), member 20	SLC6A20	NM_020208	4.38	0.01	3.71	0.01
Solute carrier family 7 (cationic amino acid transporter, y+ system), member 5	SLC7A5	AB018009	4.07	0.01	3.08	0.01
Solute carrier family 7, (cationic amino acid transporter, y+ system) member 11	SLC7A11	AA488687	4.66	0.01	4.07	0.01
Solute carrier family 8 (sodium/calcium exchanger), member 1	SLC8A1	AI741439	3.21	0.01	2.65	0.01
Solute carrier organic anion transporter family, member 1B3	SLCO1B3	NM_019844	2.28	0.03	2.24	0.02
Sorbitol dehydrogenase	SORD	AV699883	2.66	0.01	2.03	0.01
Sorting nexin 10	SNX10	NM_013322	4.08	0.01	3.02	0.01
Sorting nexin 18	SNX18	AU146771	2.94	0.01	2.11	0.01
SPARC related modular calcium binding 2	SMOC2	AB014737	2.72	0.01	2.19	0.01
Spastic ataxia of Charlevoix-Saguenay (sacsin)	SACS	AI932370	2.78	0.02	2.44	0.01
Spastic paraplegia 20 (Troyer syndrome)	SPG20	AI651603	2.56	0.01	2.23	0.01
SPC25, NDC80 kinetochore complex component, homolog (S. cerevisiae)	SPC25	AF225416	2.74	0.01	2.19	0.01
Sperm associated antigen 4	SPAG4	NM_003116	3.71	0.01	3.29	0.01
Sphingomyelin synthase 1	SGMS1	AI377497	5.15	0.01	3.56	0.01
Sphingomyelin synthase 2	SGMS2	AI963083	3.12	0.01	2.14	0.01
Spondin 2, extracellular matrix protein	SPON2	NM_012445	3.13	0.01	2.91	0.01
Sprouty-related, EVH1 domain containing 1	SPRED1	AW957786	2.58	0.01	2.02	0.01
Squalene epoxidase	SQLE	AF098865	2.4	0.01	2.36	0.01
ST3 beta-galactoside alpha-2,3-sialyltransferase 1	ST3GAL1	AV721528	2.76	0.01	2.51	0.01
ST6 (alpha-N-acetyl-neuraminyl-2,3-beta-galactosyl-1,3)-N-acetylgalactosaminide alpha-2,6-sialyltransferase 5	ST6GALNAC5	NM_030965	2.98	0.01	2.8	0.01
ST8 alpha-N-acetyl-neuraminide alpha-2,8-sialyltransferase 1	ST8SIA1	L32867	2.76	0.02	2.61	0.01
ST8 alpha-N-acetyl-neuraminide alpha-2,8-	ST8SIA4	AA352113	4.75	0.01	3.15	0.01

sialyltransferase 4						
Stanniocalcin 1	STC1	NM_003155	4.46	0.01	3.38	0.01
Stearoyl-CoA desaturase (delta-9-desaturase)	SCD	AA678241	2.33	0.01	2.15	0.01
Stearoyl-CoA desaturase 5	SCD5	AL571375	2.81	0.01	2.06	0.01
Sterile alpha motif and leucine zipper containing kinase AZK	ZAK	AI129320	2.64	0.01	2.2	0.01
Steroid 5 alpha-reductase 3	SRD5A3	BC002480	4.67	0.01	3.17	0.01
Steroid sulfatase (microsomal), isozyme S	STS	AI122754	4.99	0.01	3.96	0.01
Stromal cell-derived factor 2-like 1	SDF2L1	NM_022044	2.48	0.01	2.17	0.01
Succinate receptor 1	SUCNR1	AF348078	2.67	0.02	2.04	0.01
Sulfatase 1	SULF1	AI479175	6.49	0.01	3.9	0.01
Superoxide dismutase 2, mitochondrial	SOD2	W46388	3.86	0.01	3.03	0.01
Suppressor of cytokine signaling 1	SOCS1	AB005043	2.23	0.01	2.05	0.01
Suppressor of cytokine signaling 3	SOCS3	AI244908	4.49	0.01	4.88	0.01
Sushi-repeat-containing protein, X-linked 2	SRPX2	NM_014467	3.15	0.01	3.01	0.01
SWI/SNF related, matrix associated, actin dependent regulator of chromatin, subfamily a, member 1	SMARCA1	AK026426	2.94	0.01	2.42	0.01
Synaptotagmin XI	SYT11	AA626780	2.39	0.01	2.22	0.01
Syndecan 2	SDC2	AI380298	3.59	0.01	2.62	0.01
Syntaxin 11	STX11	AI916948	3.31	0.01	2.76	0.01
Synuclein, alpha interacting protein	SNCAIP	NM_005460	2.33	0.01	2.14	0.01
Tandem C2 domains, nuclear	TC2N	NM_152332	2.83	0.01	2.11	0.01
Tenascin C	TNC	NM_002160	7.53	0.01	6.54	0.01
Tetraspanin 2	TSPAN2	AI743596	3.47	0.01	2.59	0.01
tetratricopeptide repeat domain 7B	-	BE963437	2.5	0.01	2.2	0.01
Tetratricopeptide repeat domain 9	TTC9	AW235608	4.15	0.01	3.48	0.01
Thiamin pyrophosphokinase 1	TPK1	AB028138	3.79	0.01	3	0.01
Thioredoxin domain containing 15	TXNDC15	NM_024715	3.16	0.01	2.58	0.01
Thioredoxin domain containing 5 (endoplasmic reticulum)	TXNDC5	NM_030810	2.88	0.01	2.26	0.01
Thrombomodulin	THBD	NM_000361	3.08	0.01	2.83	0.01
Thrombospondin 2	THBS2	NM_003247	3.95	0.02	2.9	0.01
Thromboxane A synthase 1 (platelet)	TBXAS1	AA044825	2.63	0.01	2.34	0.01
Thy-1 cell surface antigen	THY1	AA218868	2.64	0.01	2.49	0.01
Thymocyte selection pathway associated	TSEPA	BC043608	3.43	0.01	2.2	0.01
Thymosin beta 15a	TMSB15A	NM_021992	3.48	0.01	2.65	0.01
Thyroid hormone receptor interactor 13	TRIP13	NM_004237	2.62	0.01	2.28	0.01

TIMELESS interacting protein	TIPIN	NM_017858	2.9	0.01	2.22	0.01
TIMP metallopeptidase inhibitor 1	TIMP1	NM_003254	5.44	0.01	4.89	0.01
TIMP metallopeptidase inhibitor 3	TIMP3	BF347089	2.39	0.01	2.02	0.01
Tissue factor pathway inhibitor (lipoprotein-associated coagulation inhibitor)	TFPI	AF021834	3.14	0.01	2.64	0.01
Tissue factor pathway inhibitor 2	TFPI2	L27624	10	0.01	7.01	0.01
TNFAIP3 interacting protein 3	TNIP3	NM_024873	15.21	0.01	9.42	0.01
Toll-like receptor 1	TLR1	AL050262	3.59	0.01	2.69	0.01
Toll-like receptor 2	TLR2	NM_003264	2.4	0.02	2.18	0.01
toll-like receptor 4	-	AF177765	2.96	0.01	2.06	0.02
toll-like receptor 8	-	NM_016610	2.87	0.01	2.39	0.01
Toll-like receptor 8	TLR8	AW872374	5.24	0.01	3.93	0.01
Toll-like receptor adaptor molecule 2	TICAM2	AI423165	2.63	0.01	2.2	0.01
TOX high mobility group box family member 2	TOX2	AA211909	3.67	0.01	2.8	0.01
TRAF-interacting protein with forkhead-associated domain	TIFA	AA195074	3.48	0.01	2.52	0.01
Transcobalamin I (vitamin B12 binding protein, R binder family)	TCN1	NM_001062	14.14	0.01	9.94	0.01
Transcribed locus	-	AA059445	2.56	0.01	2.06	0.01
Transcribed locus	-	AA424567	2.38	0.02	2.85	0.01
Transcribed locus	-	AA502609	2.99	0.02	2.29	0.01
Transcribed locus	-	AA643687	4.02	0.01	3.87	0.01
Transcribed locus	-	AA810265	2.51	0.03	2.06	0.01
Transcribed locus	-	AA868461	2.92	0.01	2.64	0.01
Transcribed locus	-	AA903473	2.55	0.02	2.3	0.01
Transcribed locus	-	AI081246	2.29	0.03	2.19	0.01
Transcribed locus	-	AI093327	2.44	0.01	2.14	0.01
Transcribed locus	-	AI141861	6.25	0.01	3.57	0.01
Transcribed locus	-	AI221894	4.33	0.01	3.23	0.01
Transcribed locus	-	AI225238	3.18	0.05	2.59	0.01
Transcribed locus	-	AI266750	2.01	0.02	2.01	0.01
Transcribed locus	-	AI300520	2.46	0.01	2.37	0.01
Transcribed locus	-	AI343467	30.25	0.01	20.33	0.01
Transcribed locus	-	AI363213	2.59	0.01	2.14	0.01
Transcribed locus	-	AI401105	5.31	0.01	3.79	0.01
Transcribed locus	-	AI608902	4.33	0.01	3.63	0.01
Transcribed locus	-	AI654224	3.3	0.01	3.08	0.01
Transcribed locus	-	AI683621	2.5	0.03	2.33	0.01
Transcribed locus	-	AI739241	2.18	0.01	2.09	0.01
Transcribed locus	-	AI754693	4	0.01	2.97	0.01
Transcribed locus	-	AI810826	3.05	0.01	2.44	0.01
Transcribed locus	-	AI873273	2.44	0.03	2.01	0.01
Transcribed locus	-	AI915629	3.87	0.01	3.51	0.01
Transcribed locus	-	AI917494	5.47	0.01	5.34	0.01
Transcribed locus	-	AI922605	2.84	0.01	2.75	0.01

Transcribed locus	-	AI924134	2.52	0.01	2.43	0.01
Transcribed locus	-	AI927941	3.19	0.01	2.16	0.01
Transcribed locus	-	AL041761	2.85	0.01	3.14	0.01
Transcribed locus	-	AL575735	3.83	0.01	3.15	0.01
Transcribed locus	-	AU158251	2.52	0.01	2.38	0.01
Transcribed locus	-	AV655640	3.35	0.01	2.5	0.01
Transcribed locus	-	AV707318	2.57	0.01	2.06	0.01
Transcribed locus	-	AV717590	3.03	0.01	2.57	0.01
Transcribed locus	-	AW043782	3.38	0.01	2.94	0.01
Transcribed locus	-	AW162011	2.27	0.01	2.04	0.01
Transcribed locus	-	AW206419	2.78	0.01	2.33	0.01
Transcribed locus	-	AW242409	2.85	0.01	2.55	0.01
Transcribed locus	-	AW268719	3.55	0.01	2.97	0.01
Transcribed locus	-	AW299568	3.12	0.02	3.21	0.01
Transcribed locus	-	AW504569	2.38	0.03	2.17	0.01
Transcribed locus	-	AW576600	3.53	0.01	2.6	0.01
Transcribed locus	-	AW779917	3.14	0.01	2.04	0.01
Transcribed locus	-	BE501881	3.16	0.01	2.83	0.01
Transcribed locus	-	BE789881	2.8	0.01	2.35	0.01
Transcribed locus	-	BF439063	2.4	0.01	2.1	0.01
Transcribed locus	-	BF508702	2.65	0.01	2.25	0.01
Transcribed locus	-	BF513674	5.87	0.01	4.14	0.01
Transcribed locus	-	BG112263	3.3	0.01	2.68	0.01
Transcribed locus	-	BG250585	3.14	0.01	2.73	0.01
Transcribed locus	-	BG401950	3	0.01	2.22	0.01
Transcribed locus	-	BG484552	2.43	0.01	2.16	0.01
Transcribed locus	-	BG540494	2.74	0.01	2.26	0.01
Transcribed locus	-	N22766	2.41	0.01	2.11	0.01
Transcribed locus, strongly similar to NP_001094380.1 hypothetical protein LOC653820 [Homo sapiens]	-	AL135396	2.75	0.01	2.1	0.01
Transcribed locus, strongly similar to XP_537800.1 PREDICTED: similar to 60S ribosomal protein L7a [Canis familiaris]	-	AF130059	2.71	0.01	2.31	0.01
Transcription elongation factor A (SII)-like 7	TCEAL7	BF591534	3.95	0.01	2.82	0.01
Transcription factor 4	TCF4	BE857360	2.98	0.01	2.58	0.01
Transcription factor AP-2 gamma (activating enhancer binding protein 2 gamma)	TFAP2C	U85658	3.2	0.01	2.46	0.01
Transcription factor EC	TFEC	AL110232	2.67	0.01	2.02	0.01
Transcriptional regulating factor 1	TRERF1	BF724270	2.45	0.03	2.03	0.01
Transforming growth factor, beta 2	TGFB2	AU145950	2.12	0.01	2.17	0.01
Transforming growth factor, beta-induced, 68kDa	TGFBI	NM_000358	3.23	0.01	2.84	0.01
Transgelin	TAGLN	BC010946	2.27	0.01	2.08	0.01

transglutaminase 2 (C polypeptide, protein-glutamine-gamma-glutamyltransferase)	-	AL031651	6.1	0.01	4.52	0.01
transmembrane 4 L six family member 1	-	M90657	3.56	0.01	2.64	0.01
Transmembrane and tetratricopeptide repeat containing 1	TMTC1	AF319520	3.44	0.01	2.94	0.01
Transmembrane protein 158	TMEM158	BF062629	8.01	0.01	5.83	0.01
Transmembrane protein 163	TMEM163	AF255647	4.65	0.02	3.76	0.01
Transmembrane protein 165	TMEM165	NM_018475	2.86	0.01	2.32	0.01
Transmembrane protein 45A	TMEM45A	NM_018004	9.1	0.01	5.01	0.01
Transmembrane protein 48	TMEM48	NM_018087	2.63	0.01	2.01	0.01
Transmembrane protein 64	TMEM64	BF732480	3.42	0.01	2.45	0.01
Transmembrane protein 92	TMEM92	AI990471	2.48	0.01	2.04	0.01
Trefoil factor 2	TFF2	NM_005423	2.77	0.01	2.41	0.01
Tribbles homolog 2 (Drosophila)	TRIB2	BC002637	2.65	0.01	2.27	0.01
Triggering receptor expressed on myeloid cells 1	TREM1	NM_018643	4.88	0.01	3.68	0.01
Tripartite motif-containing 29	TRIM29	NM_012101	2.48	0.01	2.44	0.01
Tripartite motif-containing 7	TRIM7	AF220032	2.25	0.01	2	0.01
tropomyosin 3	-	AF362887	2.31	0.01	2.05	0.01
Tryptase alpha/beta 1	TPSAB1	AF206665	2.04	0.02	2.08	0.01
tryptase beta 2	-	AF099143	2.16	0.01	2.05	0.01
Tryptophan 2,3-dioxygenase	TDO2	NM_005651	6.86	0.01	4.52	0.01
Tryptophanyl-tRNA synthetase	WARS	M61715	2.93	0.01	2.56	0.01
TTK protein kinase	TTK	NM_003318	2.57	0.01	2	0.01
Tubulin, beta 6	TUBB6	BC002654	2.37	0.01	2.38	0.01
Tumor necrosis factor (ligand) superfamily, member 11	TNFSF11	AF053712	2.61	0.04	2.79	0.01
tumor necrosis factor (ligand) superfamily, member 13b	-	AW151360	2.76	0.03	2.38	0.01
Tumor necrosis factor receptor superfamily, member 11b	TNFRSF11B	BF433902	3.89	0.01	3.24	0.01
Tumor necrosis factor receptor superfamily, member 17	TNFRSF17	NM_001192	7.51	0.01	4.13	0.01
Tumor necrosis factor receptor superfamily, member 6b, decoy	TNFRSF6B	NM_003823	3.82	0.01	3.38	0.01
Tumor necrosis factor, alpha-induced protein 6	TNFAIP6	AW188198	5.3	0.01	4.11	0.01
Tumor necrosis factor, alpha-induced protein 8	TNFAIP8	BC005352	2.87	0.03	2.28	0.01
Tumor protein p63 regulated 1	TPRG1	AW629527	3.21	0.01	2.93	0.01
Tumor suppressor candidate 3	TUSC3	AI884858	2.78	0.01	2.81	0.01
tumor-associated calcium signal transducer 2	-	J04152	6.19	0.01	4.06	0.01
Twist homolog 1 (Drosophila)	TWIST1	X99268	3.08	0.01	2.66	0.01
Twisted gastrulation homolog 1 (Drosophila)	TWSG1	AA195009	2.88	0.01	2.28	0.01
Two transmembrane domain family member A	-	N66694	2.84	0.01	2.44	0.01
Tyrosinase-related protein 1	TYRP1	NM_000550	6.58	0.01	5.63	0.01

Tyrosylprotein sulfotransferase 2	TPST2	NM_003595	2.8	0.01	2.31	0.01
Ubiquitin associated and SH3 domain containing, B	UBASH3B	AI418293	2.82	0.01	2.13	0.01
Ubiquitin D	UBD	NM_006398	2.36	0.01	2.52	0.01
Ubiquitin-conjugating enzyme E2D 1 (UBC4/5 homolog, yeast)	UBE2D1	AL545760	2.94	0.01	2.04	0.01
Ubiquitin-conjugating enzyme E2L 6	UBE2L6	NM_004223	2.06	0.01	2.04	0.01
Ubiquitin-like domain containing CTD phosphatase 1	UBLCP1	BF965546	3.01	0.01	2.27	0.01
Ubiquitin-like with PHD and ring finger domains 1	UHRF1	AK025578	2.7	0.01	2.11	0.01
UDP-N-acetyl-alpha-D-galactosamine:polypeptide N-acetylgalactosaminyltransferase 2 (GalNAc-T2)	GALNT2	AL525086	2.9	0.01	2.32	0.01
UDP-N-acetyl-alpha-D-galactosamine:polypeptide N-acetylgalactosaminyltransferase 5 (GalNAc-T5)	GALNT5	AI633503	2.33	0.01	2.14	0.01
vanin 1	-	BG120535	5.89	0.01	5.17	0.01
Vanin 2	VNN2	NM_004665	2.91	0.02	2.43	0.01
Vascular endothelial growth factor C	VEGFC	U58111	3.19	0.01	2.87	0.01
Versican	VCAN	BF218922	4.33	0.01	3.63	0.01
Vesicle-associated membrane protein 4	VAMP4	BC005974	3.82	0.01	2.56	0.01
Vestigial like 3 (Drosophila)	VGLL3	AI754423	2.33	0.01	2.12	0.01
Visinin-like 1	VSNL1	NM_003385	4.15	0.01	2.93	0.01
Vitelline membrane outer layer 1 homolog (chicken)	VMO1	AA977975	2.86	0.01	2.52	0.01
V-kit Hardy-Zuckerman 4 feline sarcoma viral oncogene homolog	KIT	NM_000222	3.43	0.01	2.78	0.01
v-maf musculoaponeurotic fibrosarcoma oncogene homolog F (avian)	-	AL021977	2.93	0.01	2.59	0.01
Von Willebrand factor	VWF	NM_000552	2.27	0.01	2.18	0.01
V-set and immunoglobulin domain containing 1	VSIG1	AW085312	2.37	0.02	2.61	0.01
V-yes-1 Yamaguchi sarcoma viral related oncogene homolog	LYN	AI356412	2.67	0.01	2.16	0.01
WAS/WASL interacting protein family, member 1	WIPF1	AI005043	2.7	0.03	2.16	0.02
WAS/WASL interacting protein family, member 1	WIPF1	AW058622	2.73	0.05	2.36	0.01
Wingless-type MMTV integration site family, member 2B	WNT2B	NM_024494	2.63	0.01	2.15	0.01
Wingless-type MMTV integration site family, member 4	WNT4	NM_030761	3.01	0.01	2.19	0.01
Wingless-type MMTV	WNT5A	AI968085	5.76	0.01	3.82	0.01

integration site family, member 5A						
WNT1 inducible signaling pathway protein 1	WISP1	NM_003882	5.17	0.01	4.47	0.01
X-box binding protein 1	XBP1	NM_005080	3.15	0.01	2.63	0.01
Yip1 domain family, member 5	YIPF5	AW001618	3.2	0.01	2.21	0.01
Zinc finger CCCH-type containing 12A	ZC3H12A	NM_025079	2.99	0.01	2.77	0.01
Zinc finger E-box binding homeobox 1	ZEB1	NM_030751	3.07	0.01	2.85	0.01
Zinc finger E-box binding homeobox 2	ZEB2	NM_014795	2.42	0.01	2.09	0.02
Zinc finger protein 521	ZNF521	AF141339	3.36	0.01	2.63	0.01
Zinc finger protein, multitype 2	ZFPM2	NM_012082	5.37	0.01	3.57	0.01
Zinc finger, BED-type containing 2	ZBED2	NM_024508	3.34	0.01	2.99	0.01
Zymogen granule protein 16 homolog B (rat)	ZG16B	AI559190	2.17	0.05	2.32	0.01

Supplementary Table 4. Pediatric UC commonly down regulated genes

Gene Title	Gene ID	Gene Identifier	9686UC		10616 UC	
			fold	p	Fold	p
3-hydroxy-3-methylglutaryl-Coenzyme A synthase 2 (mitochondrial)	HMGCS2	NM_005518	-14.87	0.01	-13.68	0.01
3-hydroxybutyrate dehydrogenase, type 1	BDH1	BC005844	-2.22	0.01	-2.23	0.01
4-aminobutyrate aminotransferase	ABAT	AF237813	-2.21	0.01	-2.21	0.01
5-hydroxytryptamine (serotonin) receptor 4	HTR4	AJ131724	-2.61	0.02	-2.25	0.01
5'-nucleotidase, cytosolic II	NT5C2	BF114733	-3.74	0.01	-2.46	0.01
A kinase (PRKA) anchor protein 1	AKAP1	BC000729	-2.85	0.01	-2.36	0.01
Acetyl-Coenzyme A carboxylase beta	ACACB	AI057637	-2.52	0.01	-2.27	0.01
Activin A receptor, type IC	ACVR1C	BC030101	-2.04	0.02	-2.22	0.01
Acyl-CoA synthetase family member 2	ACSF2	NM_025149	-3.01	0.01	-2.58	0.01
Acyl-CoA synthetase short-chain family member 2	ACSS2	AK000162	-2.5	0.01	-2.23	0.01
Acyl-Coenzyme A dehydrogenase, C-2 to C-3 short chain	ACADS	NM_000017	-2.45	0.01	-2.43	0.01
Acyl-Coenzyme A oxidase 1, palmitoyl	ACOX1	BF435852	-3.48	0.01	-3.22	0.01
Adaptor protein, phosphotyrosine interaction, PH domain and leucine zipper containing 2	APPL2	NM_018171	-2.46	0.01	-2.69	0.01
Advillin	AVIL	NM_006576	-4.42	0.01	-2.92	0.01
AFG3 ATPase family gene 3-like 2 (yeast)	AFG3L2	AU147380	-3.38	0.01	-2.25	0.01
Alanine-glyoxylate aminotransferase 2-like 2	AGXT2L2	AI762154	-3.89	0.01	-2.37	0.01
Alanyl (membrane) aminopeptidase	ANPEP	NM_001150	-3.95	0.01	-5	0.01
Alcohol dehydrogenase 1C (class I), gamma polypeptide	ADH1C	NM_000669	-2.68	0.02	-3.65	0.01

Alpha-methylacyl-CoA racemase	AMACR	AA888589	-2.48	0.01	-2.48	0.01
Aminomethyltransferase	AMT	NM_000481	-2.66	0.01	-2.05	0.01
Amylase, alpha 1A (salivary)	AMY1A	NM_004038	-5.07	0.01	-3.12	0.01
Ankyrin 3, node of Ranvier (ankyrin G)	ANK3	AI743588	-9.07	0.01	-5.63	0.01
Anoctamin 9	ANO9	AW084755	-3.59	0.01	-2.36	0.01
APOBEC1 complementation factor	A1CF	NM_014576	-3.67	0.01	-2.89	0.01
Apolipoprotein B mRNA editing enzyme, catalytic polypeptide-like 3B	APOBEC3B	NM_004900	-2.78	0.01	-2.8	0.01
Apoptosis-inducing factor, mitochondrion-associated, 3	AIFM3	AA468769	-6.46	0.01	-3.92	0.01
Aquaporin 8	AQP8	NM_001169	-28.58	0.01	-36.55	0.01
Aspartoacylase (Canavan disease)	ASPA	NM_000049	-2.47	0.01	-2.28	0.01
ATP synthase, H+ transporting, mitochondrial F1 complex, gamma polypeptide 1	ATP5C1	BG232034	-3.16	0.01	-2.07	0.01
ATP/GTP binding protein-like 2	AGBL2	NM_024783	-2.83	0.01	-2.39	0.01
ATPase, aminophospholipid transporter (APLT), class I, type 8A, member 1	ATP8A1	AB013452	-2.03	0.05	-2.07	0.01
ATPase, class V, type 10B	ATP10B	NM_025153	-3.09	0.01	-2.05	0.01
ATP-binding cassette, sub-family B (MDR/TAP), member 1	ABCB1	AF016535	-4.98	0.01	-4.04	0.01
ATP-binding cassette, sub-family C (CFTR/MRP), member 13	-	NM_172025	-5.84	0.01	-3.33	0.01
ATP-binding cassette, sub-family C (CFTR/MRP), member 3	ABCC3	BF515888	-5.45	0.01	-3.01	0.01
ATP-binding cassette, sub-family C (CFTR/MRP), member 6	ABCC6	AI074459	-2.98	0.01	-2.36	0.01
ATP-binding cassette, sub-family	ABCG2	AF098951	-8.52	0.01	-9.93	0.01

G (WHITE), member 2						
Bestrophin 2	BEST2	NM_017682	-2.6	0.01	-2.36	0.01
Bestrophin 4	BEST4	NM_153274	-4.91	0.01	-4.22	0.01
BTG family, member 3	-	AL049332	-2.32	0.01	-2.05	0.01
Calcineurin B homologous protein 2	CHP2	NM_022097	-5.34	0.01	-5.95	0.01
calcium channel, voltage-dependent, beta 2 subunit	-	U80764	-2.99	0.01	-2.05	0.01
calcium/calmodulin-dependent protein kinase II inhibitor 1	-	AW162846	-3.77	0.01	-3.34	0.01
Calpain 13	CAPN13	AI762355	-2.48	0.03	-2	0.01
Calpain 3, (p94)	CAPN3	AF127764	-4.57	0.01	-2.43	0.01
Carbonic anhydrase I	CA1	NM_001738	-7.64	0.01	-6.7	0.01
Carbonic anhydrase IV	CA4	NM_000717	-2.1	0.01	-2.06	0.01
Carbonic anhydrase XII	CA12	BC000278	-2.42	0.01	-2.46	0.01
Carbonyl reductase 4	CBR4	NM_032783	-3.8	0.01	-2.25	0.01
Carboxylesterase 2 (intestine, liver)	CES2	D50579	-3.19	0.01	-2.87	0.01
Carboxylesterase 3	CES3	NM_024922	-2.59	0.01	-2.33	0.01
Carboxypeptidase M	CPM	BE878495	-3.07	0.01	-2.46	0.01
Carcinoembryonic antigen-related cell adhesion molecule 1 (biliary glycoprotein)	CEACAM1	M69176	-6.2	0.01	-3.43	0.01
Carnitine palmitoyltransferase 1B (muscle)	CPT1B	U62733	-2.94	0.01	-2.02	0.01
CD177 molecule	CD177	NM_020406	-6.09	0.01	-6.21	0.01
CDC14 cell division cycle 14 homolog A (S. cerevisiae)	CDC14A	AF064103	-3.21	0.01	-2.24	0.01
CDC42 binding protein kinase alpha (DMPK-like)	CDC42BPA	NM_003607	-3.33	0.01	-2.46	0.01
CDKN2B antisense RNA (non-protein coding)	CDKN2BAS	BC038540	-3.13	0.01	-2.95	0.01
CDNA clone IMAGE:4346813	-	BF346665	-5.24	0.01	-3.18	0.01
CDNA clone IMAGE:4794632	-	BC040647	-3.93	0.01	-2.88	0.01
CDNA clone IMAGE:4801360	-	AA630955	-3.51	0.01	-2.52	0.01
CDNA clone IMAGE:5175565	-	AW003845	-3.27	0.01	-2.01	0.01

CDNA clone IMAGE:5199989	-	BC030211	-3.76	0.01	-2.01	0.04
CDNA clone IMAGE:5261865	-	BQ710453	-2.51	0.01	-2	0.01
CDNA clone IMAGE:5272221	-	AI524619	-3.9	0.01	-2.56	0.01
CDNA clone IMAGE:5274509	-	BC037883	-2.78	0.01	-2.08	0.01
CDNA clone IMAGE:5286225	-	BC042961	-4.34	0.01	-2.73	0.01
CDNA FLJ10171 fis, clone HEMBA1003807	-	AU145070	-3.1	0.01	-2.07	0.01
CDNA FLJ11415 fis, clone HEMBA1000942	-	AK021477	-3.42	0.01	-2.08	0.01
CDNA FLJ11687 fis, clone HEMBA1004960	-	AK021749	-5.31	0.01	-2.92	0.01
CDNA FLJ11757 fis, clone HEMBA1005606	-	AU145719	-3.98	0.01	-2.29	0.01
CDNA FLJ12024 fis, clone HEMBB1001797	-	AU146893	-4.12	0.01	-2.11	0.01
CDNA FLJ12050 fis, clone HEMBB1002002	-	AK022112	-4.02	0.01	-2.44	0.01
CDNA FLJ12088 fis, clone HEMBB1002545	-	AU147152	-4.43	0.01	-3	0.01
CDNA FLJ12284 fis, clone MAMMA1001757	-	AU147805	-3.82	0.01	-2.36	0.01
CDNA FLJ12285 fis, clone MAMMA1001764	-	AU147809	-4.23	0.01	-2.34	0.01
CDNA FLJ12288 fis, clone MAMMA1001783	-	AK022350	-7.17	0.01	-4.49	0.01
CDNA FLJ12308 fis, clone MAMMA1001931	-	AU147861	-3.26	0.01	-2.09	0.01
CDNA FLJ12727 fis, clone NT2RP2000027	-	BF110792	-3.35	0.01	-2.07	0.02
CDNA FLJ13274 fis, clone OVARC1001029	-	AU155112	-4.22	0.01	-2.85	0.01
CDNA FLJ13464 fis, clone PLACE1003478	-	AI740629	-2.96	0.01	-2.41	0.01
CDNA FLJ13694 fis, clone PLACE2000115	-	AU158358	-3.93	0.01	-2.15	0.03

CDNA FLJ20031 fis, clone ADSU02180	-	BF726849	-2.55	0.01	-2.2	0.01
CDNA FLJ20078 fis, clone COL02974	-	AK000085	-2.49	0.01	-2.54	0.01
CDNA FLJ20099 fis, clone COL04544	-	AK000106	-4.65	0.01	-2.61	0.01
CDNA FLJ20112 fis, clone COL05405	-	AK000119	-3.63	0.01	-2.08	0.01
CDNA FLJ20486 fis, clone KAT08039	-	AK000493	-2.92	0.01	-2.66	0.01
CDNA FLJ20770 fis, clone COL06509	-	AK000777	-3.81	0.01	-2.27	0.01
CDNA FLJ20771 fis, clone COL06394	-	AK000778	-5.37	0.01	-2.98	0.01
CDNA FLJ20788 fis, clone COL02074	-	AK000795	-11.56	0.01	-5.28	0.01
CDNA FLJ23646 fis, clone COL03258	-	AK074226	-3.29	0.01	-2.14	0.01
CDNA FLJ25345 fis, clone TST01118	-	AA669135	-3.6	0.01	-2.44	0.01
CDNA FLJ26461 fis, clone KDN03867	-	AA968447	-3.41	0.01	-2.01	0.01
CDNA FLJ26512 fis, clone KDN07513	-	H57166	-2.77	0.01	-2.54	0.01
CDNA FLJ30409 fis, clone BRACE2008615	-	BQ027635	-3.37	0.01	-2.13	0.01
CDNA FLJ32697 fis, clone TESTI2000372	-	AK057259	-3.78	0.01	-2.65	0.01
CDNA FLJ33679 fis, clone BRAWH2002352	-	AK025137	-3.19	0.01	-2.11	0.03
CDNA FLJ34358 fis, clone FEBRA2013905	-	AA496222	-2.24	0.01	-2.08	0.01
CDNA FLJ34475 fis, clone HLUNG2003716, moderately similar to RETROVIRUS-RELATED ENV POLYPROTEIN	-	AK091794	-3.72	0.01	-2.24	0.01
CDNA FLJ36375 fis, clone THYMU2008226	-	AA632049	-2.84	0.01	-2.05	0.01
CDNA FLJ37319 fis, clone BRAMY2018027	-	AA737437	-8.02	0.01	-3.23	0.01
CDNA FLJ37584 fis, clone BRCOC2004950	-	AW029203	-3.47	0.01	-2.02	0.01
CDNA FLJ39306 fis, clone OCBBF2013123	-	AI571166	-4.58	0.01	-2.78	0.01

CDNA FLJ39805 fis, clone SPLEN2007951	-	CA444630	-3.59	0.01	-2.24	0.01
CDNA FLJ40647 fis, clone THYMU2017522	-	AI814750	-3.34	0.01	-2.42	0.01
CDNA FLJ42410 fis, clone BLADE2000938	-	AW975013	-3.85	0.01	-2.28	0.01
CDNA: FLJ21211 fis, clone COL00485	-	AK024864	-3.12	0.01	-2.26	0.01
CDNA: FLJ21228 fis, clone COL00739	-	AK024881	-4.57	0.01	-2.31	0.01
CDNA: FLJ21244 fis, clone COL01174	-	AK024897	-2.56	0.01	-2.08	0.01
CDNA: FLJ21254 fis, clone COL01317	-	AK024907	-3.15	0.01	-2.09	0.01
CDNA: FLJ21303 fis, clone COL02107	-	AK024956	-5.41	0.01	-3.05	0.01
CDNA: FLJ21345 fis, clone COL02694	-	AK024998	-2.63	0.02	-2.36	0.01
CDNA: FLJ21389 fis, clone COL03455	-	AK025042	-14.51	0.01	-8.16	0.01
CDNA: FLJ21391 fis, clone COL03479	-	AK025044	-9.77	0.01	-4.88	0.01
CDNA: FLJ21428 fis, clone COL04203	-	AK025081	-4.2	0.01	-2.4	0.01
CDNA: FLJ21556 fis, clone COL06353	-	AA528157	-2.78	0.01	-2.77	0.01
CDNA: FLJ21565 fis, clone COL06463	-	AK025218	-4.96	0.01	-3.16	0.01
CDNA: FLJ21635 fis, clone COL08233, highly similar to AF131819 Homo sapiens clone 24838 mRNA sequence	-	AA480392	-3.91	0.01	-2.1	0.02
CDNA: FLJ22614 fis, clone HSI05089	-	AK026267	-6.28	0.01	-2.34	0.01
CDNA: FLJ23163 fis, clone LNG09751	-	AK026816	-3.4	0.01	-2.11	0.02
CDNA: FLJ23240 fis, clone COL01276	-	AK026893	-7.88	0.01	-4.32	0.01
CDNA: FLJ23261 fis, clone COL05862	-	AK026914	-3.77	0.01	-2.16	0.01
centrosomal protein 68kDa	-	BF589179	-3.4	0.01	-2.09	0.01
Chloride channel 2	CLCN2	NM_004366	-2.2	0.01	-2.1	0.01
Choline kinase alpha	CHKA	NM_001277	-2.98	0.01	-2.19	0.01
Choline phosphotransferase 1	CHPT1	AK025141	-3.36	0.01	-2.43	0.01
Chromosome 1	C1orf161	NM_152367	-2.72	0.01	-2.12	0.01

open reading frame 161						
Chromosome 1 open reading frame 63	C1orf63	AF247168	-3.61	0.01	-2.22	0.01
Chromosome 10 open reading frame 116	C10orf116	NM_006829	-4.98	0.01	-4.35	0.01
chromosome 15 open reading frame 5	-	NM_030944	-3.52	0.01	-2	0.02
Chromosome 15 open reading frame 52	C15orf52	BE673226	-3	0.01	-2.18	0.01
Chromosome 2 open reading frame 88	C2orf88	BC005083	-2.17	0.01	-2.2	0.01
Chromosome 6 open reading frame 124	C6orf124	AB016898	-2.87	0.01	-2.14	0.01
Chromosome 6 open reading frame 26	C6orf26	BE674960	-3.5	0.01	-2.13	0.01
Chromosome 9 open reading frame 86	C9orf86	BE208843	-3.61	0.01	-2.47	0.01
Cingulin	CGN	AI768894	-3.5	0.01	-3.1	0.01
CKLF-like MARVEL transmembrane domain containing 4	CMTM4	AA191708	-2.81	0.01	-2.25	0.01
Claudin 8	CLDN8	AL049977	-7.65	0.02	-6.22	0.01
Clone 23948 mRNA sequence	-	U79293	-4.07	0.01	-2.33	0.01
Clone FP18821 unknown mRNA	-	AF289551	-5.17	0.01	-3.07	0.01
Coiled-coil domain containing 64B	CCDC64B	AW139399	-3.37	0.01	-2.54	0.01
Consensus includes gb:AA195276 /FEA=EST /DB_XREF=gi:1784976 /DB_XREF=est:zr34b03.s1 /CLONE=IMAGE:665261 /UG=Hs.263858 ESTs, Moderately similar to B34087 hypothetical protein H.sapiens	-	AA195276	-3.19	0.01	-2.48	0.01
Consensus includes gb:AA521056 /FEA=EST /DB_XREF=gi:2261	-	AA521056	-4.04	0.01	-2.28	0.02

599 /DB_XREF=est:aa71e05.s1 /CLONE=IMAGE:826400 /UG=Hs.6179 DEADH (Asp-Glu-Ala-AspHis) box polypeptide 17 (72kD)						
Consensus includes gb:AA704163 /FEA=EST /DB_XREF=gi:2714081 /DB_XREF=est:zj77a08.s1 /CLONE=IMAGE:460886 /UG=Hs.272101 ESTs	-	AA704163	-4.17	0.01	-2.35	0.01
Consensus includes gb:AA913635 /FEA=EST /DB_XREF=gi:3053027 /DB_XREF=est:om94f01.s2 /CLONE=IMAGE:1554841 /UG=Hs.326413 Homo sapiens cDNA FLJ20812 fis, clone ADSE01316	-	AA913635	-2.87	0.01	-2.17	0.01
Consensus includes gb:AA993566 /FEA=EST /DB_XREF=gi:3180111 /DB_XREF=est:ot96g10.s1 /CLONE=IMAGE:1624674 /UG=Hs.159983 ESTs	-	AA993566	-4.65	0.01	-2.25	0.01
Consensus includes gb:AI148006 /FEA=EST /DB_XREF=gi:3675688 /DB_XREF=est:qg62e10.s1 /CLONE=IMAGE:1839786 /UG=Hs.222120 ESTs	-	AI148006	-5.5	0.01	-2.91	0.01

Consensus includes gb:AI251870 /FEA=EST /DB_XREF=gi:3848399 /DB_XREF=est:qu78a11.x1 /CLONE=IMAGE:1978172 /UG=Hs.188898 ESTs	-	AI251870	-2.98	0.01	-2.02	0.02
Consensus includes gb:AI494291 /FEA=EST /DB_XREF=gi:4395294 /DB_XREF=est:qy98d11.x1 /CLONE=IMAGE:2020053 /UG=Hs.111977 ESTs	-	AI494291	-4.02	0.01	-2.37	0.01
Consensus includes gb:AI694598 /FEA=EST /DB_XREF=gi:4971938 /DB_XREF=est:wd88g05.x1 /CLONE=IMAGE:2338712 /UG=Hs.202126 ESTs	-	AI694598	-2.62	0.01	-2.04	0.01
Consensus includes gb:AI821404 /FEA=EST /DB_XREF=gi:5440483 /DB_XREF=est:ye17h09.x5 /CLONE=IMAGE:118049 /UG=Hs.227505 ESTs, Weakly similar to ALU7_HUMAN ALU SUBFAMILY SQ SEQUENCE CONTAMINATION WARNING ENTRY H.sapiens	-	AI821404	-6.82	0.01	-3.74	0.01
Consensus includes gb:AU155612 /FEA=EST /DB_XREF=gi:11017133	-	AU155612	-3.32	0.01	-2.09	0.01

/DB_XREF=est:AU155612 /CLONE=PLACE1000417 /UG=Hs.269545 ESTs, Weakly similar to ALUB_HUMAN !!!! ALU CLASS B WARNING ENTRY !!! H.sapiens						
Consensus includes gb:AU156822 /FEA=EST /DB_XREF=gi:11018343 /DB_XREF=est:AU156822 /CLONE=PLACE1004838 /UG=Hs.287577 Homo sapiens cDNA FLJ13503 fis, clone PLACE1004838	-	AU156822	-3.66	0.01	-2.22	0.01
Consensus includes gb:AV700081 /FEA=EST /DB_XREF=gi:10302052 /DB_XREF=est:AV700081 /CLONE=GKCFED12 /UG=Hs.284614 ESTs	-	AV700081	-4.4	0.01	-2.78	0.01
Consensus includes gb:AV735587 /FEA=EST /DB_XREF=gi:10853168 /DB_XREF=est:AV735587 /CLONE=CBDAAD08 /UG=Hs.249890 ESTs	-	AV735587	-5.35	0.01	-2.77	0.01
Consensus includes gb:AW173166 /FEA=EST /DB_XREF=gi:6439114 /DB_XREF=est:xj84b11.x1 /CLONE=IMAGE:2663901 /UG=Hs.243468 ESTs	-	AW173166	-4.03	0.01	-2.65	0.01

Consensus includes gb:AW182675 /FEA=EST /DB_XREF=gi:6451135 /DB_XREF=est:xj45d04.x1 /CLONE=IMAGE:2660167 /UG=Hs.187820 ESTs	-	AW182675	-4.69	0.01	-2.35	0.01
Consensus includes gb:AW885748 /FEA=EST /DB_XREF=gi:8047760 /DB_XREF=est:RC4-OT0071-240400-014-d05 /UG=Hs.257862 ESTs	-	AW885748	-6.84	0.01	-3.52	0.01
Consensus includes gb:BE503118 /FEA=EST /DB_XREF=gi:9705526 /DB_XREF=est:hz83f11.x1 /CLONE=IMAGE:3214605 /UG=Hs.257852 ESTs	-	BE503118	-3.67	0.01	-2.06	0.01
Consensus includes gb:BF002339 /FEA=EST /DB_XREF=gi:10702614 /DB_XREF=est:7h22c09.x1 /CLONE=IMAGE:3316720 /UG=Hs.158528 ESTs	-	BF002339	-3.42	0.01	-2.32	0.01
Consensus includes gb:H57111 /FEA=EST /DB_XREF=gi:1009943 /DB_XREF=est:yr09e12.s1 /CLONE=IMAGE:204814 /UG=Hs.221132 ESTs	-	H57111	-4.14	0.01	-2.21	0.01
Consensus includes gb:H58000	-	H58000	-3.76	0.01	-2.07	0.01

/FEA=EST /DB_XREF=gi:1010832 /DB_XREF=est:yr05g06.s1 /CLONE=IMAGE:204442 /UG=Hs.269837 ESTs						
Consensus includes gb:H71242 /FEA=EST /DB_XREF=gi:1043058 /DB_XREF=est:ys12g09.s1 /CLONE=IMAGE:214624 /UG=Hs.117864 ESTs	-	H71242	-3.74	0.01	-2.2	0.01
Consensus includes gb:N58278 /FEA=EST /DB_XREF=gi:1202168 /DB_XREF=est:yv68b02.s1 /CLONE=IMAGE:247851 /UG=Hs.269400 ESTs	-	N58278	-3.08	0.01	-2.1	0.01
Consensus includes gb:R39769 /FEA=EST /DB_XREF=gi:797225 /DB_XREF=est:yf58b03.s1 /CLONE=IMAGE:26267 /UG=Hs.206088 ESTs, Moderately similar to ALU8_HUMAN ALU SUBFAMILY SX SEQUENCE CONTAMINATION WARNING ENTRY H.sapiens	-	R39769	-3.66	0.01	-2.34	0.01
Consensus includes gb:R55749 /FEA=EST /DB_XREF=gi:825824 /DB_XREF=est:yg89b03.s1 /CLONE=IMAGE:40	-	R55749	-2.17	0.02	-2.13	0.01

720 /UG=Hs.26454 ESTs, Weakly similar to B34087 hypothetical protein H.sapiens						
Contactin 3 (plasmacytoma associated)	CNTN3	BE221817	-4.26	0.01	-4.2	0.01
Creatine kinase, brain	CKB	NM_001823	-6.81	0.01	-4.15	0.01
Cyclin-dependent kinase 3	CDK3	AI885421	-4.78	0.01	-3.2	0.01
Cyclin-dependent kinase inhibitor 2B (p15, inhibits CDK4)	CDKN2B	AW444761	-3.48	0.01	-3.65	0.01
Cysteine sulfinic acid decarboxylase	CSAD	NM_015989	-3.72	0.01	-2.07	0.01
Cytochrome P450, family 2, subfamily B, polypeptide 6	CYP2B6	NM_000767	-3.93	0.01	-3.53	0.01
Cytochrome P450, family 2, subfamily C, polypeptide 9	CYP2C9	AV652420	-2.73	0.01	-2.29	0.01
Cytochrome P450, family 3, subfamily A, polypeptide 5	CYP3A5	X90579	-3.64	0.01	-2.23	0.01
Cytochrome P450, family 4, subfamily F, polypeptide 12	CYP4F12	NM_023944	-2.51	0.01	-2.46	0.01
Cytochrome P450, family 4, subfamily F, polypeptide 2	CYP4F2	D26480	-2.49	0.01	-2.24	0.01
DEAD (Asp-Glu-Ala-Asp) box polypeptide 17	DDX17	AW188131	-3.6	0.01	-2.09	0.01
DEAQ box RNA-dependent ATPase 1	DQX1	H58606	-2.99	0.01	-2.41	0.01
Death-associated protein kinase 2	DAPK2	AK026801	-2.21	0.01	-2.1	0.01
Defensin, beta 1	DEFB1	U73945	-3.66	0.01	-4.03	0.01
Dehydrogenase/reductase (SDR family) member 11	DHRS11	NM_024308	-3.99	0.01	-3.82	0.01
DENN/MADD domain containing 1B	DENND1B	AL831839	-4.56	0.01	-2.78	0.01
DEP domain containing 7	DEPDC7	AJ245600	-2.85	0.01	-2.39	0.01
Desmocollin 2	DSC2	BF196457	-2.67	0.02	-2.39	0.01
dimethylarginine dimethylaminohydrolase 2	-	AJ012008	-2.61	0.01	-2.28	0.01
Dipeptidyl-peptidase 10	DPP10	AL538781	-2.93	0.01	-3.69	0.01

Discs, large homolog 1 (Drosophila)	DLG1	AI692879	-3.71	0.01	-2.12	0.01
Dynein, axonemal, heavy chain 1	DNAH1	AI004779	-3.42	0.01	-2.2	0.01
Ectonucleoside triphosphate diphosphohydrolase 5	ENTPD5	NM_001249	-3.69	0.01	-3.39	0.01
Electron-transferring-flavoprotein dehydrogenase	ETFDH	S69232	-2.1	0.01	-2.1	0.01
endo-beta-N-acetylglucosaminidase	-	NM_022759	-3.51	0.01	-2.25	0.01
Endothelin 1	EDN1	NM_001955	-2.56	0.01	-2.38	0.01
Endothelin 3	EDN3	NM_000114	-2.59	0.01	-2.59	0.01
enolase superfamily member 1	-	AF305057	-3.4	0.01	-2.14	0.01
Enoyl Coenzyme A hydratase domain containing 2	ECHDC2	AI903313	-4.21	0.01	-2.37	0.01
Ephrin-B2	EFNB2	U16797	-3.01	0.01	-2.41	0.01
Epoxide hydrolase 2, cytoplasmic	EPHX2	AF233336	-2.08	0.01	-2.21	0.01
erythrocyte membrane protein band 4.1 like 4B	-	NM_024823	-3.6	0.01	-2.46	0.01
Ets homologous factor	EHF	AA565141	-2.33	0.01	-2.77	0.01
Ewing sarcoma breakpoint region 1	EWSR1	AW089574	-3.05	0.01	-2.04	0.01
Exocyst complex component 3	EXOC3	BF528646	-2.84	0.02	-3.77	0.01
Exophilin 5	EXPH5	AL050204	-3.46	0.01	-2.8	0.01
Ezrin	EZR	AF199015	-2.45	0.01	-2.21	0.01
family with sequence similarity 195, member A	-	NM_032371	-2.25	0.01	-2	0.01
Family with sequence similarity 55, member D	FAM55D	NM_017678	-7.84	0.01	-4.32	0.01
Family with sequence similarity 82, member A1	FAM82A1	AI261321	-2.04	0.01	-2.09	0.01
F-box protein 32	-	BF244402	-4.14	0.01	-2.47	0.01
Fc fragment of IgG, receptor, transporter, alpha	FCGRT	NM_004107	-2.67	0.01	-2.47	0.01
FERM domain containing 1	FRMD1	NM_024919	-2.28	0.01	-2.03	0.01
FERM, RhoGEF (ARHGEF) and	FARP1	AW102941	-2.85	0.01	-2.02	0.01

pleckstrin domain protein 1 (chondrocyte-derived)						
Fibroblast growth factor 9 (glia-activating factor)	FGF9	NM_002010	-2.48	0.01	-2.12	0.01
Fibroblast growth factor receptor 2	FGFR2	M80634	-2.35	0.01	-2.52	0.01
Fibroblast growth factor receptor 3	FGFR3	NM_000142	-3.45	0.01	-2.69	0.01
Filamin binding LIM protein 1	FBLIM1	AF459643	-2.54	0.01	-2.44	0.01
Flavin containing monooxygenase 5	FMO5	AI935710	-2.13	0.01	-2.17	0.01
Forkhead box A1	FOXA1	AI693336	-2.68	0.01	-2.36	0.01
FRY-like	FRYL	AL833444	-2.88	0.01	-2.07	0.01
Full length insert cDNA clone YR74F10	-	AF085948	-3.61	0.01	-2.22	0.01
Full length insert cDNA clone ZB94A08	-	W04694	-3.45	0.01	-2.02	0.01
Full length insert cDNA clone ZD60E09	-	W76548	-4.07	0.01	-2.28	0.01
Full length insert cDNA YH99G08	-	AA889954	-3.26	0.01	-2.43	0.01
Full length insert cDNA YN63H06	-	AI887306	-3.09	0.01	-2.13	0.01
FXYD domain containing ion transport regulator 3	FXYD3	NM_005971	-3.46	0.01	-2.92	0.01
Glucosaminyl (N-acetyl) transferase 2, I-branching enzyme (I blood group)	GCNT2	BF059748	-2.46	0.01	-2.62	0.01
Glutaminase	GLS	NM_014905	-2.04	0.01	-2	0.01
Golgi autoantigen, golgin subfamily a, 2-like 1	GOLGA2L1	AL359652	-3.7	0.01	-2.46	0.01
Guanine nucleotide binding protein (G protein), alpha 11 (Gq class)	GNA11	NM_002067	-3.19	0.01	-2.9	0.01
Guanylate cyclase activator 1B (retina)	GUCA1B	NM_002098	-9.88	0.01	-8.61	0.01
Guanylate cyclase activator 2B (uroguanylin)	GUCA2B	NM_007102	-8.39	0.01	-6.4	0.01
Haloacid dehalogenase-like hydrolase domain containing 3	HDHD3	NM_031219	-2.32	0.01	-2.04	0.01

Heat shock 27kDa protein 3	HSPB3	NM_006308	-3.21	0.02	-4.05	0.01
Heat shock factor binding protein 1-like	LOC440498	AK001829	-3.26	0.01	-2.38	0.01
HECT domain containing 1	HECTD1	BE243270	-3.23	0.01	-2.21	0.01
heterogeneous nuclear ribonucleoprotein C (C1/C2)	-	AA889628	-3.37	0.01	-2.26	0.01
Homeobox A5	HOXA5	NM_019102	-2.96	0.01	-2.46	0.01
Homeobox B5	HOXB5	NM_002147	-2.53	0.01	-2.02	0.01
Homo sapiens cDNA FLJ20670 fis, clone KAIA4743.	-	AK000677	-5.29	0.01	-3.13	0.01
Homo sapiens cDNA FLJ35505 fis, clone SMINT2009363.	-	AK092824	-5.97	0.01	-3.17	0.01
Homo sapiens cDNA FLJ36460 fis, clone THYMU2014801.	-	AA524609	-4.12	0.01	-2.47	0.01
Homo sapiens cDNA: FLJ21718 fis, clone COL10356.	-	AK025371	-2.39	0.01	-2.29	0.01
Homo sapiens full length insert cDNA clone YB34H05	-	AW341225	-4.14	0.01	-2.38	0.01
Homo sapiens hqp0376 protein mRNA, complete cds.	-	AF078844	-2.5	0.01	-2.31	0.01
Homo sapiens hypothetical protein FLJ23556 (FLJ23556), mRNA.	-	NM_024880	-6.1	0.01	-3.09	0.01
Homo sapiens NAG13 (NAG13) mRNA, complete cds.	-	AF194537	-3.33	0.01	-2.22	0.01
Homo sapiens PRO2221 mRNA, complete cds.	-	AF116695	-5.02	0.01	-2.53	0.01
Homo sapiens, clone IMAGE:3959993, mRNA	-	BQ219651	-3.52	0.01	-2.11	0.01
Homo sapiens, clone IMAGE:4106389, mRNA	-	BC016780	-4.63	0.04	-3.14	0.02
Homo sapiens, clone IMAGE:4275461,	-	BC026299	-5.15	0.01	-2.9	0.01

mRNA						
Homo sapiens, clone IMAGE:4722681, mRNA	-	BC035958	-3.73	0.01	-2.19	0.01
Hydroxyacyl-Coenzyme A dehydrogenase/3-ketoacyl-Coenzyme A thiolase/enoyl-Coenzyme A hydratase (trifunctional protein), alpha	HADHA	BG472176	-2.39	0.01	-2.18	0.01
Hydroxy-delta-5-steroid dehydrogenase, 3 beta- and steroid delta-isomerase 1	HSD3B1	NM_000862	-2.05	0.01	-2.76	0.01
hydroxy-delta-5-steroid dehydrogenase, 3 beta- and steroid delta-isomerase 1 /// hydroxy-delta-5-steroid dehydrogenase, 3 beta- and steroid delta-isomerase 2 /// 3-beta-hydroxysteroid dehydrogenase, tissue-type heart /// similar to 3 beta-hydroxyster	-	AL359553	-2.19	0.01	-2.06	0.01
Hydroxy-delta-5-steroid dehydrogenase, 3 beta- and steroid delta-isomerase 2	HSD3B2	NM_000198	-9.47	0.02	-15.15	0.01
Hydroxysteroid (11-beta) dehydrogenase 2	HSD11B2	NM_000196	-2.46	0.02	-2.25	0.01
Hydroxysteroid (17-beta) dehydrogenase 3	HSD17B3	NM_000197	-2.8	0.01	-2.12	0.01
Hypothetical gene supported by AK026416	FLJ22763	AI650260	-2.06	0.01	-2.54	0.01
Hypothetical gene supported by AK126569	FLJ44606	BE674694	-3.55	0.01	-2.38	0.01
Hypothetical gene supported by AK128346	LOC440993	BM914560	-5.48	0.01	-3.16	0.01

Hypothetical gene supported by BC032913; BC048425	LOC389023	AI499651	-3.56	0.01	-3.6	0.01
Hypothetical LOC100131541	UNQ6228	AL832308	-4.12	0.01	-2.33	0.01
hypothetical LOC100272216	-	AU158490	-4.35	0.01	-2.16	0.02
Hypothetical LOC150538	FLJ32063	BC035902	-4.65	0.01	-3.6	0.01
hypothetical LOC150538	-	NM_153031	-2.21	0.02	-2.2	0.01
hypothetical LOC151009	-	AI699847	-2.42	0.01	-2.02	0.01
Hypothetical LOC339290	LOC339290	H49382	-2.59	0.01	-2.01	0.01
hypothetical LOC440993	-	CA418406	-4.73	0.01	-3.12	0.01
Hypothetical LOC641467	LOC641467	BE670165	-2.91	0.01	-2.04	0.01
Hypothetical LOC643187	LOC643187	BC016022	-4.8	0.01	-2.77	0.01
Hypothetical protein FLJ20674	FLJ20674	BG547864	-3.14	0.01	-2.75	0.01
Hypothetical protein LOC100128893	LOC100128893	N63706	-3.11	0.01	-2.6	0.01
Hypothetical protein LOC129293	LOC129293	AA005361	-3.18	0.01	-2.4	0.01
Hypothetical protein LOC151878	LOC151878	AW009761	-4.32	0.01	-2.49	0.01
Hypothetical protein LOC283177	LOC283177	AI743605	-3.24	0.01	-2.57	0.01
Hypothetical protein LOC285758	LOC285758	BC037331	-3.72	0.01	-2.69	0.01
Immunoglobulin superfamily, member 9	IGSF9	AB037776	-3.52	0.01	-2.88	0.01
InaD-like (Drosophila)	INADL	AJ001306	-3.35	0.01	-2.2	0.01
Inositol 1,4,5-trisphosphate 3-kinase A	ITPKA	NM_002220	-2.88	0.01	-2.41	0.01
Inositol hexakisphosphate kinase 2	IP6K2	BC004469	-3.17	0.01	-2.34	0.01
Inositol polyphosphate-5-phosphatase J	INPP5J	AI935720	-2.51	0.01	-2.21	0.01
insulin-like growth factor 1 receptor	-	H05812	-2.71	0.01	-2.12	0.01
Keratin 12	KRT12	NM_000223	-4.34	0.01	-3.69	0.01
KIAA0574 protein	KIAA0574	AB011146	-2.45	0.01	-2.34	0.01
Kinesin family member C2	KIFC2	BF515031	-3.19	0.01	-2.09	0.01

KN motif and ankyrin repeat domains 4	KANK4	AA456955	-3.49	0.01	-2.36	0.01
Kruppel-like factor 5 (intestinal)	KLF5	AB030824	-2.36	0.01	-2.18	0.01
Lactate dehydrogenase D	LDHD	AI623801	-2.41	0.01	-2.13	0.01
Laminin, alpha 1	LAMA1	AI990816	-3.84	0.01	-3.07	0.01
lectin, galactoside-binding, soluble, 3	-	AW085690	-3.64	0.01	-2.56	0.01
Leucine rich repeat (in FLII) interacting protein 1	LRRFIP1	AA262583	-3.75	0.01	-2.1	0.01
Leucine rich repeat (in FLII) interacting protein 2	LRRFIP2	AK025207	-3.94	0.01	-2.12	0.01
leucine rich repeat containing 1	-	NM_025168	-2.7	0.01	-2.09	0.01
Leucine rich repeat neuronal 2	LRRN2	NM_006338	-2.41	0.01	-2.35	0.01
Leucine zipper-EF-hand containing transmembrane protein 1	LETM1	AI359368	-2.18	0.01	-2.05	0.01
LIM domain 7	LMO7	AA702962	-3.35	0.01	-2.27	0.01
LIM domain containing preferred translocation partner in lipoma	LPP	AI079544	-5.4	0.01	-2.7	0.01
Macrophage stimulating 1 (hepatocyte growth factor-like)	MST1	U28055	-4.26	0.02	-2.92	0.01
Macrophage stimulating, pseudogene 9	MSTP9	AA911235	-4.7	0.01	-2.9	0.01
Malic enzyme 3, NADP(+)-dependent, mitochondrial	ME3	NM_006680	-3.98	0.01	-2.88	0.01
MAM domain containing 4	MAMDC4	AI978986	-3.25	0.01	-2.1	0.01
Mannosyl (alpha-1,3-)-glycoprotein beta-1,4-N-acetylglucosaminyltransferase, isozyme B	MGAT4B	NM_014275	-2.34	0.01	-2.12	0.01
MARVEL domain containing 3	MARVELD3	BC005052	-4.37	0.01	-3.13	0.01
Matrix metallopeptidase 28	MMP28	NM_024302	-2.31	0.01	-2.13	0.01
Membrane associated guanylate kinase,	MAGI1	NM_004742	-2.78	0.01	-2.06	0.01

WW and PDZ domain containing 1						
Membrane-spanning 4-domains, subfamily A, member 12	MS4A12	NM_017716	-9.47	0.02	-5.38	0.01
Meprin A, beta	MEP1B	NM_005925	-6.37	0.01	-8.09	0.01
Metallothionein 1F	MT1F	BF246115	-3.11	0.01	-2.78	0.01
Metallothionein 1G	MT1G	NM_005950	-2.52	0.01	-2.22	0.01
Metallothionein 1M	MT1M	R06655	-7.19	0.01	-4.64	0.01
metastasis associated lung adenocarcinoma transcript 1 (non-protein coding)	-	AF001540	-33.38	0.01	-8.83	0.01
Methyltransferase like 7B	METTL7B	AI827972	-2.38	0.02	-2.66	0.01
Microsomal glutathione S-transferase 1	MGST1	AV705233	-2.85	0.01	-2.23	0.01
Microtubule associated serine/threonine kinase 2	MAST2	BE786598	-2.17	0.01	-2.02	0.01
Microtubule associated serine/threonine kinase family member 4	MAST4	AW971134	-2.98	0.01	-2.13	0.01
Mitochondrial tumor suppressor 1	MTUS1	AI028661	-3.53	0.01	-2.13	0.01
Mitogen-activated protein kinase kinase kinase 13	MAP3K13	BC026249	-5.08	0.01	-2.8	0.01
Molybdenum cofactor synthesis 1	MOCS1	AL583528	-2.39	0.01	-2.5	0.01
Monoamine oxidase A	MAOA	AA923354	-2.5	0.01	-2.45	0.01
Monoglyceride lipase	MGLL	BC006230	-2.06	0.01	-2.05	0.01
MRNA full length insert cDNA clone EUROIMAGE 85905	-	AL080280	-3.51	0.01	-2.21	0.02
MRNA; cDNA DKFZp434F0472 (from clone DKFZp434F0472)	-	AL137331	-2.73	0.01	-2.06	0.01
MRNA; cDNA DKFZp434M0835 (from clone DKFZp434M0835)	-	AL137325	-3.68	0.01	-2.41	0.01
MRNA; cDNA DKFZp564C142 (from clone DKFZp564C142)	-	AL049979	-4.32	0.01	-2.21	0.01

MRNA; cDNA DKFZp586G081 (from clone DKFZp586G081)	-	AL050129	-3.07	0.01	-2.02	0.01
MRNA; cDNA DKFZp667C0715 (from clone DKFZp667C0715)	-	AL713714	-3.08	0.04	-2.28	0.03
MRNA; cDNA DKFZp761E11121 (from clone DKFZp761E11121)	-	AL137309	-4.07	0.01	-2.7	0.01
MRNA; cDNA DKFZp761L149 (from clone DKFZp761L149)	-	AL390180	-5.97	0.01	-2.96	0.01
MRNA; cDNA DKFZp761M2223 (from clone DKFZp761M2223)	-	AL162055	-3.12	0.01	-2.1	0.01
Mucin 20, cell surface associated	MUC20	AA132448	-2.83	0.01	-2.07	0.01
Mucin-like protocadherin	MUPCDH	NM_021924	-2.99	0.01	-2.71	0.01
Mucolipin 2	MCOLN2	AY083533	-4.31	0.01	-2.94	0.01
Muscleblind-like (Drosophila)	MBNL1	AL562860	-3.06	0.01	-2.02	0.01
Myelodysplasia syndrome 1	MDS1	NM_004991	-2.9	0.01	-2.1	0.01
Myosin IA	MYO1A	AF009961	-2.3	0.01	-2.19	0.01
Myosin ID	MYO1D	AA621962	-2.25	0.01	-2.4	0.01
myosin X	-	AI561354	-3.24	0.01	-2.33	0.01
Myosin XVB pseudogene	MYO15B	AI825877	-2.98	0.01	-2.15	0.01
myosin XVB pseudogene	-	NM_024957	-2.59	0.01	-2.01	0.01
Myosin, heavy chain 14	MYH14	AW081820	-2.75	0.01	-2.07	0.01
Myosin, light chain 5, regulatory	MYL5	NM_002477	-3.15	0.01	-2.49	0.01
Myotilin	MYOT	NM_006790	-2.71	0.01	-2.18	0.01
Myotubularin related protein 11	MTMR11	AK097000	-2.62	0.01	-2.32	0.01
N-acyl phosphatidylethanol amine phospholipase D	NAPEPLD	AK000801	-3.16	0.01	-2.22	0.01
N-acylethanolamine acid amidase	NAAA	AI436803	-2.29	0.01	-2.12	0.01
NADH dehydrogenase, subunit 6 (complex I)	-	NM_173714	-3.97	0.01	-2.11	0.01
NDRG family	NDRG2	NM_016250	-2.35	0.01	-2.05	0.01

member 2						
Neighbor of BRCA1 gene 1	NBR1	BC012591	-3.18	0.01	-2.12	0.01
Neural precursor cell expressed, developmentally down-regulated 4-like	NEDD4L	AV700008	-4.02	0.01	-2.41	0.01
Neuralized homolog 1B (Drosophila)	NEURL1B	AK026748	-2.57	0.01	-2.49	0.01
Neurexin 1	NRXN1	BF509242	-2.53	0.04	-2.13	0.04
Neurobeachin-like 1	NBEAL1	AB053318	-3.09	0.01	-2.28	0.01
Neuroblastoma breakpoint family, member 1	NBPF1	AI634549	-5.88	0.01	-3.31	0.01
Neuroblastoma breakpoint family, member 10	NBPF10	BE732345	-2.94	0.01	-2.07	0.01
Nipped-B homolog (Drosophila)	NIPBL	AW272262	-3.33	0.01	-2.23	0.01
NOL1/NOP2/Sun domain family, member 4	NSUN4	NM_001441	-2.49	0.01	-2.01	0.01
NOL1/NOP2/Sun domain family, member 5B	NSUN5B	AI768378	-4.15	0.01	-2.18	0.01
Non-protein coding RNA 84	NCRNA00084	AU134977	-4.72	0.01	-2.57	0.01
Nuclear pore complex interacting protein-like 3	NPIPL3	BG256504	-4.04	0.01	-2.34	0.01
Nuclear receptor subfamily 1, group I, member 2	NR1I2	NM_003889	-2.76	0.01	-2.64	0.01
Olfactory receptor, family 2, subfamily H, member 1	OR2H1	AJ459849	-2.33	0.01	-2.09	0.01
Organic solute transporter beta	OSTbeta	AI479168	-2.66	0.01	-2.98	0.01
OTU domain containing 7B	OTUD7B	AW022496	-3.13	0.01	-2.01	0.01
Oxysterol binding protein-like 1A	OSBPL1A	W19983	-2.04	0.01	-2.23	0.01
Paired immunoglobin-like type 2 receptor beta	PILRB	AV762653	-6.12	0.01	-2.78	0.01
Pancreatic lipase-related protein 2	PNLIPRP2	BC005989	-8.15	0.01	-4.57	0.01
PDZ domain containing 3	PDZD3	NM_024791	-3.82	0.01	-2.84	0.01
peptidyl arginine deiminase, type II	-	AL049569	-7.61	0.01	-5.77	0.01
Peptidylprolyl isomerase D	PPID	BF431902	-2.55	0.01	-2.79	0.01

Per1-like domain containing 1	PERLD1	AI703342	-2.53	0.01	-2.02	0.01
Peroxisomal membrane protein 2, 22kDa	PXMP2	NM_018663	-2.44	0.01	-2.33	0.01
Peroxisome proliferator-activated receptor gamma, coactivator 1 alpha	PPARGC 1A	NM_013261	-2.03	0.01	-2.26	0.01
PH domain and leucine rich repeat protein phosphatase-like	PHLPPL	AB023148	-4.29	0.01	-3.99	0.01
Phosphatidylinositol glycan anchor biosynthesis, class Z	PIGZ	NM_025163	-3.69	0.01	-3.14	0.01
Phosphodiesterase 6A, cGMP-specific, rod, alpha	PDE6A	NM_000440	-4.47	0.01	-4.39	0.01
Phosphodiesterase 8A	PDE8A	NM_002605	-2.55	0.01	-2.36	0.01
Phosphoenolpyruvate carboxykinase 1 (soluble)	PCK1	NM_002591	-22.88	0.01	-15.38	0.01
Phospholipase A2, group XIIB	PLA2G12 B	BF939574	-2.8	0.01	-3.73	0.01
Phospholipase C, epsilon 1	PLCE1	NM_016341	-2.89	0.01	-2.52	0.01
Phospholipase D1, phosphatidylcholine-specific	PLD1	AA132961	-2.77	0.01	-2.09	0.03
Phospholipase inhibitor	LOC6466 27	CA424969	-3.28	0.01	-3.11	0.01
Phosphomannomutase 1	PMM1	NM_002676	-2.34	0.01	-2.1	0.01
Placenta-specific 2 (non-protein coding)	PLAC2	AI743780	-2.48	0.01	-2.13	0.01
Pleckstrin homology domain containing, family G (with RhoGef domain) member 6	PLEKHG6	NM_018173	-2.89	0.01	-2.88	0.01
Pleckstrin homology domain containing, family H (with MyTH4 domain) member 1	PLEKHH1	AB033026	-2.65	0.01	-2.03	0.01
Plexin A2	PLXNA2	AI688418	-2.36	0.01	-2.01	0.01
Poly(rC) binding protein 2	PCBP2	AW103422	-5.02	0.01	-2.65	0.01
Polymerase (RNA) II (DNA directed) polypeptide J4, pseudogene	POLR2J4	BQ613856	-2.91	0.01	-2.03	0.01
postmeiotic	-	AK091784	-3.54	0.01	-2.07	0.01

segregation increased 2-like 5-like /// postmeiotic segregation increased 2-like 1 pseudogene /// postmeiotic segregation increased 2-like 2 pseudogene						
PP12104	LOC643008	BF478120	-4.5	0.01	-2.58	0.01
Progestin and adipoQ receptor family member V	PAQR5	AI934557	-2.46	0.01	-2.35	0.01
Programmed cell death 4 (neoplastic transformation inhibitor)	PDCD4	AI185160	-2.47	0.01	-2.08	0.01
Proline rich 15	PRR15	AI347918	-2.72	0.01	-2.53	0.01
Proline-rich acidic protein 1	PRAP1	AA502331	-3.99	0.01	-4.65	0.01
Proprotein convertase subtilisin/kexin type 6	PCSK6	AI056815	-5.06	0.01	-3.27	0.01
Proprotein convertase subtilisin/kexin type 7	PCSK7	AK027156	-3.4	0.01	-2.06	0.01
Prostaglandin D2 receptor (DP)	PTGDR	AK026202	-2.85	0.01	-2.12	0.01
Protein kinase, cGMP-dependent, type II	PRKG2	NM_006259	-2.41	0.01	-2.49	0.01
Protein kinase, X-linked, pseudogene 1	PRKXP1	AI983986	-3.95	0.01	-2.04	0.04
Protein phosphatase 1, regulatory (inhibitor) subunit 16A	PPP1R16A	AI742931	-2.32	0.01	-2.01	0.01
Protein tyrosine phosphatase, receptor type, F	PTPRF	AU158443	-5.28	0.01	-3.06	0.01
Proteolipid protein 1	PLP1	BC002665	-2.64	0.02	-2.33	0.01
Protocadherin 21	PCDH21	AI825832	-6.15	0.01	-3.7	0.01
PTPRF interacting protein, binding protein 1 (liprin beta 1)	PPFIBP1	AI962377	-4.02	0.01	-2.24	0.01
Pyruvate dehydrogenase kinase, isozyme 4	PDK4	AL832708	-2.82	0.02	-2.66	0.01
RAB2A, member	RAB2A	AA535244	-3	0.01	-2.05	0.01

RAS oncogene family						
RAB40B, member RAS oncogene family	RAB40B	AI344141	-2.51	0.01	-2.01	0.01
RAB6A, member RAS oncogene family	RAB6A	AI805050	-2.09	0.01	-2.32	0.01
Ral GEF with PH domain and SH3 binding motif 1	RALGPS1	NM_014636	-2.62	0.01	-2.19	0.01
Rap guanine nucleotide exchange factor (GEF)-like 1	RAPGEFL1	NM_016339	-3.37	0.01	-2.87	0.01
RAS and EF-hand domain containing	RASEF	NM_152573	-5.94	0.01	-3.02	0.01
Ras association (RalGDS/AF-6) domain family member 6	-	AU157324	-3.39	0.01	-2.14	0.01
Ras homolog gene family, member U	RHOU	AB051826	-2.12	0.01	-2.37	0.01
Retinol dehydrogenase 5 (11-cis/9-cis)	RDH5	AI887702	-2.85	0.01	-2.02	0.01
Retinol saturase (all-trans-retinol 13,14-reductase)	RETSAT	NM_017750	-2.6	0.01	-2.37	0.01
Rho guanine nucleotide exchange factor (GEF) 10-like	-	BC029928	-3.18	0.01	-2.22	0.01
Rho-type GTPase-activating protein RICH2	-	AK023797	-4.04	0.01	-2.82	0.01
Ribonucleoprotein, PTB-binding 2	RAVER2	AL359613	-2.7	0.01	-2.41	0.01
Ring finger protein 207	RNF207	AK056658	-3.8	0.01	-2.17	0.01
RNA binding motif protein 25	RBM25	AA580691	-3.75	0.01	-2.12	0.01
RNA binding motif protein 39	RBM39	BE466173	-4.71	0.01	-2.46	0.01
RRN3 RNA polymerase I transcription factor homolog (S. cerevisiae) pseudogene	LOC100131998	AI344253	-2.87	0.01	-2	0.01
RUN domain containing 3B	RUNDC3B	AI825798	-3.2	0.01	-3.63	0.01
S-adenosylhomocysteine hydrolase-like 2	AHCYL2	AB020635	-2.22	0.01	-2.25	0.01
SATB homeobox 2	-	AK025127	-10.24	0.01	-5.25	0.01
Scinderin	SCIN	AF276507	-2.76	0.01	-3.46	0.01

Selenium binding protein 1	SELENBP1	NM_003944	-5.1	0.01	-3.92	0.01
Sema domain, seven thrombospondin repeats (type 1 and type 1-like), transmembrane domain (TM) and short cytoplasmic domain, (	SEMA5A	NM_003966	-2.91	0.01	-2.35	0.01
Sema domain, transmembrane domain (TM), and cytoplasmic domain, (semaphorin) 6A	SEMA6A	AB002438	-6.56	0.01	-4.13	0.01
Sema domain, transmembrane domain (TM), and cytoplasmic domain, (semaphorin) 6D	SEMA6D	NM_024966	-3.73	0.01	-2.79	0.01
Serine/arginine repetitive matrix 2	SRRM2	AI655799	-3.59	0.01	-2.07	0.01
Serine/threonine kinase 38	STK38	BU617137	-2.87	0.01	-2.1	0.01
Serum/glucocorticoid regulated kinase 2	SGK2	AI631895	-4.76	0.01	-4.35	0.01
SH3 domain containing ring finger 2	SH3RF2	AW082633	-2.63	0.01	-2.36	0.01
Shroom family member 1	SHROOM1	AA588854	-2.71	0.01	-2.07	0.01
Signal transducing adaptor family member 2	STAP2	BC000795	-2.92	0.01	-2.61	0.01
similar to FAM133B protein	-	AU120130	-4.85	0.01	-2.55	0.01
Similar to hCG1983765	LOC100133019	BC029255	-4.99	0.01	-2.86	0.01
Similar to KIAA1839 protein	LOC641705	BF116157	-3.24	0.01	-2.17	0.01
Similar to p40	LOC728678	AK000115	-4.79	0.01	-2.41	0.02
Similar to Vesicle-fusing ATPase (Vesicular-fusion protein NSF) (N-ethylmaleimide sensitive fusion protein) (NEM-sensitive fu	LOC728806	AK025172	-2.55	0.01	-2.13	0.01
Small EDRK-rich factor 1A (telomeric)	SERF1A	AF073518	-2.4	0.01	-2.2	0.01
SMG1 homolog, phosphatidylinositol	LOC641298	BC042832	-3.19	0.01	-2.03	0.02

3-kinase-related kinase pseudogene						
Sodium channel, nonvoltage-gated 1, beta	SCNN1B	NM_000336	-2.71	0.02	-2.19	0.01
Solute carrier family 1 (glutamate transporter), member 7	SLC1A7	N39099	-2.64	0.01	-2.52	0.01
Solute carrier family 13 (sodium-dependent dicarboxylate transporter), member 2	SLC13A2	AW136060	-2.6	0.01	-2.27	0.01
Solute carrier family 16, member 1 (monocarboxylic acid transporter 1)	SLC16A1	BF511091	-3.49	0.01	-3.16	0.01
Solute carrier family 16, member 9 (monocarboxylic acid transporter 9)	SLC16A9	BG401568	-6.67	0.01	-4.65	0.01
Solute carrier family 17 (sodium phosphate), member 4	SLC17A4	NM_005495	-8.08	0.01	-6.66	0.01
Solute carrier family 19, member 3	SLC19A3	NM_025243	-2.76	0.01	-2.74	0.01
Solute carrier family 20 (phosphate transporter), member 1	SLC20A1	NM_005415	-2.51	0.01	-2.41	0.01
Solute carrier family 22 (extraneuronal monoamine transporter), member 3	SLC22A3	AV699746	-3.05	0.01	-2.04	0.01
Solute carrier family 22 (organic cation/carnitine transporter), member 5	SLC22A5	NM_003060	-3.18	0.01	-3.31	0.01
Solute carrier family 22, member 23	SLC22A23	AL512737	-2.8	0.01	-2.39	0.01
Solute carrier family 23 (nucleobase transporters), member 1	SLC23A1	AF170911	-4.56	0.01	-4	0.01
Solute carrier family 23 (nucleobase transporters), member 3	SLC23A3	AI263078	-5.24	0.01	-3.98	0.01
Solute carrier family 25 (mitochondrial	SLC25A23	BG290819	-2.82	0.01	-2.4	0.01

carrier; phosphate carrier), member 23						
Solute carrier family 25, member 34	SLC25A34	AL832282	-3.63	0.01	-3.07	0.01
Solute carrier family 26 (sulfate transporter), member 2	SLC26A2	AI025519	-14.71	0.01	-10.78	0.01
Solute carrier family 26, member 3	SLC26A3	NM_000111	-2.2	0.02	-2.03	0.01
Solute carrier family 26, member 6	SLC26A6	AF288410	-2.93	0.01	-2.15	0.01
Solute carrier family 3 (cystine, dibasic and neutral amino acid transporters, activator of cystine, dibasic and neutral amin	SLC3A1	M95548	-3.25	0.01	-4.76	0.01
Solute carrier family 35, member D2	SLC35D2	AA693722	-3.28	0.01	-2.25	0.01
Solute carrier family 38, member 4	SLC38A4	NM_018018	-7.08	0.02	-10.1	0.01
Solute carrier family 39 (metal ion transporter), member 5	SLC39A5	NM_173596	-2.39	0.01	-2.27	0.01
Solute carrier family 4, sodium bicarbonate cotransporter, member 4	SLC4A4	AF157492	-4.93	0.01	-3.76	0.01
Solute carrier family 9 (sodium/hydrogen exchanger), member 2	SLC9A2	AF073299	-4.43	0.01	-3.47	0.01
Sortilin-related receptor, L(DLR class) A repeats-containing	SORL1	AA290609	-3.35	0.01	-2.23	0.01
Spastic paraplegia 7 (pure and complicated autosomal recessive)	SPG7	BE670386	-3	0.01	-2.33	0.01
Spen homolog, transcriptional regulator (Drosophila)	SPEN	AL524033	-3.97	0.01	-2.34	0.01
Spire homolog 2 (Drosophila)	SPIRE2	AA553722	-3.53	0.01	-2.56	0.01
Splicing factor, arginine/serine-rich 18	SFRS18	N66397	-4.21	0.01	-2.22	0.01
Splicing factor,	SFRS4	AI435248	-3.2	0.01	-2.03	0.01

arginine/serine-rich 4						
Starch binding domain 1	STBD1	NM_003943	-2.03	0.01	-2.11	0.01
Stromal antigen 3-like 1	STAG3L1	NM_018991	-3	0.01	-2	0.01
Structural maintenance of chromosomes flexible hinge domain containing 1	SMCHD1	AA336502	-3.65	0.01	-2.38	0.01
Succinate dehydrogenase complex, subunit A, flavoprotein pseudogene 1	SDHALP1	W90764	-2.97	0.01	-2.14	0.02
Sulfotransferase family, cytosolic, 1A, phenol-preferring, member 1	SULT1A1	NM_001055	-2.22	0.01	-2.18	0.01
Sulfotransferase family, cytosolic, 1A, phenol-preferring, member 2	SULT1A2	NM_001054	-3.1	0.01	-2.7	0.01
Sulfotransferase family, cytosolic, 1A, phenol-preferring, member 3	SULT1A3	U08032	-2.2	0.01	-2.06	0.01
Synaptotagmin-like 2	SYTL2	N21426	-2.9	0.01	-2.21	0.01
TBC1 domain family, member 3F	TBC1D3F	AL136860	-3.24	0.01	-2.02	0.01
Testis expressed 11	TEX11	NM_031276	-2.49	0.01	-2.79	0.01
Tetraspanin 7	TSPAN7	NM_004615	-2.07	0.01	-2.1	0.01
Thioredoxin domain containing 6	TXNDC6	BF942281	-4.54	0.01	-2.44	0.01
Thyroid hormone receptor, beta (erythroblastic leukemia viral (v-erb-a) oncogene homolog 2, avian)	THRB	BG494007	-4.03	0.02	-2.64	0.01
Thyrotropin-releasing hormone degrading enzyme	TRHDE	NM_013381	-2.79	0.03	-3.37	0.01
tight junction protein 2 (zona occludens 2)	-	AK025185	-3.88	0.01	-2.27	0.01
tight junction protein 3 (zona occludens 3)	-	AC005954	-2.73	0.01	-2.32	0.01
Transcribed locus	-	AI090874	-12.84	0.01	-10.55	0.01
Transcribed locus	-	T65568	-12.75	0.01	-6.65	0.01
Transcribed locus	-	T77543	-8.26	0.01	-9.68	0.01

Transcribed locus	-	AI475544	-7.86	0.01	-4.02	0.01
Transcribed locus	-	BF590303	-7.67	0.01	-3.06	0.01
Transcribed locus	-	AI916887	-7.44	0.01	-6.08	0.01
Transcribed locus	-	AW136397	-7.32	0.01	-3.91	0.01
Transcribed locus	-	AW971248	-7.28	0.01	-3.77	0.01
Transcribed locus	-	AW080999	-6.89	0.01	-3.71	0.01
Transcribed locus	-	AA682539	-6.79	0.01	-3.81	0.01
Transcribed locus	-	AA706922	-6.69	0.01	-3.81	0.01
Transcribed locus	-	AW117547	-6.46	0.01	-3.41	0.01
Transcribed locus	-	AA598661	-6.18	0.01	-3.27	0.01
Transcribed locus	-	AI467945	-6.11	0.01	-2.82	0.01
Transcribed locus	-	AW016812	-5.58	0.01	-2.6	0.02
Transcribed locus	-	AW972359	-5.55	0.01	-2.63	0.01
Transcribed locus	-	BF509781	-4.89	0.01	-2.22	0.02
Transcribed locus	-	AW971198	-4.83	0.01	-2.93	0.01
Transcribed locus	-	AW976631	-4.73	0.01	-3.65	0.01
Transcribed locus	-	AI632567	-4.69	0.01	-2.76	0.01
Transcribed locus	-	AW452823	-4.64	0.01	-2.86	0.01
Transcribed locus	-	BF476152	-4.59	0.01	-2.44	0.01
Transcribed locus	-	AA026388	-4.58	0.01	-2.49	0.01
Transcribed locus	-	AW975021	-4.52	0.01	-2.56	0.01
Transcribed locus	-	AI610347	-4.51	0.01	-2.72	0.01
Transcribed locus	-	BF358386	-4.51	0.01	-2.61	0.01
Transcribed locus	-	AI424825	-4.43	0.01	-2.87	0.01
Transcribed locus	-	BC025999	-4.4	0.01	-2.1	0.02
Transcribed locus	-	AI457588	-4.39	0.01	-3.22	0.01
Transcribed locus	-	AW292329	-4.38	0.01	-2.55	0.01
Transcribed locus	-	AW051321	-4.36	0.01	-2.84	0.01
Transcribed locus	-	AL833742	-4.25	0.01	-2.34	0.02
Transcribed locus	-	BF801735	-4.22	0.01	-2.55	0.01
Transcribed locus	-	BE843544	-4.18	0.01	-2.66	0.01
Transcribed locus	-	AI800470	-4.16	0.01	-2.49	0.02
Transcribed locus	-	N55072	-4.14	0.01	-2.64	0.01
Transcribed locus	-	AI936859	-4.1	0.01	-2.52	0.01
Transcribed locus	-	AI768144	-4.07	0.01	-2.26	0.01
Transcribed locus	-	BC014363	-4.07	0.01	-2.2	0.01
Transcribed locus	-	BF725383	-4.07	0.01	-2.05	0.02
Transcribed locus	-	AI912194	-4.06	0.01	-2.5	0.01
Transcribed locus	-	AA649070	-4.03	0.01	-2.3	0.01
Transcribed locus	-	BF508786	-4.03	0.01	-2.44	0.01
Transcribed locus	-	AI018256	-4.01	0.01	-2.25	0.01
Transcribed locus	-	AW451711	-3.98	0.01	-2.37	0.01
Transcribed locus	-	AA702415	-3.95	0.01	-2.75	0.01
Transcribed locus	-	AI697540	-3.92	0.01	-2.02	0.03
Transcribed locus	-	AW129056	-3.88	0.01	-2.13	0.01
Transcribed locus	-	AW474960	-3.84	0.01	-2.55	0.01
Transcribed locus	-	AA404501	-3.83	0.01	-2.4	0.01
Transcribed locus	-	AW452419	-3.83	0.01	-2.32	0.01
Transcribed locus	-	BF676361	-3.82	0.01	-2.01	0.02

Transcribed locus	-	BF843343	-3.82	0.01	-2.52	0.01
Transcribed locus	-	BE501559	-3.81	0.01	-2.41	0.01
Transcribed locus	-	AA921841	-3.77	0.01	-2.2	0.01
Transcribed locus	-	BG255923	-3.68	0.01	-2.24	0.01
Transcribed locus	-	AI907083	-3.67	0.01	-2.04	0.01
Transcribed locus	-	AA654772	-3.64	0.01	-2.01	0.02
Transcribed locus	-	AI268231	-3.64	0.01	-2.2	0.02
Transcribed locus	-	AW195351	-3.6	0.01	-2.32	0.01
Transcribed locus	-	AI431345	-3.55	0.01	-2.28	0.01
Transcribed locus	-	AI797677	-3.54	0.01	-2.18	0.01
Transcribed locus	-	AA468422	-3.52	0.01	-2.39	0.01
Transcribed locus	-	AI248760	-3.52	0.01	-2.43	0.01
Transcribed locus	-	AI312527	-3.52	0.01	-2.03	0.01
Transcribed locus	-	AI475033	-3.5	0.01	-2.06	0.03
Transcribed locus	-	AA665058	-3.48	0.01	-2.2	0.01
Transcribed locus	-	AI420611	-3.48	0.01	-2.07	0.01
Transcribed locus	-	AI393727	-3.41	0.01	-2.14	0.01
Transcribed locus	-	AA565051	-3.38	0.01	-2.07	0.01
Transcribed locus	-	AW090199	-3.33	0.01	-2.21	0.01
Transcribed locus	-	BE156563	-3.28	0.01	-2.04	0.01
Transcribed locus	-	AV694053	-3.24	0.02	-3.92	0.01
Transcribed locus	-	AL832146	-3.22	0.01	-2.02	0.01
Transcribed locus	-	N74444	-3.19	0.01	-2.03	0.01
Transcribed locus	-	AL833045	-3.18	0.01	-2.31	0.01
Transcribed locus	-	AI373107	-3.17	0.01	-2.03	0.01
Transcribed locus	-	BF433975	-3.17	0.01	-2.06	0.01
Transcribed locus	-	AL047522	-3.11	0.01	-2.18	0.01
Transcribed locus	-	AU144462	-3.09	0.01	-2.06	0.01
Transcribed locus	-	BG054682	-3.09	0.01	-2.06	0.01
Transcribed locus	-	AA765786	-3.07	0.01	-2.02	0.01
Transcribed locus	-	AA033699	-3.02	0.01	-2.01	0.01
Transcribed locus	-	AI654130	-2.93	0.01	-2.01	0.01
Transcribed locus	-	AI264135	-2.89	0.01	-2.13	0.01
Transcribed locus	-	AW265065	-2.89	0.01	-2.18	0.01
Transcribed locus	-	AI587332	-2.81	0.03	-2.25	0.02
Transcribed locus	-	AV693734	-2.8	0.01	-2.21	0.01
Transcribed locus	-	BE501878	-2.8	0.01	-2.29	0.01
Transcribed locus	-	BE671532	-2.79	0.01	-2.11	0.01
Transcribed locus	-	C14898	-2.71	0.01	-2.06	0.01
Transcribed locus	-	AA456945	-2.68	0.01	-2.07	0.01
Transcribed locus	-	AW014327	-2.54	0.01	-2.18	0.01
Transcribed locus	-	AI198547	-2.52	0.01	-2.2	0.01
Transcribed locus	-	AI004009	-2.51	0.01	-2.55	0.01
Transcribed locus	-	AI394574	-2.49	0.01	-2.1	0.01
Transcribed locus	-	AI738422	-2.49	0.01	-2.1	0.01
Transcribed locus	-	AW138815	-2.42	0.01	-2.39	0.01
Transcribed locus	-	BG290650	-2.27	0.01	-2.12	0.01
Transcribed locus	-	W73272	-2.22	0.01	-2.09	0.01
Transcribed locus	-	AW451432	-2.17	0.01	-2.26	0.01

Transcribed locus	-	BE550153	-2.06	0.01	-2.09	0.01
Transcribed locus, moderately similar to NP_598502.1 SNF related kinase [Mus musculus]	-	BF434212	-3.52	0.01	-2.66	0.01
Transcribed locus, moderately similar to XP_001719123.1 PREDICTED: hypothetical protein, partial [Homo sapiens]	-	AV684285	-2.85	0.01	-2.09	0.01
Transcribed locus, strongly similar to NP_003874.2 histone deacetylase 3 [Homo sapiens]	-	AL037917	-2.75	0.01	-2.01	0.01
Transcribed locus, strongly similar to NP_008916.2 nuclear pore complex interacting protein [Homo sapiens]	-	AA308853	-3.3	0.01	-2.05	0.01
Transcribed locus, weakly similar to NP_001006512.1 NEDD8-conjugating enzyme [Gallus gallus]	-	AW137053	-2.62	0.01	-2.38	0.01
Transcribed locus, weakly similar to XP_001091208.1 PREDICTED: hypothetical protein [Macaca mulatta]	-	T93073	-3.52	0.01	-2.36	0.01
Transcription elongation factor A (SII), 3	TCEA3	AI675780	-2.77	0.01	-2.42	0.01
Transcription factor CP2-like 1	TFCP2L1	AI928242	-2.59	0.01	-2.12	0.01
Transient receptor potential cation channel, subfamily M, member 4	TRPM4	NM_017636	-2.69	0.01	-2.18	0.01
Transient receptor potential cation channel, subfamily M, member 6	TRPM6	AF350881	-14.44	0.01	-8.2	0.01
Transient receptor potential cation channel, subfamily V, member 1	TRPV1	AF196175	-3.19	0.01	-2.29	0.01
Transmembrane	TMEM178	AA058832	-3.13	0.01	-2.26	0.01

protein 178						
transmembrane protein 184A	-	BQ277407	-4.1	0.01	-2.64	0.01
Transmembrane protein 20	TMEM20	R56424	-2.19	0.01	-2.37	0.01
Transmembrane protein 37	TMEM37	N93191	-2.44	0.01	-2.49	0.01
Transmembrane protein 63C	TMEM63 C	AA928939	-2.54	0.01	-2.19	0.01
Transmembrane protein 72	TMEM72	BC029135	-2.79	0.04	-2.59	0.01
Triple functional domain (PTPRF interacting)	TRIO	N21108	-3.59	0.01	-2.04	0.01
Tubulin tyrosine ligase-like family, member 6	TTLL6	AI698574	-3.26	0.01	-2.29	0.01
Tubulin, alpha-like 3	TUBAL3	NM_024803	-2.22	0.01	-2.68	0.01
Tubulointerstitial nephritis antigen	TINAG	AF195116	-2.2	0.01	-2.39	0.01
tumor protein p53 inducible nuclear protein 2	-	AL109824	-2.07	0.01	-2.09	0.01
Ubiquinol-cytochrome c reductase core protein II	UQCRC2	AI961429	-2.98	0.01	-2.14	0.01
ubiquitin specific peptidase 34	-	AL050376	-4.22	0.01	-2.33	0.01
UDP glucuronosyltransferase 1 family, polypeptide A1	UGT1A1	NM_019076	-2.25	0.01	-2.84	0.01
UDP-glucose pyrophosphorylase 2	UGP2	U00954	-4.74	0.01	-2.74	0.01
Usher syndrome 1C (autosomal recessive, severe)	-	NM_025034	-4.6	0.01	-2.79	0.01
Vacuolar protein sorting 13 homolog B (yeast)	VPS13B	BE048857	-3.57	0.01	-2.12	0.01
Vasoactive intestinal peptide receptor 1	VIPR1	NM_004624	-3.76	0.01	-3.09	0.01
V-erb-b2 erythroblastic leukemia viral oncogene homolog 3 (avian)	ERBB3	NM_001982	-2.1	0.01	-2.04	0.01
Villin 1	VIL1	BC017303	-3.75	0.01	-2.31	0.01
Vitamin D (1,25-dihydroxyvitamin D3) receptor	VDR	AA454701	-2.42	0.01	-2.23	0.01
V-set and immunoglobulin	VSIG2	NM_014312	-2.56	0.01	-2.14	0.01

domain containing 2						
WAS protein family homolog 3 pseudogene	WASH3P	AI628311	-3.53	0.01	-2.15	0.01
WD repeat domain 78	WDR78	AI989799	-2.74	0.01	-3.04	0.01
Wiskott-Aldrich syndrome-like	WASL	BE504979	-3.55	0.01	-2.66	0.01
WSC domain containing 1	WSCD1	AB011095	-2.62	0.01	-2.73	0.01
Zinc finger and BTB domain containing 7C	ZBTB7C	AI278995	-2.26	0.01	-2.16	0.01
zinc finger CCCH-type containing 7B	-	NM_025013	-3.66	0.01	-2.06	0.01
Zinc finger protein 575	ZNF575	AK057129	-2.82	0.01	-2.67	0.01
Zinc finger protein 789	ZNF789	AI355709	-4.15	0.01	-2.36	0.01
Zinc finger protein 91	ZNF91	NM_003430	-3.78	0.01	-2.85	0.01
Zinc finger protein 91 homolog (mouse)	ZFP91	AA758013	-2.72	0.01	-2.06	0.01
zinc finger with KRAB and SCAN domains 1	-	BG761185	-2.54	0.01	-2.04	0.01
Zinc finger, AN1-type domain 6	ZFAND6	AA741493	-4.41	0.01	-2.32	0.01
Zinc finger, DHHC-type containing 11	ZDHHC11	NM_024786	-3	0.02	-2.07	0.02
Zinc finger, DHHC-type containing 21	ZDHHC21	AK026322	-2.81	0.01	-2	0.01
Zinc finger, DHHC-type containing 23	ZDHHC23	AW003367	-2.84	0.01	-2.29	0.01

Supplementary Table 5. Ingenuity network analysis of pediatric CD

ID	Molecules in Network	Score	Focus Molecules	Top Functions
1	C8, CCL2, CD274, CFB, CHEMOKINE, CXCL1, CXCL2, CXCL3, CXCL5, CXCL6, CXCL9, CXCL10, CXCL11, Ferritin, Ifn, IFN alpha/beta, Ifn gamma, IFN TYPE 1, Ifnar, IL-17f dimer, IL17a dimer, IL17R, LCN2, lymphotoxin-alpha1-beta2, NFkB (complex), NfkB-RelA, OLFM4, PCSK1, PI3, S100A8, SLC3A1, SLC6A14, TFPI2, Tlr, Tnf	37	19	Cell-To-Cell Signaling and Interaction, Gastrointestinal Disease, Hepatic System Disease
2	Casein, CHI3L1, COL1A2, COL4A1, COL6A3, collagen, Collagen Alpha1, Collagen type I, Collagen type III, Collagen type IV, Collagen(s), Cyclooxygenase, CYR61, Elastase, Eotaxin, ERK1/2, Fibrin, FN1, Igfbp, IGFBP5, IL-1R, Laminin, Mmp, MMP1, MMP3, MMP10, NID1, Pdgf (complex), PSAT1, SAA, STAT1/3/5 dimer, Tgf beta, TIMP1, TMEM158, VCAN	27	15	Connective Tissue Disorders, Genetic Disorder, Cardiovascular Disease
3	ALDH1A2, ALDH7A1, C3, C11orf82, CCRL2, CFI, CFP, CKAP2, CLDN1, CLDN3, CTSZ, CX3CR1, CYSLTR2, DUOX2, DUOXA2, EMR2, GCHFR, GPR146, GPR109B, GRB2, HNF4A, HRH1, IFNG, IKBIP, PEG10, PHLDA1, PLA2G7, PLA2G12B, PRAP1, SEC24D, SLC16A1, SLC23A1, TP53, TPO, tretinoin	26	15	Cell Cycle, Genetic Disorder, Inflammatory Disease
4	ABCG2, ACSL4, Alp, C4BPA, CCRN4L, CDHR1, COL16A1, CTSZ, FKBP11, FOXJ2, GPR109B, HAPLN1, HAS1, Histone h4, HMGA1, HMGCS2, HNF1A, HSPA13, IGF1, IL13RA2, IL17B, KDELR3, KRT12, LOXL2, NNMT, oleic acid, P2RY6, PMP2, PPARG, SGK2, SLC20A1, TGFB1, TNF, VNN1, XBP1	25	14	Lipid Metabolism, Molecular Transport, Small Molecule Biochemistry
5	ABCB1, ABCC3, ABCG2, Adaptor protein 1, Akt, Angiotensin II receptor type 1, ANK3, Ap1, Creb, Cyclin A, CYP27B1, CYP2C9, Fibrinogen, hCG, HSD17B2, IL1B, N-cor, Ncoa-Nr1i2-Rxra, Ncoa-Nr1i3-Rxra, Nfat (family), p70 S6k, PCDH7, PDGF BB, PHLDA1, PHLPP2, PXR ligand-PXR-Retinoic acid-RXRα, Rap1, Rxr, SCD, SLC20A1, Smad, SOD2, TSH, VitaminD3-VDR-RXR, WISP1	24	15	Drug Metabolism, Molecular Transport, Small Molecule Biochemistry
6	ADAM17, ANGPTL3, ANPEP, APCDD1, APOA5, AQP8, beta-estradiol, C10orf116, cholesterol, DDR1, ERBB3, FBLN2, FBN1, FCRL5, HDL-cholesterol, HSD11B1, IER2, IGF1R, IGFBP5, KDELR3, MBL2, MEP1B, NEBL, PLTP, PTPN6, pyridoxine, SAA1, SERPINA3, SGMS1, SLC23A3, SLPI, SPINK4, TFPI, triacylglycerol lipase, ZYX	21	12	Lipid Metabolism, Molecular Transport, Small Molecule Biochemistry
7	ADM, APOBEC3B, Fc gamma receptor, FCGR1A, FCGR3B, Gm-csf, GOT, Growth hormone, Hemoglobin, Hsp27, Hsp70, IFN Beta, IgG, IgG2a, IL1, IL8, IL12 (complex), Immunoglobulin, Interferon alpha, LDL, LPL, MHC Class II (complex), MZB1,	16	10	Cellular Movement, Hematological System Development

	NFkB (family), NfkB1-RelA, Nos, NOS2, P38 MAPK, PI3K (complex), Pro-inflammatory Cytokine, PTGS2, SOCS3, Sod, sphingomyelinase, STAT5a/b			and Function, Hematopoiesis s
8	ADCY, Caspase, CCRL1, CDH11, Cg, CH25H, Cytochrome c, ELL2, ERK, Focal adhesion kinase, FSH, GBP1, Gpcr, HBB, Histone h3, HSD11B1, Ige, IKK (complex), IL12 (family), Insulin, Jnk, Lh, Mapk, Mlc, MS4A2, Pka, Pkc(s), PLC, Rac, Ras, RNA polymerase II, STAT, TYRP1, Vegf, ZDHHC11	15	10	Cell Morphology, Cellular Compromise, Cellular Development

Supplementary Table 6. Ingenuity network analysis of pediatric UC

ID	Molecules in Network	Score	Focus Molecules	Top Functions
1	Beta Arrestin, CALCRL, CCR6, CCR10, CCRL1, CXCR6, CXCR7, CYSLTR1, ELTD1, EMR2, F2RL2, FFAR2, FPR3, FZD2, Gpcr, GPR19, GPR65, GPR84, GPR85, GPR110, GPR126, GPR128, GPR180, GPR183, GPR109B, GPR137B, HTR4, LPHN3, Pik3r, PTGDR, PTGFR, RAMP3, Relaxin, SUCNR1, VIPR1	39	31	Cellular Movement, Cell Signaling, Nucleic Acid Metabolism
2	ADAMTS1, ARL4C, CHI3L1, COL12A1, COL15A1, COL1A2, COL3A1, COL4A1, COL4A2, COL5A1, COL5A2, COL6A2, COL6A3, collagen, Collagen type III, CTSK, DUSP7, EFEMP1, FBLN1, FBN1, FGFBP1, JPH1, LAIR2, Mapk, MFAP5, NNMT, PCSK6, Pdgf Ab, PPBP, STK38, Tenascin, TMEM158, TNC, VCAN, VWF	37	30	Connective Tissue Disorders, Genetic Disorder, Dermatological Diseases and Conditions
3	ACOX1, ADAMTS9, CARD6, CBR3, CLEC7A, CST7, E2F7, EHF, EOMES, FOXF1, G0S2, Gpd, IL1/IL6/TNF, IL13RA2, LY96, lymphotoxin-alpha1-beta2, MEOX1, MMRN1, NFkB (complex), NFKBIZ, NR2C2, peptidase, PPARα-RXRα, PRDX4, S100P, SLC3A1, SLC6A14, ST8SIA1, STAP2, TNFAIP8, TNFRSF17, VNN1, VSNL1, ZC3H12A, ZFAND6	35	29	Amino Acid Metabolism, Molecular Transport, Small Molecule Biochemistry
4	3',5'-cyclic-nucleotide phosphodiesterase, AKAP, AKAP1, AKAP12, AKAP2/PALM2-AKAP2, Akt, AMY1A (includes others), CADM1, CDH13, CRTAM, ELOVL5, LETM1, MAGI1, MCAM, NOX4, PCK1, Pde, Pde4, PDE10A, PDE4B, PDE6A, PDE8A, PDK1, PDLIM4, PLN, Ppp2c, PRKAC, PRKAR2B, PROK2, PTPN13, RBPMS, ST8SIA4, SULF1, THBS2, TNFAIP6	33	28	Cardiovascular Disease, Cell Signaling, DNA Replication, Recombination, and Repair
5	Acid Phosphatase, ACP5, ACPP, ADM, APOL1, ARFGAP3, C1orf38, CAPN3, CD80/CD86, Cebp, CECR1, CLEC5A, DEFB1, FAAH, FLRT2, GBP4, GBP5, HAVCR2, HBA1/HBA2, HBB, HBD, Hemoglobin, IFNG, IL-2R, KMO, KYNU, MNDA, Par, RAB38, Serine Protease, SH2D1A, SLC6A6, STX11, UBD, VAMP4	33	28	Amino Acid Metabolism, Small Molecule Biochemistry, Genetic Disorder
6	AMT, cldn, CLDN1, CLDN2, CLDN8, COL18A1, COL1A1, CPM, CTGF, CYR61, EFNB2, ENTPD5, F3, F2R, Fibrinogen, FLI1, GLDC, GPIIB-IIIA, Granzyme B-Perforin-SRGN, Gα12/13, IGFBP5, IGFBP7, INADL, INHBA, ITGA2, KCNJ15, LOX, NID2, PECAM1, PF4, SERPINB9, Smad2/3-Smad4, SRGN, TJP2, Vla-4	32	28	Cell-To-Cell Signaling and Interaction, Cardiovascular System Development and Function, Organismal Development
7	ADAM9, ANTXR1, Collagen type IV, Complement component 1, DPP10, Elastase, ERK1/2, GAL, Integrin alpha V beta 3, KCNN3, LAMA1, LAMC1, LAMC2, Laminin, Laminin1, LIMS1, LPP, Mac1, MMP7, MMP12, MMP19, MT1F, MTUS1, NID1, PARVB, PCBP2, Potassium Channel, PRRX1,	29	26	Post-Translational Modification, Cell-To-Cell Signaling and Interaction,

	PTPRF, QKI, RBP4, REG3A, SERPING1, TFF2, TINAG			Tissue Development
8	ANGPT2, Ap1, BHLHE40, BLVRA, BTG3, Calcineurin protein(s), CEP170, DLG1, ELK3, EPHX2, ETS, EZR, FJX1, Glutathione peroxidase, GNLY, GPX7, GPX8, HAS2, hCG, HSD17B3, HSD3B1, IL11, Lh, MGST1, NMDA Receptor, PBK, PDCD4, PHLPP2, RAB31, Sapk, SLC4A4, SOD2, SRC, TIPIN, TM4SF1	29	26	Cellular Growth and Proliferation, Hematological System Development and Function, Hematopoiesis
9	Alpha tubulin, AQP3, AREG/AREGB, Beta Tubulin, C2, C12orf23, C2-C4b, C3-Cfb, CD55, CFB, CFI, CH25H, CHST11, CHSY1, DNAJB9, DRAM1, EPB41L4B, FILIP1L, FSH, GJA1, GJA5, GJC1, HDGFRP3, IP6K2, MDFIC, MIR1, MIR124 (human), OSBPL8, PLA1A, RAB8B, RAVER2, RBMS1, SH3PXD2B, SLC22A5, TUBB6	28	28	Cardiovascular Disease, Hematological Disease, Renal Thrombosis
10	BAG2, CARD16, CASP1, Caspase 3/7, CD80, CHPT1, CSTA, ERO1LB, IFN TYPE 1, Iga, IgG2b, IGHM, IL24, IL12 (family), KDELR3, KLK10, LGALS1, MHC, PI3, PIM2, POU2AF1, PRDM1, Pro-inflammatory Cytokine, RGS18, SAMSN1, SATB2, SDF2L1, SELM, TDO2, TH1 Cytokine, Tlr, TREM1, TXNDC5, XBP1, YIPF5	28	26	Hematological System Development and Function, Humoral Immune Response, Tissue Morphology
11	Alp, ANK3, ASAP1, BMP, BMP6, CHP2, CHRDL2, CTNNβ-TCF/LEF, CYP39A1, EPHA2, EXOC5, Fgf, FGF9, Fgfr, FGFR2, FGFR3, Focal adhesion kinase, HOXA5, Hspg, KAL1, LPCAT1, p85 (pik3r), RUNX2, SDC2, SLC9A2, Smad, Smad1/5/8, SMARCA1, SORD, SPP1, STC1, STS, TPSAB1/TPSB2, TWIST1, WNT5A	27	25	Tissue Development, Cellular Development, Organismal Injury and Abnormalities
12	ADCY, ADCYAP1, C-C CHEMOKINE RECEPTOR, C-X-C CHEMOKINE RECEPTOR, CCL2, CCL3, CCL4, CCL11, CCL18, Ccr, CCR1, CCR2, CCR5, CHEMOKINE, CHEMOKINE RECEPTOR, CXCL1, CXCL2, CXCL3, CXCL5, CXCL9, CXCL11, CXCR2, DDHD1, EDNRB, FPR1, FPR2, G protein alphai, Gi-coupled receptor, Gs-coupled receptor, IL17A, NOD2, RGS5, S100A8, S100A9, Tnf	27	25	Cellular Movement, Hematological System Development and Function, Immune Cell Trafficking
13	AChR, AQP9, ASPN, Calpain, CHRNA5, DCLK1, DDX17, EDN1, EDNRA, Endothelin, ERLEC1, GBP1, GOT, Histone h4, HSD17B2, IFIT3, IL1RN, IRF4, LTBP2, NRG, NRG1, p70 S6k, RBM39, SEL1L, SELE, SLC2A3, SLFN12, Smad2/3, TCF4, Tgf beta, TGFB2, TNFSF11, TSPAN5, VDR, VitaminD3-VDR-RXR	26	25	Cellular Development, Embryonic Development, Cardiovascular Disease
14	ABCB1, AGT, ANXA3, Calmodulin, CD3, CD44, Ck2, Cytokeratin, DDAH2, DECR1, FAM69A, FGF2, HADHA, HDHD3, IGKV1D-8, IRS1, KRT7, KRT12, KRT6A, KRT6B, Mediator, Neurotrophin, Nos, PPFIBP1, Proinsulin, SEC24A, SELENBP1, SLC18A2, SLC7A11, SRRM2, SSR1, SSR4, TRIP13, Voltage Gated Calcium Channel, WASL	26	26	Cellular Movement, Skeletal and Muscular System Development and Function,

				Tissue Development
15	ALT, ARHGEF3, BUB1, CDK3, CDKN3, CENPA, CRISPLD2, Cyclooxygenase, Cytochrome p450, Growth hormone, HLA-DMA, HLA-DRA, HSP, IDO1, IL6, IL33, Immunoglobulin, LAG3, Mhc class ii, MHC Class II (complex), Mhc2 Alpha, Na+,K+ -ATPase, NCF2, NMU, NUCB2, OSBPL1A, PLP1, S100A12, SAA, SAA2, SAA4, SLC39A6, SNX10, SPC25, WARS	25	24	Inflammatory Disease, Cellular Growth and Proliferation, Hematological System Development and Function
16	ACVR1, Alpha Actinin, Alpha catenin, Angiotensin II receptor type 1, C13orf15, Cadherin, CALU, CAV2, Caveolin, CDH3, CDH11, CDK14, CFL2, Collagen Alpha1, Collagen type I, CSRP2, DPYD, Eotaxin, FAP, FPR, G protein, G-Actin, IL10RA, JUB, LATS2, LMO7, LPHN2, PLAUR, PRAP1, PRKG2, RHOJ, SERPINB5, SRPX2, TFAP2C, TGFBI	25	24	Cellular Movement, Hematological System Development and Function, Hypersensitivity Response
17	ALDH1A2, C2orf88, CCL18, CCL23, CD274, CLEC4A, CLEC7A, CMKLR1, CTSC, CYSLTR1, DEFA1 (includes others), ERO1L, FMO3, Gzmc, HLX, IL2, IL5, IL13, IL15, IL21R, IRAK3, MAOA, NCR3, NMU, PDE4B, PHLDA1, PLXNC1, PRDX4, PRKAR1A, RAB33A, SLC26A6, SLC7A5, TLR1, TSPAN7, ZBED2	25	24	Cell-mediated Immune Response, Cellular Movement, Hematological System Development and Function
18	A1CF, ACACB, ADAM12, ADAM19, APOBEC3B, APOBEC3F, APOBEC3G, CDA, CELF2, CETP, CKB, CLIC4, Creatine Kinase, cytidine deaminase, EDN3, Endothelin Receptor, EPHA3, ERK, GALNT2, HDL, HDL-cholesterol, Importin alpha, LDL-cholesterol, LIPG, LOXL1, LPL, LRRC8C, Metalloprotease, NCOR-LXR-Oxysterol-RXR-9 cis RA, PLCL1, PNLIPRP2, PROCR, SGMS1, SPRED1, triacylglycerol lipase	25	24	Lipid Metabolism, Small Molecule Biochemistry, Nucleic Acid Metabolism
19	ADH1C, ANGPTL2, ANP32E, ARNTL2, BCL2A1, C/ebp, HELLS, IL1B, INSIG1, KIAA1199, Ldh, LDL, LRP, LRP8, MT1G, Notch, NR4A2, NRCAM, Pcsk, PCSK1, PCSK7, PCSK9, Pias, PLD1, PLXNA2, PMAIP1, PTGDS, S100A11, Secretase gamma, SLC20A1, Sod, Sphk, THBD, Vegf, VEGFC	24	24	Amino Acid Metabolism, Molecular Transport, Small Molecule Biochemistry
20	Adaptor protein 1, ANPEP, ANXA1, AP1S3, BNIP3, CD38, Cytochrome c, ENaC, Estrogen Receptor, G protein alpha, GC-GCR dimer, GNA14, Hsp27, HSPB3, Ikb, IKK (complex), IL8, IL1A, MEP1B, NEDD4L, OLFML3, OTUD7B, PARP, PTGS2, S100A2, SCNN1B, SLPI, SNCAIP, SPG20, TLR2, Tnf receptor, TNFRSF6B, TRIB2, Ubiquitin, ZG16B	24	23	Endocrine System Development and Function, Lipid Metabolism, Molecular Transport
21	C9orf3, CDH11, CDHR1, CITED2, COL4A1, CPXM1, DFNA5, DKK3, ELN, FBN1, FCN1, FNDC3B, GLI2, GYG1, HOXB5, HS6ST2, HSD17B6, IGFBP7, ITGA2, MAP4, MSRB3, MYCN, PDZK1IP1, PHGDH,	24	23	Cellular Movement, Cell-To-Cell Signaling and

	Pyruvate kinase, S100A10, SDC2, SPAG4, TAGLN, TERC, TGFB1, TPM1, TPST2, TRIM7, ZEB2			Interaction, Cellular Assembly and Organization
22	20s proteasome, 26s Proteasome, ACVR1C, alcohol group acceptor phosphotransferase, ATXN1, DLGAP5, ERBB3, FADS1, FLNB, GZMB, Hsp70, Hsp90, IL21R, Insulin, Interferon alpha, MAP3K8, MDM2, NBPF11 (includes others), NDRG2, NEK2, NOS2, P110, Pka catalytic subunit, PSMB9, PYHIN1, Ras homolog, RGS2, RNA polymerase II, RPAP3, SPEN, SPINK4, STAT1, TRPV1, UQCRC2, ZC3H7B	23	24	Infection Mechanism, Cell Death, Infectious Disease
23	ACADS, CD1E, CHST2, CXCL2, CXCL3, HAPLN1, HAS2, hyaluronic acid, hydroquinone, IRAK3, LAYN, LY96, LYVE1, MAST4, MMP12, MUC20, NCAN, NQO2, OLFML2B, PIGA, PIGB, PIGH, PIGQ, PLXNB2, PXMP2, RND1, SCFV, SLC1A4, SLPI, SMAD1, SMAD1/5, TLR2/TLR4, TNF, TNFAIP6, VCAN	22	22	Carbohydrate Metabolism, Cellular Movement, Hematological System Development and Function
24	ADAMTS5, ASPA, Casein, Chymotrypsin, CLCN2, COL14A1, Collagen(s), DCN, FGF7, HTRA1, Kallikrein, Mmp, MMP1, MMP2, MMP3, MMP9, MMP10, MMP28, NAMPT, OSMR, PCOLCE, Plasminogen Activator, PLAU, SERPINA1, SERPINA3, SERPINE2, STAT1/3/5, STAT1/3/5 dimer, TCF/LEF, TFPI, TFPI2, Timp, TIMP1, TIMP3, Trypsin	22	24	Connective Tissue Disorders, Genetic Disorder, Inflammatory Disease
25	ABCC4, AMPK, CAR ligand-CAR-Retinoic acid-RXRα, Cbp/p300, Creb, CYP1B1, CYP2B6, CYP2C9, CYP3A5, CYP4F12, CYP4X1, DUSP4, EAF2, ELL2, GAS1, Gsk3, Holo RNA polymerase II, HSD3B2, MITF, PDK4, PFKFB3, PPARGC1A, PRKAA, RPS6KA2, Rsk, SCD, sphingomyelinase, SRSF4, Stat1 dimer, TCEA3, TFEC, unspecific monooxygenase, Wnt, WNT4, WNT2B	21	23	Endocrine System Development and Function, Small Molecule Biochemistry, Drug Metabolism

Supplementary Table 7. GSE9686 pediatric CD promoter analysis of up regulated genes

**Promoter database**	**TFBS name**	**TFBS number**	**TFBS fold**	**p**
**JASPAR**	**RELA**	**55**	**5.1**	**0.0004**
	**NF-kappaB**	**60**	**4.9**	**0.0008**
	**IRF2**	**43**	**4.1**	**0.0087**
	**Evi1**	**41**	**3.2**	**0.0569**
	NFYA	36	3.1	0.0714
	**IRF1**	**34**	**3**	**0.0935**
	**TAL1-TCF3**	**36**	**2.9**	**0.1003**
	**ELF5**	**42**	**2.8**	**0.1176**
	SRF	34	2.8	0.1293
	NFKB1	32	2.7	0.1374
**Transfac**	**V$GATA_C**	**72**	**7.6**	**0.0026**
	**V$STAT3_01**	**75**	**7.2**	**0.0057**
	**V$ISRE_01**	**71**	**6**	**0.0366**
	**V$HLF_01**	**74**	**5.9**	**0.0441**
	**V$EVI1_04**	**57**	**5.8**	**0.0445**
	**V$NFKAPPAB65_01**	**59**	**5.8**	**0.0455**
	V$OCT1_04	62	5.7	0.0518
	V$OLF1_01	57	5.7	0.0531
	V$IRF2_01	79	5.7	0.0536
	V$CEBPA_01	70	5.6	0.0626

Supplementary table 8. GSE9686 pediatric CD promoter analysis of down regulated genes

**Promoter database**	**TFBS name**	**TFBS number**	**TFBS fold**	**p**
**JASPAR**	**Lhx3 (MA0135)**	**17**	**10.6**	**0.001**
	**MEF2A**	**13**	**7.5**	**0.001**
	**HNF1A**	**13**	**7.2**	**0.0017**
	**Nobox**	**16**	**6.9**	**0.0029**
	Pax4	10	5.6	0.0185
	**NR2F1**	**10**	**4.8**	**0.0468**
	FOXF2	7	4.3	0.0806
	**Foxa2**	**9**	**4.3**	**0.0445**
	**NR3C1**	**9**	**4.3**	**0.0848**
	Pdx1	9	4.3	0.097
**Transfac**	V$EVI1_01	22	11.3	0.0029
	**V$MEF2_03**	**19**	**10.4**	**0.0074**
	V$SREBP1_01	17	10.4	0.0075
	**V$HNF1_01**	**19**	**10.1**	**0.0101**
	V$MEF2_04	21	10	0.0111
	**V$EVI1_04**	**16**	**9.7**	**0.0136**
	**V$EVI1_05**	**18**	**9.6**	**0.0151**
	V$S8_01	17	9.6	0.0152
	**V$OCT1_04**	**17**	**9.3**	**0.0194**
	V$XBP1_01	21	8.9	0.0266
	V$OCT1_05	13	8	0.0333
	V$OCT1_02	15	7.9	0.0357

Supplementary Table 9. GSE10616 pediatric CD promoter analysis of up regulated genes

Promoter database	TFBS name	TFBS number	TFBS fold	p
**JASPAR**	NF-kappa B	85	5.3	0.0007
	RELA	69	4.9	0.0018
	IRF2	51	3.7	0.0315
	ELF5	70	3.6	0.0367
	IRF1	49	3.3	0.0681
	Evi1	54	3.3	0.0695
	Pax4	44	3.2	0.0807
	FOXD1	55	2.9	0.1251
	TAL1-TCF3	46	2.9	0.1311
	Lhx3 (MA0135)	35	2.8	0.1391
**Transfac**	V$STAT3_01	104	7.6	0.0017
	V$HLF_01	111	6.7	0.0093
	V$OCT1_Q6	100	6.4	0.015
	V$NFKAPPAB65_01	80	6.1	0.0271
	V$EVI1_01	91	6.1	0.0273
	V$ISRE_01	89	5.8	0.0415
	V$GATA_C	71	5.7	0.0421
	V$NFKAPPAB_01	84	5.7	0.0432
	V$EVI1_04	72	5.7	0.0472
	V$CAAT_C	84	5.2	0.0792

Supplementary table 10. GSE10616 pediatric CD promoter analysis of down regulated genes

**Promoter database**	**TFBS name**	**TFBS number**	**TFBS fold**	**p**
**JASPAR**	MEF2A	12	7.5	0.000
	HNF1A	11	6.5	0.001
	Lhx3 (MA0135)	9	6.1	0.0025
	Nobox	11	5.1	0.0147
	Evi1	10	5.1	0.0156
	SRF	9	4.7	0.0268
	NR2F1	9	4.7	0.0275
	Foxa2	9	4.6	0.0297
	NR3C1	9	4.6	0.0312
	HNF4A	8	4.3	0.0454
**Transfac**	V$HNF1_01	17	9.7	0.0057
	V$MEF2_03	16	9.5	0.0076
	V$EVI1_04	14	9.2	0.0101
	V$OCT1_04	15	8.9	0.0141
	V$EVI1_05	15	8.6	0.0177
	V$CDP_02	14	8.3	0.0234
	V$RSRFC4_01	14	8.3	0.0251
	V$MEF2_04	16	8.2	0.0272

Supplementary Table 11. The over represented transcription factor binding sites in the up regulated genes in pediatric UC inflamed tissue

TFBS Data base	TFBS name	TFBS s number	TFBS s fold	p
JASPAR TFBSs	IRF2	209	3.9	0.0181
	Pax4	207	3.9	0.0201
	IRF1	205	3.6	0.0363
	NFYA	197	3.4	0.0486
	RELA	161	3	0.0945
	FOXF2	141	2.9	0.1068
	NF-kappa B	179	2.9	0.1077
	SRY	150	2.8	0.1289
	Foxq1	171	2.7	0.1475
	Evi1	169	2.7	0.1565
Transfac TFBSs	V$AP1FJ_Q2	501	9.4	0.0001
	V$GATA_C	305	6.4	0.0079
	V$OCT1_04	331	6.1	0.0143
	V$STAT3_01	285	5.4	0.0418
	V$IRF1_01	274	5.3	0.0494
	V$HLF_01	333	5.2	0.0547
	V$CHOP_01	241	5.1	0.0697
	V$MEF2_01	299	5	0.0757
	V$EVI1_04	235	4.8	0.0982
	V$IRF2_01	331	4.8	0.1034

Supplementary table 12. The overpresented transcription factor binding sites in the down regulated genes in pediatric UC inflamed tissue

TFBS database	TFBS name	TFBS s Number	TNBSs fold	p
JASPAR TFBSs	HNF1A	93	4	0.0087
	Lhx3 (MA0135)	75	3.6	0.0203
	Pax4	82	3.6	0.0245
	MEF2A	74	3.3	0.0434
	NR2F1	81	3	0.0739
	IRF2	69	3	0.077
	Nobox	88	2.9	0.0901
	RORA_1	67	2.9	0.0981
	Foxa2	76	2.8	0.1107
	Lhx3 (MA0134)	72	2.8	0.1129
Transfac TFBSs	V$GATA_C	157	7.6	0.0012
	V$AP1FJ_Q2	150	6.5	0.011
	V$EVI1_04	134	6.3	0.0156
	V$HNF1_01	146	6	0.0265
	V$MEF2_01	147	5.7	0.0426
	V$NRSF_01	122	5.5	0.0539
	V$OCT1_04	129	5.5	0.0566
	V$OCT1_05	124	5.5	0.0585
	V$CLOX_01	128	5.4	0.06
	V$RSRFC4_01	124	5.3	0.0751

Supplementary Table 13. GSE9686 pediatric UC KEGG pathway analysis

KEGG Pathway	List	Gene Set	z-score
**Cytokine-cytokine receptor interaction**	**68**	**263**	**4.27**
Pathways in cancer	67	323	2.2
Focal adhesion	48	196	3.14
**Chemokine signaling pathway**	42	182	2.49
Neuroactive ligand-receptor interaction	33	311	-2.84
**Cell adhesion molecules (CAMs)**	32	126	2.77
**ECM-receptor interaction**	27	82	4.09
**Toll-like receptor signaling pathway**	26	101	2.57
**Hematopoietic cell lineage**	23	83	2.82
**Leishmaniasis**	21	65	3.5
**Malaria**	20	49	4.65
**Complement and coagulation cascades**	19	67	2.67
**Alzheimer's disease**	14	156	-2.54
**Huntington's disease**	14	169	-2.89
**Steroid hormone biosynthesis**	13	43	2.47
**Bladder cancer**	12	41	2.24
**Graft-versus-host disease**	10	33	2.17
**Biosynthesis of unsaturated fatty acids**	9	20	3.47
**Oxidative phosphorylation**	8	120	-2.91
**Olfactory transduction**	7	97	-2.46
Renin-angiotensin system	7	17	2.77
One carbon pool by folate	6	17	2.11
**Ribosome**	5	85	-2.64
**Sulfur metabolism**	5	13	2.16
**RIG-I-like receptor signaling pathway**	3	70	-2.75
Aminoacyl-tRNA biosynthesis	2	41	-2
Notch signaling pathway	2	46	-2.21

The bold pathways (number is 20) are dysregulated in both sets of pediatric UC microarray data.

Supplementary Table 14. GSE10616 pediatric UC KEGG pathway analysis

KEGG Pathway	List	Gene Set	z-score
Cytokine-cytokine receptor interaction	52	263	5.04
Cell adhesion molecules (CAMs)	29	126	4.64
Chemokine signaling pathway	29	182	2.43
ECM-receptor interaction	22	82	4.86
Complement and coagulation cascades	21	67	5.6
Toll-like receptor signaling pathway	21	101	3.4
Leishmaniasis	20	65	5.36
Malaria	20	49	6.95
Hematopoietic cell lineage	19	83	3.71
Steroid hormone biosynthesis	14	43	4.73
Drug metabolism - cytochrome P450	13	58	2.97
PPAR signaling pathway	13	68	2.33
Arachidonic acid metabolism	12	51	3.05
Intestinal immune network for IgA production	11	44	3.15
Retinol metabolism	11	48	2.82
Graft-versus-host disease	10	33	3.72
Bladder cancer	9	41	2.4
Tryptophan metabolism	9	37	2.75
Type I diabetes mellitus	9	37	2.75
Alzheimer's disease	8	156	-2.23
Huntington's disease	8	169	-2.49
Allograft rejection	7	32	2.1
Asthma	6	26	2.09
Biosynthesis of unsaturated fatty acids	6	20	2.85
Glycosaminoglycan biosynthesis - chondroitin sulfate	6	21	2.7
Neurotrophin signaling pathway	5	125	-2.4
Oocyte meiosis	5	109	-2.04
Sulfur metabolism	5	13	3.29
Spliceosome	4	121	-2.62
ErbB signaling pathway	3	84	-2.09
Olfactory transduction	3	97	-2.41
Oxidative phosphorylation	3	120	-2.9
Long-term potentiation	2	69	-2.08
RIG-I-like receptor signaling pathway	2	70	-2.1
Vitamin B6 metabolism	2	5	2.15
Colorectal cancer	1	61	-2.27
Non-small cell lung cancer	1	54	-2.09
Ribosome	1	85	-2.83
RNA degradation	1	56	-2.14

Supplementary Table 15. T cells transfer colitis model progressively up regulated genes promoter analysis

	TFBS name	TFBSs number	TFBSs fold	P
JASPAR	IRF2	120	4.5	0.0034
	IRF1	113	3.9	0.0142
	RELA	105	3.8	0.015
	NF-kappaB	116	3.7	0.0201
	ELF5	140	3.7	0.0205
	Cebpa	90	2.9	0.0921
	NFKB1	83	2.8	0.1191
	NFYA	81	2.8	0.1258
	FOXF2	66	2.7	0.1392
	Lhx3 (MA0134)	81	2.7	0.1397
Transfac	V$AP1FJ_Q2	206	7.6	0.0004
	V$GATA_C	147	6.1	0.0121
	V$NFY_C	146	6.1	0.0124
	V$NFKAPPAB65_01	156	6	0.0131
	V$STAT3_01	160	6	0.0141
	V$OCT1_05	159	6	0.0151
	V$HLF_01	192	5.9	0.0154
	V$NFKAPPAB_01	170	5.9	0.0162
	V$OCT1_Q6	179	5.9	0.017
	V$IRF1_01	145	5.5	0.0316

Supplementary table 16. T cell colitis model progressively down regulated genes promoter analysis

	TFBS name	TFBSs number	TFBSs fold	p
JASPAR	Pax4	148	4.5	0.0051
	HNF1A	149	4.5	0.0054
	Lhx3 (MA0135)	125	4.3	0.0089
	Lhx3 (MA0134)	127	3.5	0.0432
	MEF2A	108	3.4	0.0511
	SRY	102	3.1	0.0855
	Nobox	130	3.1	0.0918
	IRF2	97	3	0.1025
	Cebpa	99	2.7	0.162
	Foxq1	103	2.7	0.1681
Transfac	V$AP1FJ_Q2	285	8.7	0.0002
	V$OCT1_04	229	6.9	0.0093
	V$HNF1_01	236	6.8	0.0097
	V$EVI1_04	199	6.6	0.0138
	V$GATA_C	192	6.6	0.0148
	V$CHOP_01	180	6.2	0.0274
	V$S8_01	195	6	0.0333
	V$RSRFC4_01	193	5.8	0.046
	V$OCT1_05	186	5.8	0.0467
	V$MEF2_03	179	5.4	0.0771

Supplementary table 17. DSS Colitis model of promoter of progressively up regulated genes

	TFBS name	TFBSs number	TFBSs fold	p
JASPAR	IRF2	200	3.9	0.0242
	Pax4	194	3.7	0.0306
	IRF1	188	3.4	0.058
	MEF2A	166	3.3	0.0649
	Foxa2	186	3.1	0.0922
	Cebpa	179	3.1	0.0939
	FOXI1	148	2.9	0.1095
	FOXF2	135	2.9	0.1222
	Foxd3	171	2.8	0.134
	Lhx3 (MA0134)	157	2.7	0.1512
Transfac	V$GATA_C	324	7	0.0047
	V$AP1FJ_Q2	361	7	0.0049
	V$HLF_01	363	5.9	0.0315
	V$SRY_01	265	5.8	0.0361
	V$MEF2_01	328	5.6	0.044
	V$OCT1_04	297	5.6	0.0457
	V$EVI1_04	267	5.6	0.0464
	V$IRF1_01	275	5.5	0.0546
	V$RSRFC4_01	289	5.5	0.056
	V$MEF2_03	289	5.5	0.056

Supplementary table 18. DSS colitis model progressively down regulated genes promoter analysis

	TFBS name	TFBS number	TFBSs fold	p
JASPAR	HNF1A	100	5.1	0.0016
	Pax4	84	4.3	0.0093
	IRF2	71	3.7	0.0353
	SRY	70	3.6	0.0424
	HLF	74	3.5	0.0458
	Lhx3 (MA0135)	61	3.5	0.0473
	Foxa2	78	3.4	0.0549
	FOXF2	59	3.3	0.063
	Cebpa	72	3.3	0.0712
	IRF1	68	3.2	0.0749
Transfac	V$AP1FJ_Q2	229	11.7	0.000
	V$GATA_C	165	9.5	0.0006
	V$EVI1_04	144	8	0.0067
	V$HNF1_01	162	7.9	0.0084
	V$CHOP_01	128	7.3	0.0171
	V$OCT1_05	138	7.2	0.0213
	V$OCT1_04	128	6.4	0.0512
	V$HFH2_01	138	6.2	0.0646
	V$RSRFC4_01	121	6.1	0.0738
	V$EVI1_01	127	6	0.0804
